# The Nature of Shared Cortical Variability

**DOI:** 10.1016/j.neuron.2015.06.035

**Published:** 2015-08-05

**Authors:** I-Chun Lin, Michael Okun, Matteo Carandini, Kenneth D. Harris

**Affiliations:** 1 UCL Institute of Neurology, University College London, London WC1N 3BG, UK; 2 UCL Institute of Ophthalmology, University College London, London EC1V 9EL, UK; 3 UCL Department of Neuroscience, Physiology, and Pharmacology, University College London, London WC1E 6DE, UK

## Abstract

Neuronal responses of sensory cortex are highly variable, and this variability is correlated across neurons. To assess how variability reflects factors shared across a neuronal population, we analyzed the activity of many simultaneously recorded neurons in visual cortex. We developed a simple model that comprises two sources of shared variability: a multiplicative gain, which uniformly scales each neuron’s sensory drive, and an additive offset, which affects different neurons to different degrees. This model captured the variability of spike counts and reproduced the dependence of pairwise correlations on neuronal tuning and stimulus orientation. The relative contributions of the additive and multiplicative fluctuations could vary over time and had marked impact on population coding. These observations indicate that shared variability of neuronal populations in sensory cortex can be largely explained by two factors that modulate the whole population.

## Highlights

• Response variability in V1 neuronal populations is largely shared across neurons
• Shared variability involves two factors: a multiplicative gain and an additive offset
• These two factors predict sensory responses of large populations on single trials
• They determine pairwise correlations and constrain information coding

## Teaser

Cortical responses are highly variable. Using large-scale recordings in V1, Lin et al. show that this variability is shared across neurons and involves two simple factors: multiplicative and additive. These factors shape the joint variability of large populations of neurons.

## Introduction

Repeated presentations of the same stimulus elicit highly variable responses in sensory cortex (Heggelund and Albus, 1978; Tolhurst et al., 1983; Vogels et al., 1989). This variability is correlated across neurons, so it cannot be easily removed by averaging across the population, and may thus place critical constraints on information transmission (Averbeck et al., 2006; Averbeck and Lee, 2006; Deweese and Zador, 2004; Shadlen and Newsome, 1998; Zohary et al., 1994). Understanding its nature can thus shed light on the circuit mechanisms and computations performed by the cortex in health and disease (Dinstein et al., 2015).

Cortical variability does not arise because neurons are intrinsically noisy. Indeed, cortical neurons can generate highly reliable spike trains (Mainen and Sejnowski, 1995). The variability of their responses is therefore more likely to arise from the variability of their synaptic inputs, reflecting cortical network dynamics (Carandini, 2004). Traditionally, variability has been studied in single neurons or neuronal pairs, but a full description requires understanding factors operating at the population level.

A clue toward understanding cortical variability comes from spontaneous activity patterns that the cortex produces in the absence of stimuli. These patterns share some features with responses evoked by sensory stimuli (Arieli et al., 1996; Kenet et al., 2003; Luczak et al., 2009, 2013; Ringach, 2009; Tsodyks et al., 1999): for instance, pairwise correlations measured during spontaneous activity can resemble those seen during sensory stimulation (Jermakowicz et al., 2009; Okun et al., 2012). Voltage-sensitive dye imaging experiments in visual cortex suggest that the interaction between spontaneous and evoked activity is additive: activity would be the sum of a deterministic sensory response and a stochastic pattern originating from networks that generate spontaneous activity (Arieli et al., 1996). Subsequent work showed that such an additive interaction could approximate the dependence of pairwise correlations on cortical state (Schölvinck et al., 2015). Other work suggests a more complex picture. In auditory cortex, responses to prolonged tone stimuli show similar fluctuating activity to those seen in silence (Luczak et al., 2013). Yet, sensory-evoked spikes do not occur independently of these fluctuations, as would be expected from addition, but occur together with them, suggesting that spontaneous fluctuations gate the representation of stimuli. In visual cortex, quantitative analyses suggest that the variability of single neurons and correlations of neuronal pairs are more consistent with a multiplicative gain change, whose gain factor fluctuates from trial to trial (Ecker et al., 2014; Goris et al., 2014).

Such multiplicative variability is consistent with what one might expect from top-down feedback from higher order cortices. By targeting layer 1, this feedback can change the gain with which neurons respond to activity in input layers (Larkum, 2013; Larkum et al., 1999). Top-down feedback might be involved in spatial attention (Armstrong and Moore, 2007; Moore and Armstrong, 2003), which can have a multiplicative effect on neuronal gain (Reynolds and Heeger, 2009). Yet, other evidence suggests that the influence of spatial attention is additive (Boynton, 2009; Buracas and Boynton, 2007; Murray, 2008; Thiele et al., 2009). Other modulatory effects, such as those seen in visual cortex during locomotion, appear to be both additive and multiplicative (Ayaz et al., 2013).

These observations raise multiple questions. If single-neuron variability is well-modeled by multiplicative gain changes, can a single, population-wide gain factor explain the coordinated fluctuations of the population? Are the additive and multiplicative models mutually exclusive or is there a common ground between them? And what are the effects of the multiplicative and additive fluctuations on the cortical code?

Here, we answer these questions by analyzing the trial-by-trial activity of large, simultaneously recorded neural populations in primary visual cortex (V1) of anesthetized cats and quietly awake mice. We find that cortical variability is best understood at the population level, where it can be described by a simple mathematical model comprising two sources of shared variability: multiplicative and additive. This model explained the structure of trial-to-trial population variability and captured the complex dependence of neuronal correlations on stimulus and neuronal tuning. Our results suggest that neither additive nor multiplicative variability alone forms a complete model of cortical variability; instead, a combination of the additive and multiplicative components that invest the whole population can explain much of the shared response variability in visual cortex.

## Results

**Figure 1:**
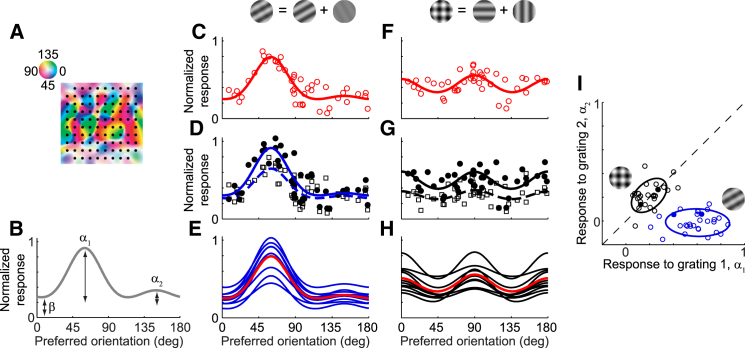
Recordings of Population Activity in Anesthetized Cat V1 (A) Layout of a 10-by-10 electrode array aligned to the underlying map of preferred orientations (adapted from Katzner et al., 2009). The electrode sites are 400 μm apart. (B) The function fitted to population responses is the sum of two circular Gaussians: one peaking at the orientation of grating 1 with amplitude *α*_1_, and one peaking at the orientation of grating 2 with amplitude *α*_2_. A baseline untuned response *β* provides an additive offset. (C) Population response averaged over ten presentations of a plaid with orientations 60° and 150° and contrasts 50% and 6%. Each circle shows the trial-averaged normalized firing rate of an orientation-tuned site, arranged by its preferred orientation. The firing rate is normalized by each site’s response to its optimal orientation at 100% contrast. The curve indicates the fit of the function in (B). (D) Two example single-trial population responses to the plaid shown in (C). A filled circle and an empty square represent the normalized firing rates of an orientation-tuned site on trials 1 and 2; the solid and dashed lines plot the fits of the function in (B) to population responses on trials 1 and 2. (E) Population responses to ten presentations of the plaid shown in (C) (blue); only fitted curves are shown for clarity. A red curve repeats the trial-averaged population response for comparison. (F–H) As in (C)–(E), but for a plaid with component gratings of orientations 0° and 90°, both at 50% contrast. (I) Variability in population responses measured by their response to grating 1, *α*_1_, and their response to grating 2, *α*_2_. The blue circles indicate responses to any plaids with contrasts 50% (grating 1) and 6% (grating 2); the black circles indicate responses to any plaids with contrasts 50% and 50%. Each open circle denotes population response on one trial; the four solid circles mark the four single-trial population responses shown in (D) and (G). For each component-contrast combination, the data were pooled across different component orientations. The ellipses show 1 SD contours of Gaussian fits (session 83-7-5, 45 orientation-tuned sites, plaid angle = 90°).

We first analyzed the activity of large neural populations in V1 of anesthetized cats (seven recording sessions from three neuronal populations in three cats). Neuronal responses were recorded from a 10-by-10 electrode array that covered a 16 mm^2^-region with a diversity of orientation preferences (Figure 1A). All spikes detected on a given site of the array were pooled, as they originated from neurons having similar preferred orientations (Katzner et al., 2009). Stimuli were contrast-reversing oriented gratings and plaids consisting of two superimposed component gratings. These data sets were previously analyzed after averaging across trials of the same stimulus (Busse et al., 2009); here, we examined them in individual trials.

### Variability Is Shared across the Population

Repeated presentations of the same stimulus elicited highly variable responses, yet this variability was coordinated across the population. To illustrate the nature of this variability, we plotted “population tuning curves”, a graphical summary of the population response to a plaid stimulus (Figure 1B). As expected, a plaid with very different component contrasts evoked the largest mean activity at sites tuned for the orientation of the high-contrast component grating (Figure 1C), a form of winner-take-all competition (Busse et al., 2009). Yet, the responses of all tuned sites varied from trial to trial; for instance, firing rate tended to be higher on one trial than on another, and this difference affected most sites simultaneously (Figure 1D). As a result, the curves fitted to the population activity changed noticeably from trial to trial (Figure 1E). Similar results were obtained for different stimuli. For instance, as observed previously (Busse et al., 2009), a plaid with equal component contrasts elicited large mean responses at sites tuned for either of the component orientations (Figure 1F). Trial-by-trial responses were again highly variable, and the variability seemed to be coordinated at the population level (Figures 1G and 1H).

Although trial-by-trial variability was clearly coordinated across the population, the nature of this shared variability was not immediately obvious. In the first example, population responses seemed to be scaled multiplicatively between trials (Figure 1D). Yet, in the second example, population responses seemed to be shifted by a common offset between trials (Figure 1G). There were also examples that spoke in favor of a mixture of additive and multiplicative effects (Figures 1E and 1H).

To explore the structure of this variability and to gain an intuition into how it is shared between neurons, we characterized the population response on each trial as a single, compact population tuning curve determined by three free parameters. The population tuning curve $R_{i}$ (a vector whose values are the normalized firing rates of all orientation-tuned sites, ordered by their preferred orientations) on trial *i* was fit as a linear combination of prototypical responses to the two component gratings of a plaid, plus a constant shift (Figure 1B):

(Equation 1) $$R_{i}=\alpha_{1}^{i}G\left(\theta_{1}^{i}\right)+\alpha_{2}^{i}G\left(\theta_{2}^{i}\right)+\beta^{i}.$$

Here, $\theta_{1}^{i}$ and $\theta_{2}^{i}$ are the component orientations of the plaid stimulus presented on trial *i*, and G(θ) is the prototypical response (a circular Gaussian) to a grating of orientation θ. The population tuning curve ***R***_i_ on each trial *i* is thus determined by three parameters: the tuned response to component grating 1, $\alpha_{1}^{i}$, the tuned response to component grating 2, $\alpha_{2}^{i}$, and a baseline untuned response, $\beta^{i}$. These parameters were fit by least-squares to the population response; they effectively summarized the population activity of many tens of sites on each trial, accounting for 47 ± 18% (median ± median absolute deviation) of the variance. We used these fits to examine the population data and the predictions of various models of neuronal variability (but not to fit the models, which was done on the actual spike counts as a function of site and time, as described below).

The parameters of the population tuning curves confirmed that the trial-to-trial variability included a shared multiplicative component (Figure 1I). For a plaid with equal component contrasts, the tuned-response components to gratings 1 and 2, $\alpha_{1}^{i}$ and $\alpha_{2}^{i}$, were positively correlated across trials: the ellipse summarizing their distribution was clearly diagonal (Figure 1I, *black; r = 0.45*). By comparison, for a plaid with markedly different component contrasts, the tuned response $\alpha_{2}^{i}$ to the low-contrast grating stayed close to zero regardless of the tuned response $\alpha_{1}^{i}$ to the high-contrast grating (Figure 1I, *blue*; *r =* −*0.05*). These two elongated clouds radiating outward from the origin are what would be expected from shared multiplicative variability. They do not, however, rule out the presence of additive variability, which causes variations in the parameter *β^i^* (Figure S3).

### The Affine Model

**Figure 2:**
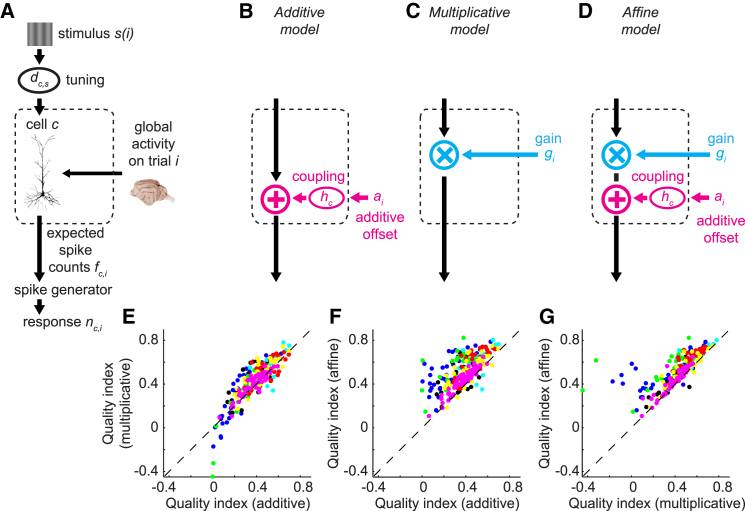
The Additive, Multiplicative, and Affine Models (A) The basic structure of the models involves a unit *c*, whose expected spike count *f*_*c,i*_ on trial *i* depends on both the tuning *d*_*c,s*_ of that unit for stimulus *s*(*i*) shown on that trial and global, shared factors that originate from the rest of the brain and vary from trial to trial. The expected spike count *f*_*c,i*_ is then passed into a stochastic spike count generator that generates private variability, yielding an integer spike count *n*_*c,i*_ from a negative binomial distribution with mean *f*_*c,i*_ and cell- and stimulus-dependent Fano factor *F*_*c,s*_. (B) In the additive model, the global factor is the additive offset *a*_*i*_ that affects each unit *c* by an amount proportional to a coupling term *h*_*c*_. (C) In the multiplicative model, the global factor is the variable response gain *g*_*i*_ that uniformly scales the sensory drive *d*_*c,s*_. (D) The affine model includes both the additive and multiplicative components. (E–G) Cross-validated performance of the response *n*_*c,i*_ generated by the multiplicative model versus the additive model (E) and by the affine model versus the additive (F) and multiplicative (G) models across seven sessions in three cats. The performance was measured by the quality index, which is zero or negative if the prediction is not better than the independent model and equals 1 for a perfect prediction. Each circle represents the performance on one site across all trials in a session; sites from the same session share the same color. Only sites that had quality index > 0.1 for at least one of the models were shown.

The shared nature of trial-to-trial variability described above suggests that trial-to-trial fluctuations in population activity might be accountable by a small number of factors, whose joint effect on each neuron can be multiplicative and/or additive. To formalize this idea, we turned to a more rigorous approach that seeks to predict the response of every site to every stimulus on every trial. We developed a set of models that operate at the level of spike trains of units (single neuron or multiunit) in a population (not at the level of the summary statistics α_1_, *α*_2_, and *β* previously described). In these models, the response of each unit on a trial depends on both the tuning of that unit to the stimulus shown on that trial and a trial-varying, global, shared factor that originates from the rest of the brain (Figure 2A). To account for the different factors of shared variability, we considered an additive model (Figure 2B), a multiplicative model (Figure 2C), and an affine model (Figure 2D), which encompasses them both.

In the affine model, the expected spike count of unit *c* on trial *i*, during which stimulus *s*(*i*) is presented, is

(Equation 2) $$f_{c,i}=g_{i}d_{c,s\left(i\right)}+a_{i}h_{c},$$

where $d_{c,s\left(i\right)}$ is the deterministic sensory drive to unit *c* arising from stimulus *s*(*i*). This term reflects the unit’s sensory tuning as well as its contextual interactions; i.e., anything that contributes to the unit’s mean response to that stimulus; e.g., divisive normalization (Busse et al., 2009). The multiplicative gain *g*_*i*_ scales the firing rate of all units in proportion to their sensory drive; i.e., it controls their response gain. The additive offset *a*_*i*_ adds to their firing rates in proportion to their coupling factors *h*_*c*_. Constraining *g*_*i*_ = 1 gives a purely additive model (Figure 2B); setting *a*_*i*_ = 0 results in a purely multiplicative model (Figure 2C). Constraining both *g*_*i*_ = 1 and *a*_*i*_ = 0 gives a model that takes no shared variability into account (referred as the independent model; Figure S4A).

To compare model predictions with recorded spike counts, we passed *f*_*c,i*_ into a stochastic spike count generator, which yields an integer spike count *n*_*c,i*_ from a negative binomial distribution with mean *f*_*c,i*_ and cell- and stimulus-dependent Fano factor *F*_*c,s(i)*_, estimated by maximum likelihood. This stochastic spike count generator delivers the fraction of variability that is not shared, but private to each unit (Deweese and Zador, 2004).

### Population Variability Is Both Multiplicative and Additive

To study how well these global, shared factors could explain cortical variability, we fit the affine model and compared the results with the purely additive and multiplicative models. We obtained the model parameters by fitting the model predictions *f*_*c,i*_ to the spike counts recorded at each site, and we evaluated the fits with cross-validation (see Supplemental Information: cross-validation; Figure S1). Performance was measured by the quality index *q*: the improvement in cross-validated prediction compared to the independent model with no shared variability. The quality index is zero or negative for a model that offers no improvement over the independent model, and equals 1 for a perfect prediction.

This analysis showed that the affine model is superior to both the additive and multiplicative models (Figures 2E–2G). The multiplicative model performed better than the additive model (Figure 2E; Table S1; p < 10^−11^ for all data together, p < 0.03 in five out of seven recording sessions evaluated individually; sign test). In all sessions, the affine model considerably outperformed both the additive model (Figure 2F; Table S1; p < 10^−43^ for all data together, p < 10^−6^ in five individual sessions, and p < 0.02 in the remaining two; sign test) and the multiplicative model (Figure 2G; Table S1; p < 10^−43^ for all data together, p < 10^−8^ in four individual sessions, and p < 0.001 for the rest; sign test). Because these results were cross-validated, the affine model could not gain a numerical advantage by over-fitting. Rather, the results indicate that neither the additive nor multiplicative factors alone suffice: combining the two forms a better model for shared cortical variability.

Note that the additive model—unlike the multiplicative model—has a coupling term *h*_*c*_ that allows each unit to be coupled to population activity differently, a phenomenon that has been previously described (Okun et al., 2015). Introducing a similar cell-coupling term to the multiplicative model did improve its performance, but this extended multiplicative model still performed worse than the affine model (Figures S2A–S2D; Table S2; Supplemental Information: the extended multiplicative model). The superior performance of the affine model thus reflects the presence of an additive component rather than simply its ability to model different coupling strengths of different neurons.

**Figure 3:**
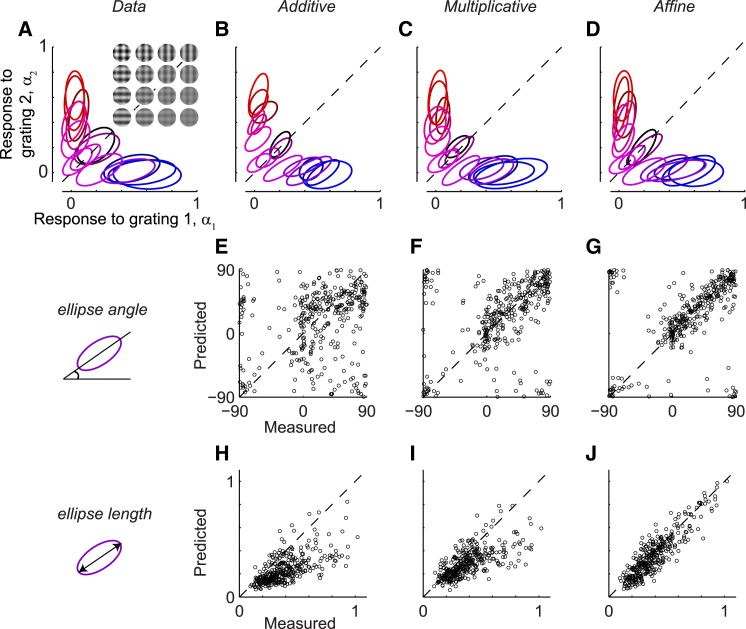
Variability of Population Response to Plaid Stimuli (A) Ellipses showing 1 SD contours of Gaussian fits to the distributions of population responses to gratings 1 and 2 (*α*_1_ and *α*_2_) for an example session. The 16 ellipses correspond to 16 plaid stimuli in which the contrasts of the two component gratings are varied independently. The colors indicate the component contrasts (RGB color code with red encoding the contrast of grating 1 and blue encoding the contrast of grating 2). For each component-contrast combination, the data were pooled across different component orientations. Two of the ellipses appeared in Figure 1I (session 83-7-5, 45 orientation-tuned sites, plaid angle = 90°). (B–D) Ellipses fitted to responses simulated by the additive (B), multiplicative (C), and affine (D) models. (E–G) Comparison of the ellipse angles generated by the three models versus experimental data across seven sessions in three cats. Each plaid stimulus in each session contributes a dot for the ellipse fitted to the distribution of *α*_1_ versus *α*_2_. (H–J) Same as (E)–(G), but for the comparison of the major-axis lengths of the ellipses.

To gain further intuition into the performance of the models, we returned to the reduced representation of the population activity (Figure 3). We first computed the population tuning curves for the recorded and simulated activities. We then fit ellipses to summarize their variability (as in Figure 1I) and examined the ellipses corresponding to all plaid stimuli (Figures 3, S3, and S4). In the data, the tuned responses to component gratings 1 and 2 tended to be correlated only for plaids with equal component contrasts, resulting in ellipses emanating from the origin (Figure 3A). The additive model produced tuned responses that were slanted (i.e., highly correlated) for all plaid stimuli, including those with unequal component contrasts (Figure 3B). The multiplicative and affine models, by contrast, captured the angle of the ellipses appropriately (Figures 3C and 3D). Indeed, the affine model outperformed both the additive and multiplicative models in predicting the ellipse angles across all seven sessions (Figures 3E–3G). The affine model also provided better predictions of ellipse lengths, which reflect the strength of correlated variability (Figures 3H–3J). Analyzing correlations between the tuned responses and the baseline, untuned activity gave similar conclusion (Figure S3). Finally, all three models outperformed the independent model (Figure S4).

In short, the affine model was superior in capturing correlations between the two tuned-response components as well as correlations between the tuned- and untuned-response components. The affine model thus explains much of the response variability and surpasses both the additive and multiplicative models in predicting not only the raw individual spike counts (Figure 2), but also our summary analysis of ensemble activity, the population tuning curves (Figures 3, S3, and S4).

### Dependence of Noise Correlations on Tuning Similarity

Our results suggest that the correlated response variability of neuronal populations can be well modeled by only two global factors, additive and multiplicative, that are shared across the population. In a population of *N* neurons, there are order *N*^*2*^ pairwise correlations. To what extent can these pairwise correlations be explained by these two factors?

To address this, we measured pairwise noise correlations for all pairs of orientation-tuned sites in response to single-grating stimuli. Correlations tended to be high because they were measured over long time windows and involved multiunit activity (Cohen and Kohn, 2011). We found that noise correlations predicted by the affine model were closer to the measured values, compared to either the additive model (Figures S5A–S5U; p = 10^−48^ for all data together, p < 10^−4^ in four individual sessions, and p < 0.05 in one session; sign test on the squared errors between measured and predicted noise correlations) or the multiplicative model (Figures S5A–S5U; p < 10^−54^ for all data together, p < 10^−5^ in five individual sessions, and p < 0.05 in the remaining two; sign test).

We next asked whether the affine model could predict the well-known relationship between signal and noise correlations: noise correlations are larger between cells with similar sensory tuning (reviewed in Cohen and Kohn, 2011). This relationship is often attributed to increased connectivity between neurons with similar sensory tuning, but it can also arise from shared multiplicative variability (Brody, 1999; Ecker et al., 2014; Goris et al., 2014). This is simply because multiplication affects neurons that are responding to a stimulus more than neurons that are not responding, thus introducing correlations among neurons with similar tuning.

**Figure 4:**
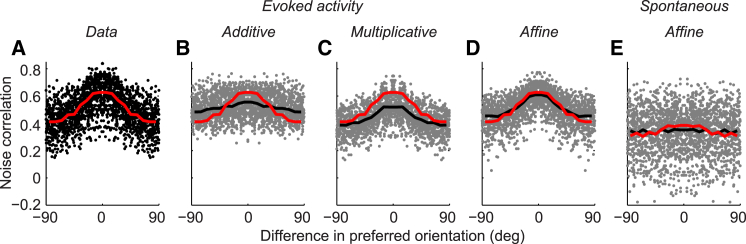
Relationship between Noise Correlation and Tuning Similarity (A) Noise correlation for each pair of orientation-tuned sites in response to single gratings, as a function of the difference between their preferred orientations. A black dot represents noise correlation calculated for a pair of sites, and the red curve shows the running median. (B–D) As in (A), but for predictions of the additive (B), multiplicative (C), and affine (D) models. A gray dot represents noise correlation predicted by the model for a pair of sites, a black curve shows the running median, and a red curve repeats the running median of the measured data for comparison. (E) As in (D), but for spontaneous correlations. Note that even for spontaneous activity, noise correlations were higher for similarly tuned sites, but this could not be captured by the model. All running medians were calculated with non-overlapping 10° bins (session 83-7-5, 45 orientation-tuned sites).

We measured the dependence of noise correlations on the difference in preferred orientations and compared it to the one calculated from spike counts simulated by the additive, multiplicative, and affine models (Figure 4). As expected, noise correlations showed a clear dependence on orientation-tuning similarity: sites preferring the same orientation were more strongly correlated (Figure 4A). This dependence could not be captured by the additive model, which predicted a negligible dependence on tuning similarity (Figure 4B). The multiplicative model predicted stronger noise correlations when preferred orientations were more similar, but correlations were generally underestimated (Figure 4C). The affine model almost completely accounted for the dependence of noise correlations on tuning similarity (Figure 4D); this superiority was statistically significant in all sessions (p < 0.05 for both additive versus affine and multiplicative versus affine; *t* test on each session’s sum of squared errors between running medians of data and model; Figure S6).

Still, the affine model slightly underestimated the dependence of pairwise correlations on tuning difference (Figure 4D). To investigate this mild imperfection, we looked at the spontaneous correlations; i.e., the pairwise correlations measured in the absence of stimuli (Figure 4E). Spontaneous correlations have been reported to be strongest for similarly tuned cells (Jermakowicz et al., 2009; Kenet et al., 2003; Okun et al., 2015). Our data showed this effect, albeit weakly (Figure 4E, red curve). Unsurprisingly, none of the three models could predict this dependence; for instance, the predictions of the affine model were essentially flat (Figure 4E, black curve). This small, but noticeable, error in accounting for spontaneous correlations hints at what might be missing from the affine model: a slightly higher coupling between neurons that are similarly tuned.

**Figure 5:**
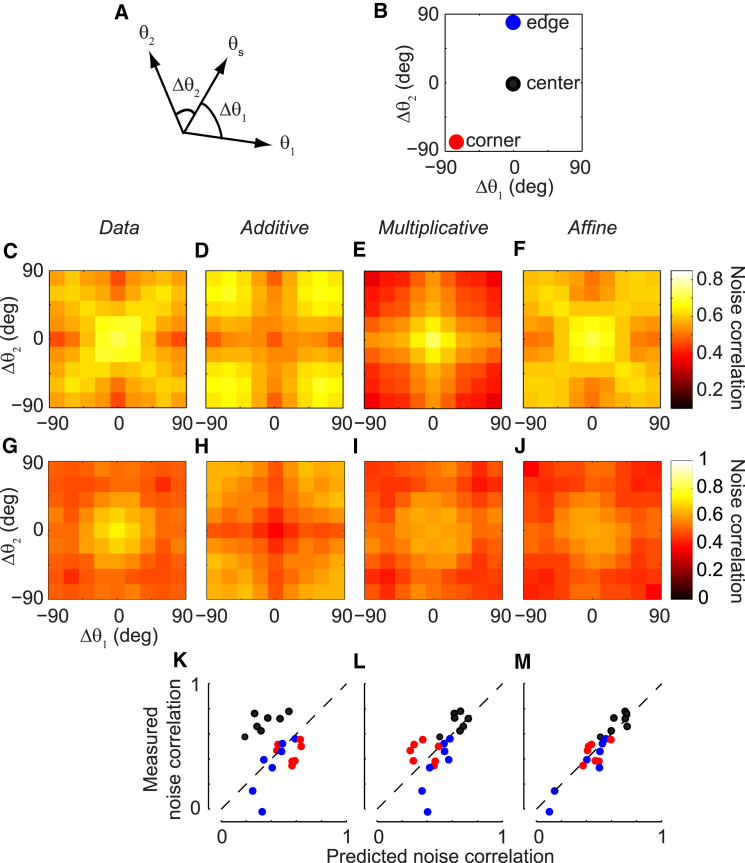
Dependence of Noise Correlations on Stimulus Orientation and Tuning Preferences (A) Noise correlation for each pair of sites in response to a single grating was analyzed as a function of three parameters: the stimulus orientation, *θ*_*s*_; the preferred orientation of site 1, *θ*_1_; and the preferred orientation of site 2, *θ*_2_. Assuming rotational symmetry, we could reduce these three parameters to two: the difference between the preferred orientations of the two sites, and the stimulus orientation, $\Delta \theta_{c}=\theta_{c}-\theta_{s}$, for *c* = 1 or 2. (B) The dependence of noise correlations on stimulus orientation and tuning preferences are summarized by three numbers: the median noise correlations in the center bin (black circle, measuring the correlation produced when two co-tuned sites are stimulated with a grating of their preferred orientation), the corner bin (red circle, measuring the correlation produced when two co-tuned sites are stimulated with a grating whose orientation is orthogonal to their preferred orientation), and the edge bin (blue circle, measuring the correlation produced when two oppositely tuned sites are stimulated with a grating of one of their preferred orientations). (C) Pseudocolor representation of median noise correlations for all pairs of orientation-tuned sites as a function of $\Delta \theta_{1}$ and $\Delta \theta_{2}$. The data were pooled across all contrasts and orientations (session 83-7-5, 45 orientation-tuned sites). (D–F) As in (C), but for predictions of the additive (D), multiplicative (E), and affine (F) models. (G–J) Same as (C)–(F), but for a different session in which the multiplicative model alone could reasonably reproduce the structure of the measured correlation matrix (session 83-10-15, 42 orientation-tuned sites). (K–M) Scatter plots comparing the measured and predicted correlations for the additive (K), multiplicative (L), and affine (M) models across seven sessions in three cats. Each circle shows the measured and predicted noise correlations for one bin and one session, color-coded as in (B).

To further understand the predictions of the three models, we considered an additional factor that determines noise correlations: the orientation $\theta_{s}$ of the stimulus relative to the preferred orientations $\theta_{1}$ and $\theta_{2}$ of the two sites. Noise correlations between a neuronal pair depend not only on the neurons’ sensory tuning, but also on the attributes (orientation in this case) of the stimulus (Kohn and Smith, 2005; Ponce-Alvarez et al., 2013). Assuming rotational symmetry, this dependence on stimulus orientation and cell tuning can be summarized by a function $\rho \left(\Delta \theta_{1},\Delta \theta_{2}\right)$, where $\Delta \theta_{c}=\theta_{c}-\theta_{s}$ is the preferred orientation of site *c* relative to stimulus orientation $\theta_{s}$ (Figure 5A). This representation was particularly informative in three locations: (1) the “center” bin, where the preferred orientations of both sites match the stimulus orientation; (2) the “edge” bin, where one site’s tuning matches the stimulus and the other is orthogonal to it; and (3) the “corner” bin, where the preferred orientations of both sites are orthogonal to the stimulus orientation (Figure 5B).

This representation revealed a rich structure of pairwise correlations (Figures 5C–5J). In a typical session, noise correlations peaked in the center bin (Figure 5C); this observation was well predicted by the multiplicative and affine models (Figures 5E and 5F), but not by the additive model (Figure 5D). As has been previously observed (Cotton et al., 2013), correlations between similarly tuned sites were also high in the corner bins; this was predicted by the additive and affine models (Figures 5D and 5F), but not by the multiplicative model (Figure 5E). Finally, the data showed the lowest correlations in the edge bins; this was predicted by the affine and additive models (Figures 5D and 5F), but not by the multiplicative model (Figure 5E). While this particular structure of the correlation matrix was the most common (four out of seven sessions), it was not the only one we observed. Other recordings showed a different correlation structure that, again, was well captured by the affine model (e.g., Figures 5G–5J).

To assess each model’s performance across sessions, we computed the median noise correlations that fell into the center, corner, and edge bins of the correlation matrix (Figures 5K–5M). As in previous examples, the additive model tended to underestimate correlations in the center bin (Figure 5K). The multiplicative model did better (Figure 5L), but not as well as the affine model (Figure 5M). Note that the correlation matrices predicted by the extended multiplicative model were similar to the multiplicative model (Figures S2H–S2O). Only the affine model could predict all possible structures of the correlation matrices. In fact, the rich structure of these correlation matrices can be analytically predicted by the affine model (see Supplemental Information: analytic calculation of pairwise correlations; Figure S7). We conclude that the affine model concisely accounts for the complex, session-dependent relationship of noise correlations to neuronal tuning and stimulus.

### Shared Cortical Variability in Quietly Awake Mice Is Both Multiplicative and Additive

We next asked whether the affine model—derived from anesthetized cat data—is also a good description of shared variability in the unanesthetized cortex. To this end, we used multisite silicon probes to record population activity in V1 of quietly awake, head-fixed mice (five sessions in five neuronal populations in four mice) in response to drifting gratings (12 directions at 100% or 60% contrast). The data were spike-sorted and only stable, well-isolated single units were used for further analysis. As expected (Busse et al., 2009; Niell and Stryker, 2008), the mean firing rates were substantially lower for single neurons in mouse V1 than for multiunit activity in cat V1 (mean of 2.8 versus 30.3 spikes/s). Even with such low firing rates, a majority (58%) of neurons showed detectable shared variability (quality index *q* > 0.1 for at least one of the models; Table S3). We focused on these neurons, fit the models, and asked which model better described this shared variability.

**Figure 6:**
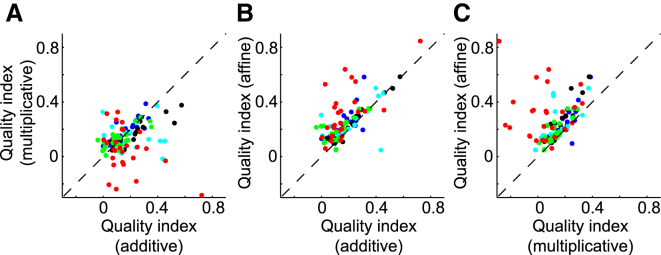
Performance of the Additive, Multiplicative, and Affine Models in Single-Unit Data from Quietly Awake Mice (A–C) Cross-validated performance of the multiplicative model versus the additive model (A) and of the affine model versus the additive (B) and multiplicative (C) models (cf. Figures 2E–2G). Each circle represents the performance on one neuron across all trials in a session; neurons from the same session share the same color (five recording sessions in four mice). Only neurons that had a quality index > 0.1 for at least one of the models were shown.

The results demonstrate that as in anesthetized cats, shared cortical variability in awake mice could be described by a combination of additive and multiplicative components (Figure 6). Differences between the multiplicative and additive models were small (Figure 6A; Table S3; p = 0.30 for all data together and p > 0.05 in all individual sessions; sign test). The affine model, on the other hand, performed significantly better than either the additive model (Figure 6B; Table S3; p < 10^−12^ for all data together and p < 0.008 in all individual sessions; sign test) or the multiplicative model (Figure 6C; Table S3; p < 10^−16^ for all data together and p < 0.002 in all individual sessions; sign test). Again, the extended multiplicative model did not fare as well as the affine model (Figures S2E–S2G; Table S4).

The affine model also outperformed the additive and multiplicative models in predicting noise correlations in single-unit data from quietly awake mice. As expected on the basis of lower firing rates (de la Rocha et al., 2007; Dorn and Ringach, 2003), measured correlations were considerably lower than in the multiunit data from anesthetized cats (noise-correlation means of 0.09 versus 0.38). Nonetheless, they could still be used to distinguish the performance of the models. As with the cat data, the affine model trumped both the additive model (Figures S5, p < 10^−4^ for all data together, p < 0.005 in two individual sessions, and p < 0.04 in two others; sign test on the squared errors between measured and predicted noise correlations) and the multiplicative model (Figures S5, p < 10^−31^ for all data together and p < 0.001 in all individual sessions; sign test).

### Effect of Multiplicative Gain and Additive Offset on Population Coding

We have shown that cortical population variability can be well described by two sources of shared fluctuation: multiplicative and additive. How do these two sources of fluctuation impact information coding? To address this question, we used the responses of a neuronal population governed by the affine model to distinguish stimuli that differ subtly in orientation or contrast. To quantify this aspect of population decoding, we used a linear discriminability measure (Averbeck and Lee, 2006; Poor, 1994):

(Equation 3) $$d^{2}={\left({\bar{n}}_{2}-{\bar{n}}_{1}\right)}^{T}{\text{Σ}}^{-1}\left({\bar{n}}_{2}-{\bar{n}}_{1}\right).$$

Here ${\bar{n}}_{1}$ and ${\bar{n}}_{2}$ are the trial-averaged population response vectors to stimuli 1 and 2, and Σ is the population covariance matrix, derived from all presentations of the two stimuli. Since ${\bar{n}}_{1}$, ${\bar{n}}_{2}$, and Σ can be analytically calculated under the affine model (Supplemental Information: analytic calculation of pairwise correlations), we can estimate the dependence of *d*^2^ on model parameters. For comparison, we also evaluated this measure for a population of independent, uncorrelated neurons having the equivalent amount of variability. To obtain a discriminability measure $d_{\text{shuffled}}^{2}$ based on those uncorrelated responses, we set all off-diagonal values of the covariance matrix Σ (corresponding to correlations between neurons) to zero. Depending on the structure of the correlation, its value can be superior or inferior to *d*^2^ (Abbott and Dayan, 1999; Averbeck et al., 2006; Moreno-Bote et al., 2014).

We first considered the effect of changing the mean of either the additive offset or the multiplicative gain. Increasing the mean of the multiplicative gain enhanced coding, whereas increasing the mean additive offset slightly degraded it (data not shown). This accords with classic studies of tuning curves: the steeper the tuning curve and the lower the baseline, the larger the decoding accuracy (Dayan and Abbott, 2001).

**Figure 7:**
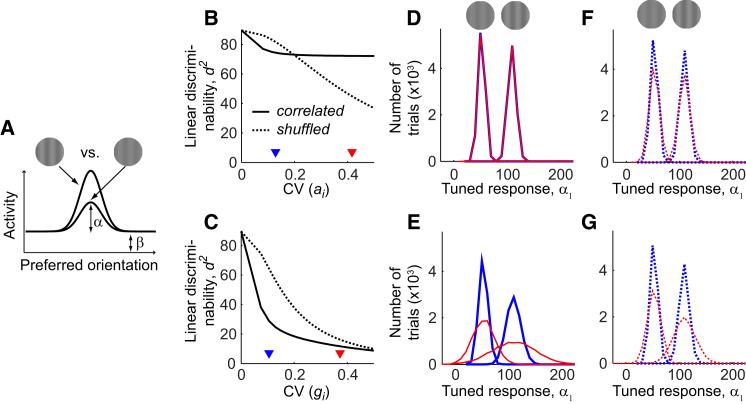
Effects of Fluctuations in Multiplicative Gain and Additive Offset on Contrast Coding (A) Cartoon depicting the contrast discrimination task. The simulated activity of a homogenous neuronal population was used to discriminate two gratings of the same orientation at 6% and 12% contrasts; curves indicate population tuning curves in response to the two stimuli to be discerned. The population tuning curve on each trial is fitted as a linear combination of a unit Gaussian and a constant offset. (B and C) Discriminability measure *d*^2^ (solid) and the corresponding $d_{\text{shuffled}}^{2}$ (dashed) between population responses in the discrimination task as a function of the coefficients of variation (CV) of the additive offset (B) and the multiplicative gain (C). (D) Distributions of tuned-response amplitudes *α* from simulated population activity on 10^4^ trials of a discrimination task for a low value (blue) and a high value (red) of CV(*a*_*i*_). The two values used are marked by triangles of the same color in (B). (E) As in (D), but for two different values of CV(*g*_*i*_), the variability of shared multiplicative fluctuations. The two values are marked by triangles of the same color in (C). (F and G) As in (D) and (E), but for their uncorrelated counterparts.

**Figure 8:**
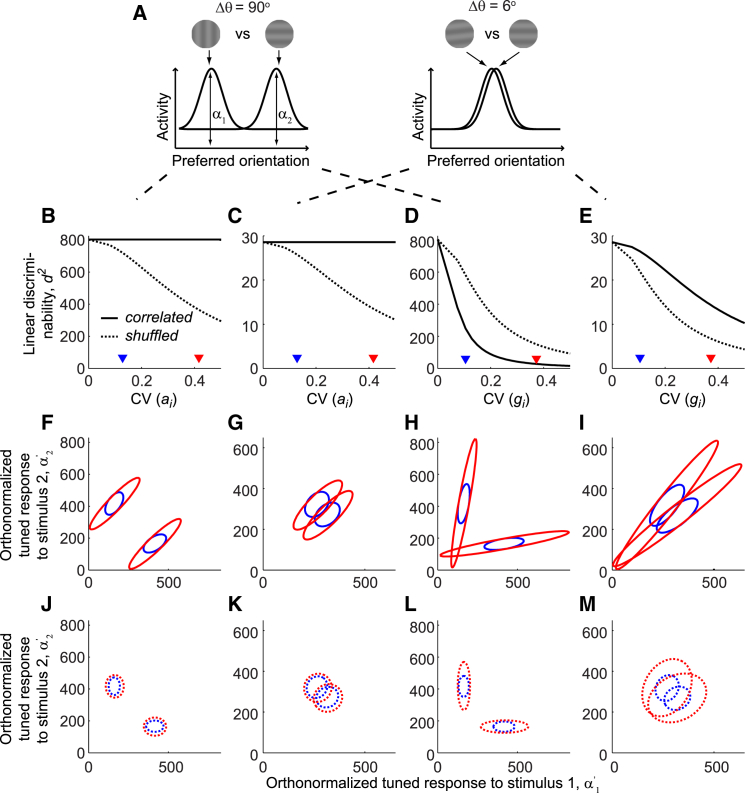
Effects of Fluctuations in Multiplicative Gain and Additive Offset on Orientation Coding (A) Cartoon showing two orientation discrimination tasks. The activity of a homogenous neuronal population was used to distinguish two 12%-contrast gratings whose orientations differed by 90° and 6°. The population tuning curve on each trial was fitted as a linear combination of two unit Gaussians centered on the orientations of stimuli 1 and 2. *α*_1_ and *α*_2_ thus summarized the tuned amplitudes of the population activity to stimuli 1 and 2, respectively. (B–E) Discriminability measure *d*^2^ (solid line) and the corresponding $d_{\text{shuffled}}^{2}$ (dashed line) between population responses in the two tasks as a function of the coefficients of variation (CV) of the additive offset (B and C) and the multiplicative gain (D and E). The results from discriminating an orientation difference of 90° were plotted in (B) and (D); and results from fine discrimination (6°) were plotted in (C) and (E). (F–I) Ellipses showing 3 SD contours of Gaussian fits to the distributions of population tuned responses to stimuli 1 and 2 on 10^4^ trials of an orientation discrimination task at two different values of CV(*a*_*i*_) (F and G) and CV(*g*_*i*_) (H and I). For simple visualization, these plots show orthonormalized responses $\alpha_{1}^{\prime }$ and $\alpha_{2}^{\prime }$. The two values of CV(*a*_*i*_) and CV(*g*_*i*_) were marked by triangles in (B)–(E) with the same color coding. (J–M) As in (F)–(I), but for trial-shuffled responses in which the equivalent variability occurs across uncorrelated neurons.

We next considered the effect of trial-to-trial fluctuations in the additive and multiplicative terms. We simulated a contrast discrimination task (Figure 7A) and an orientation discrimination task (Figure 8A). The contrast discrimination task involved distinguishing two gratings of the same orientation, but different contrasts (6% and 12%); the orientation discrimination task involved distinguishing two 12%-contrast gratings whose orientations differed by either 6° or 90°.

In the contrast discrimination task, shared multiplicative fluctuations had a much larger effect on stimulus coding than additive ones. Increasing the variability of the additive offset hardly had any impact on discriminability, triggering a small decline that became noticeable only after trial-shuffling, when variability was uncorrelated across neurons (Figure 7B). On the other hand, increasing the variability of the multiplicative gain sharply decreased discriminability; e.g., discriminability dropped by a factor of 5 when the coefficient of variation of *g*_*i*_ increased from 0 to 0.5. This decline was reduced in $d_{\text{shuffled}}^{2}$, indicating that it is specific to multiplicative variability that is shared across neurons (Figure 7C). These trends also held for tasks involving different pairs of contrasts (Figures S8B–S8D).

To understand why contrast discrimination would particularly suffer from shared multiplicative fluctuations, we fit the simulated population response on a single trial as a linear combination of a unit circular Gaussian and a constant offset (Figure 7A). Population activity on each trial was thus summarized by two parameters: the tuned amplitude α and the baseline amplitude *β*. Discriminability could then be understood from the overlap of the distributions of the tuned responses *α* to the two stimuli: the more they overlap, the lower is discriminability. Increasing the variability of the additive offset hardly altered the shapes of these distributions, explaining the negligible effect of additive fluctuations on coding (Figure 7D). Rather, additive fluctuations affected the baseline amplitude *β*, which cannot be used to discriminate the two stimuli (data not shown). By contrast, increasing the variability of the multiplicative gain broadened the distributions of the tuned amplitudes *α*, making them much harder to discern (Figure 7E).

We obtained further insight into the effect of correlations induced by shared additive and multiplicative fluctuations by comparing them to the effect of equivalent uncorrelated variability using a shuffling analysis (Figures 7F and 7G). Comparing the *α* distributions calculated from the additive-correlated responses to stimuli 1 and 2 (Figure 7D) to their shuffled counterparts (Figure 7F) showed that additive-correlated variability was less detrimental than its shuffled equivalent (see also Figure S8M). By contrast, the *α* distributions obtained with shared multiplicative fluctuations (Figure 7E) had a greater overlap than the ones from the corresponding shuffled trials (Figure 7G). This indicates that in the contrast discrimination task, additive-correlated variability had a minor effect, but multiplicative-correlated variability worsened discriminability.

The effect of shared fluctuations on the orientation discrimination task depended on the type of fluctuations and the difficulty of the task (Figure 8). Shared additive fluctuations had no effect on discriminability, which deteriorated only after trial-shuffling, when variability was no longer shared among neurons (Figures 8B and 8C). Shared multiplicative fluctuations, on the other hand, had large consequences that depended on the difficulty of the task (Figures 8D and 8E). For most orientation differences, they had a strong detrimental effect, which was reduced by trial-shuffling (Figures 8D, S8G, S8H, S8K, and S8L). Yet, when the task involved discriminating stimuli of fine orientation difference, performance was actually worsened by trial-shuffling (Figures 8E, S8E, and S8I), indicating that shared multiplicative fluctuations allow better fine-orientation discrimination than the equivalent uncorrelated variability.

To understand these effects, we fit population responses as a sum of responses to the two gratings being discriminated (Figure 8A) and plotted ellipses illustrating the means and covariances of the population responses (Figures 8F–8M; see also Supplemental Information: decoding analysis with reduced dimensionality). For two gratings of very different orientations, the mean responses were far apart; shared multiplicative fluctuations elongated the two ellipses in such a way that they started to overlap, which reduced their discriminability (Figure 8H). Introducing the same amount of variability to uncorrelated neurons by trial-shuffling did not increase the overlap significantly (Figure 8L). Conversely, for fine-orientation discrimination, the mean responses to the two stimuli were very close (Figure 8I). Shared multiplicative fluctuations again elongated the two ellipses, but because the direction of this elongation was almost orthogonal to the separation between the centers, its effects on overlap were relatively minor. When the equivalent variability was applied independently to the neurons via trial-shuffling, the elongated ellipses were replaced by nearly circular shapes that showed greater overlap (Figure 8M). We observed similar trends in orientation discrimination tasks that involved gratings of a different contrast (Figures S8I–S8L).

In short, shared additive fluctuations are not detrimental to either contrast or orientation discrimination. Shared multiplicative fluctuations, on the other hand, are detrimental to the discrimination of contrast and of coarse orientation differences, but are less harmful to fine-orientation discrimination than an equivalent amount of uncorrelated variability.

## Discussion

We analyzed the activity of large neuronal populations in V1. We found that much of the trial-by-trial variability is shared across the population and can be summarized using only two global factors: additive and multiplicative. These results may reconcile the long-held view that variability is additive (Arieli et al., 1996; Schölvinck et al., 2015) with subsequent claims of it being predominantly multiplicative (Ecker et al., 2014; Goris et al., 2014). The former view hypothesizes that the large response variability on individual trials comes from adding ongoing cortical activity onto a deterministic sensory response. The latter postulates that much of the response variability arises from fluctuations in excitability. We argue that it is not one or the other, but a combination of the two.

Our data suggest that common multiplicative gain fluctuations play a dominant role in the structure of pairwise noise correlations. There are two long-standing hypotheses for the circuit mechanisms underlying the similarity of signal and noise correlations. The first is that correlations arise from synaptic connectivity patterns. In visual cortex, neurons with similar orientation preferences are preferentially connected, thus co-tuned neurons will also share a larger fraction of common input from cells of similar sensory preference (Ko et al., 2011), increasing their correlations. The second is that correlations reflect common modulation by multiplicative gain fluctuations (Brody, 1999). Indeed, even a pair of neurons that are unconnected and share no inputs would exhibit noise correlations if they experienced common multiplicative modulation, and the strength of these correlations would be stronger for neurons with similar sensory tuning. Our data provide evidence in favor of both hypotheses, but ascribe a larger role to the mechanism of shared multiplicative fluctuations. Indeed, the affine model generated a good fit to the dependence of noise correlation on orientation tuning (Figures 4 and S6) and only a small residual was left to be explained. Intriguingly, this residual resembled the small, but significant, dependence of spontaneous correlations on tuning difference. The affine model could not possibly capture this dependence, because it has no way to preferentially assign shared factors as a function of tuning similarity. The similarity of spontaneous and signal correlations, matching the residual error in the affine model’s prediction, thus suggests that specific synaptic connectivity does contribute to producing noise correlations, although this contribution is numerically smaller than the contribution of common gain fluctuations.

Our results lend weight to the multiplicative model, but also reveal its possible limitations. For example, shared multiplicative fluctuations alone were insufficient to predict the complex relationship between correlations, the sensory tuning of both units, and the stimulus (Figure 5). The affine model—by adding an additional common additive offset on each trial—resolved this limitation.

Nevertheless, there are certain features that may further improve the affine model. For instance, Okun et al. (2015) show that different neurons are coupled to global fluctuations differently. In the current affine model, only the additive component has a cell-coupling term that allows each neuron to be coupled to population activity to different degrees. We saw that it is possible to extend the multiplicative model in such a way; this extension improved the fit of the multiplicative model, but not to the extent that it matched the affine model (Figure S2). Future work could explore the possibility of combining the extended multiplicative model and the additive model; however, this would require substantially greater amounts of single-unit data than analyzed here.

What candidate circuit mechanisms might underlie the multiplicative and additive fluctuations described here? A possibility lies in different interneuron classes: activation and inactivation of parvalbumin- or somatostatin-positive interneurons have been variously suggested to have multiplicative, additive, and combined (affine) effects on the firing rates of pyramidal cells (Atallah et al., 2012; Lee et al., 2012; Wilson et al., 2012). Another possibility lies in top-down connections from higher order cortices and thalamus. These inputs, which target distal apical dendrites in layer 1, can rarely elicit spikes in pyramidal cells, but may boost the gain of pyramidal cells’ responses to more proximal sensory inputs (Larkum, 2013; Larkum et al., 1999, 2004). Such top-down connections are believed to play a role in attentional modulation of V4 firing (Armstrong and Moore, 2007; Moore and Armstrong, 2003), which has been reported to have both a multiplicative and an additive effect on visual responses (Boynton, 2009; Buracas and Boynton, 2007; Murray, 2008; Reynolds and Heeger, 2009; Thiele et al., 2009; Williford and Maunsell, 2006). In addition, multiplicative effects have been observed in other contexts such as normalization (Busse et al., 2009; Carandini and Heeger, 2012). The multiplicative and additive fluctuations might also share circuits with those that modulate responses based on locomotion, which has both an additive and a divisive effect (Ayaz et al., 2013).

Additive and multiplicative fluctuations have very different consequences for the cortical coding of sensory information. An analytically tractable model based on our experimental results revealed that for both contrast and orientation discriminations, additive fluctuations had negligible effect on the discriminability of sensory stimuli, whereas multiplicative fluctuations had a much larger effect. These results have a simple intuitive explanation. Variability in population activity reduces the ability to discriminate stimuli, when it means that a single population-firing pattern can be induced by two different sensory stimuli. A change in multiplicative gain can affect population activity in a very similar way to a change in stimulus contrast; it is thus not surprising that contrast discrimination is impaired strongly by multiplicative fluctuations. Likewise, the small effect of additive fluctuations may be understood from the fact that the resulting changes in baseline could not have been generated by any of the stimuli presented.

While correlated fluctuations were originally believed to only worsen stimulus discriminability (Zohary et al., 1994), responses with correlated variability can often outperform responses with the same amount of uncorrelated variability (Abbott and Dayan, 1999; Averbeck and Lee, 2006; Moreno-Bote et al., 2014). This does not mean that correlated variability helps discrimination (compared to zero variability); it means that its correlation structure interferes less with stimulus coding than might otherwise be expected. Indeed, we found that correlations frequently help discriminability. Shared additive fluctuations, for example, had a less detrimental effect on discriminability than their uncorrelated counterparts. This may be because the effect of shared additive fluctuations lies along a different direction in population vector space than differences between stimuli; shuffling thus adds variance to a dimension that can interfere with stimulus coding (Figure S8M). For shared multiplicative fluctuations, the effect of correlations on orientation discrimination depended on the difficulty of the discrimination task. For easy tasks (large orientation differences), correlations hurt. In this case, the pools of neurons responding to the two very different stimuli are largely distinct; the arguments of Zohary et al. (1994) therefore apply, and correlations cause averages over populations to be taken less accurately. For fine discrimination, however, correlations improved performance, possibly because the multiplicative fluctuations move population responses in a different direction to differences in stimulus orientation (akin to differences in contrast). Our decoding results therefore suggest that the nature of shared variability in visual cortical populations is well-suited for stimulus discrimination: additive variability has little consequence, while multiplicative-derived correlations benefit fine-orientation discrimination over uncorrelated variability.

But why should shared multiplicative and additive variability occur at all? Why not simply have private variability or no variability? Multiplicative variability might reflect the same circuit mechanisms that are responsible for top-down processes, such as attention. It has been suggested that visual attention modulates visual cortical responses in a similar manner to an increase in stimulus contrast, which could explain its primarily multiplicative effect similar to a contrast change (Reynolds and Heeger, 2009). While fluctuations in this process would clearly impair fine-contrast discrimination, contrast discrimination may be a task only rarely required. Indeed, much of the visual system appears to be geared toward making contrast-invariant judgments (Finn et al., 2007). Because our results show that multiplicative fluctuations have little effect on fine-orientation discrimination, we conclude that multiplicative fluctuations might allow the visual system to modulate the salience of visual stimuli, while having relatively little impact on stimulus coding. Additive fluctuations may allow the population to include an additional dimension of salience or other non-sensory factors, with only minor impact on representation of sensory information.

## Experimental Procedures

Anesthetized cat recordings were approved by the Animal Care and Use Committee of the Smith-Kettlewell Eye Research Institute. Experimental methods have been previously described by Busse et al. (2009). Briefly, responses, primarily from layers 2/3, were recorded with a 10-by-10 electrode array. All threshold crossings on each channel were pooled and only orientation-tuned sites were considered in subsequent analyses. Sequences of 2 s contrast-reversing oriented gratings and plaids, interspersed with 2 s blanks, were shown in random order in blocks (see Supplemental Information: anesthetized cat recordings).

Awake mouse recordings were conducted under personal and project licenses issued by the Home Office, in accordance with the UK Animals (Scientific Procedures) Act 1986. A head plate with a recording chamber was affixed to the skull. After 3 days of recovery and 3 head-restraint acclimatization sessions, a craniectomy (and durotomy, if necessary) was made over the left V1. The animal was allowed to recover for at least 1.5 hr before the recording. Multisite silicon probes were inserted to a depth of 500–800 μm (median 615 μm). Animals were judged to be quietly awake by video monitoring. Spikes were detected using NDManager (Hazan et al., 2006) and clustered using KlustaKwik (Harris et al., 2000; Kadir et al., 2014), followed by manual adjustment using KlustaViewa (Rossant and Harris, 2013). Detailed analysis was carried out only on well-isolated units that showed consistent firing throughout a recording session. Sequences of 1 s oriented drifting gratings, interspersed with either 1 or 6 s blanks were shown on three liquid-crystal display monitors, covering a field of view of ∼120° × 60° that extended in front and to the right of the animal (see Supplemental Information: awake mouse recordings).

Population tuning curves $R_{i}$ were fit to population responses to plaid stimuli as a linear combination of prototypical response to the component gratings and a constant baseline shift. The percentage of variance of the population activity that could be explained by the population tuning curve analysis on each trial was estimated as $1-\left(\sum_{c}{\left(r_{c,i}-R_{c,i}\right)}^{2}\right)/\left(\sum_{c}{\left(r_{c,i}-{\bar{r}}_{i}\right)}^{2}\right)$, where *r*_*c,i*_ is the normalized firing rate for each site *c* and trial *i*, and ${\bar{r}}_{i}$ is the measured site-averaged response on trial *i* (see Supplemental Information: population tuning curve analysis).

Full details of the models considered in this study can be found in Supplemental Information: the models. Briefly, we denote the experimentally measured spike counts of unit *c* (single neuron or multiunit) on trial *i* as *N*_*c,i*_ and the stimulus presented on trial *i* as *s*(*i*). Each model predicts an expected spike count *f*_*c,i*_ of unit *c* on trial *i* that approximates *N*_*c,i*_.

In the independent model, the expected spike count on each trial is a deterministic quantity:

(Equation 4) $$f_{c,i}=d_{c,s\left(i\right)},$$

where the matrix $d_{c,s\left(i\right)}$ is estimated as the trial-averaged spike count of unit *c* to stimulus *s*(*i*).

In the additive model, the expected spike count is

(Equation 5) $$f_{c,i}=d_{c,s\left(i\right)}+a_{i}h_{c},$$

where *d*_*c,s*_ represents the sensory drive of unit *c* from stimulus *s*, *a*_*i*_ the common additive offset on each trial *i*, and *h*_*c*_ the degree to which each unit *c* is susceptible to this offset. The total number of parameters in this model is $M_{\text{units}}M_{\text{stimuli}}+M_{\text{trials}}+M_{\text{units}}.\;d_{c,s}$ is estimated as in the independent model, and the parameters *a*_*i*_ and *h*_*c*_ were fit by least-squares.

In the multiplicative model, the expected spike count is given by

(Equation 6) $$f_{c,i}=g_{i}d_{c,s\left(i\right)}.$$

*g*_*i*_ is the common multiplicative gain on trial *i*. Note that unlike the model of Goris et al. (2014), where on each trial each cell has its own (private) gain (i.e., $f_{c,i}=g_{c,i}d_{c,s\left(i\right)}$), we propose a common multiplicative gain that is shared across the population. This model contains $M_{\text{units}}M_{\text{stimuli}}+M_{\text{trials}}$ parameters, which were fit by least-squares.

The affine model incorporates both the additive and multiplicative components:

(Equation 7) $$f_{c,i}=g_{i}d_{c,s\left(i\right)}+a_{i}h_{c}.$$

The total number of parameters in this model is $M_{\text{units}}M_{\text{stimuli}}+2M_{\text{trials}}+M_{\text{units}}$. We fit the affine model by an alternation method that was repeated until the convergence criterion was met, specifically that the difference in squared error per unit per trial $\sum_{c,i}{\left(f_{c,i}-N_{c,i}\right)}^{2}/M_{\text{units}}M_{\text{trials}}$ between two iterations was lower than 10^−10^. Typically this took several hundred iterations.

The predictions of these models represent expected spike counts rather than actual integer observations. To generate spike counts *n*_*c,i*_ from each model, we used a negative-binomial spike generator with mean *f*_*c,i*_ and a Fano factor parameter $F_{c,s\left(i\right)}$ that was estimated for each unit *c* and stimulus *s* by maximum likelihood (see Supplemental Information: spike count generator). Spike counts generated by this method were used for the analyses in Figures 3, 4, and 5.

We assessed the models’ goodness of fit by cross-validating the simulated spike count *n*_*c,i*_ (see Supplemental Information: cross-validation; Figure S1). Model performance was assessed for each unit *c* by quality index $q_{c}=1-\left(\sum_{i}e_{c,i}^{2}/\sum_{i}e_{c,i}^{\prime 2}\right)$, where $e_{c,i}^{\prime 2}$ was the squared error of the independent model. This cross-validation method was used to generate the plots in Figures 2E–2G, 6, and S2B–S2G; only units that showed shared variability (*q*_*c*_ > 0.1 for at least one of the models) were included in statistical analysis.

To estimate how the linear discriminability measure *d*^*2*^depends on the fluctuations of additive offset and multiplicative gain, we constructed a homogeneous neural population with translation-invariant orientation tuning curves (see Supplemental Information: decoding). To visualize the population patterns produced by this population, we simulated population responses on 10^4^ trials of the contrast and orientation discrimination tasks, assuming Gaussian distributions for both multiplicative gain and additive offset. The corresponding uncorrelated responses were obtained by trial-shuffling. These population responses were then projected onto a low-dimensional space for visualization (see Supplemental Information: decoding analysis with reduced dimensionality).

## Author Contributions

Conceptualization: I.-C.L., M.O., M.C., and K.D.H.; Methodology: I.-C.L., M.O., M.C., and K.D.H.; Software: I.-C.L.; Formal Analysis: I.-C.L., M.O., M.C., and K.D.H.; Investigation: I.-C.L. and M.O.; Resources: M.C.; Writing – Original Draft: I.-C.L., M.O., M.C., and K.D.H.; Writing – Reviewing & Editing: I.-C.L., M.O., M.C., and K.D.H.; Visualization: I.-C.L.; Supervision: M.C. and K.D.H.; Project Administration: M.C. and K.D.H.; Funding Acquisition: M.C. and K.D.H.

## Supplemental Information

Document S1. Supplemental Experimental Procedures, Figures S1–S8, and Tables S1–S4

Document S2. Article plus Supplemental Information
