# Supplementary material for: The Nature of Shared Cortical Variability
Source: Neuron. 2015 Aug 5;87(3):644–56. doi: 10.1016/j.neuron.2015.06.035 (PMC4534383; doi:10.1016/j.neuron.2015.06.035)
Supplement: Document S1. Supplemental Experimental Procedures, Figures S1–S8, and Tables S1–S4 [file mmc1.pdf]

Neuron

Supplemental Information

The Nature of Shared Cortical Variability

I-Chun Lin, Michael Okun, Matteo Carandini, and Kenneth D. Harris

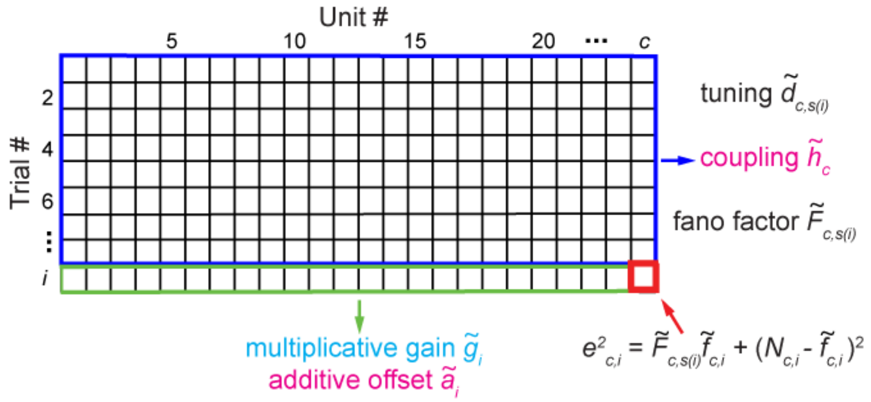

Figure S1 (related to Figures 2 and 6). Schematic of the cross-validation method used to evaluate the performance of the models for shared variability. We iterated over all elements of the matrix of units and trials, predicting the response of each element in the matrix from all other elements of the matrix. The predicted unit c is referred to as the 'test unit', and the predicted trial i is referred to as the 'test trial'. The remaining units were referred to as 'training units' and the remaining trials as 'training trials'. The data from all training trials (*blue block*) were used to estimate the stimulus-drive term $\tilde{d}_{c,s(i)}$, the fluctuation-coupling parameter $\tilde{h}_c$, and the Fano factor of the spike count generator $\tilde{F}_{c,s(i)}$. Then, the multiplicative gain $\tilde{g}_i$ and additive offset $\tilde{a}_i$ on the test trial were estimated from the activity of all training units on this trial (*green block*). Finally, a prediction $\tilde{f}_{c,i} = \tilde{g}_i\tilde{d}_{c,s(i)} + \tilde{a}_i\tilde{h}_c$ was obtained for the expected spike count of the test cell on the test trial. The squared error with respect to the test unit c on the test trial i (*red square*) was estimated as $e^2_{c,i} = \tilde{F}_{c,s(i)}\tilde{f}_{c,i} + \left(N_{c,i} - \tilde{f}_{c,i}\right)^2$ (see Supplemental Experimental Procedures: cross-validation). Cross-validated performance of each model was then assessed by quality index $q_c = 1 - \frac{\sum_i e^2_{c,i}}{\sum_i e'^2_{c,i}}$, where $e'^2_{c,i}$ is the squared error associated with the independent model.

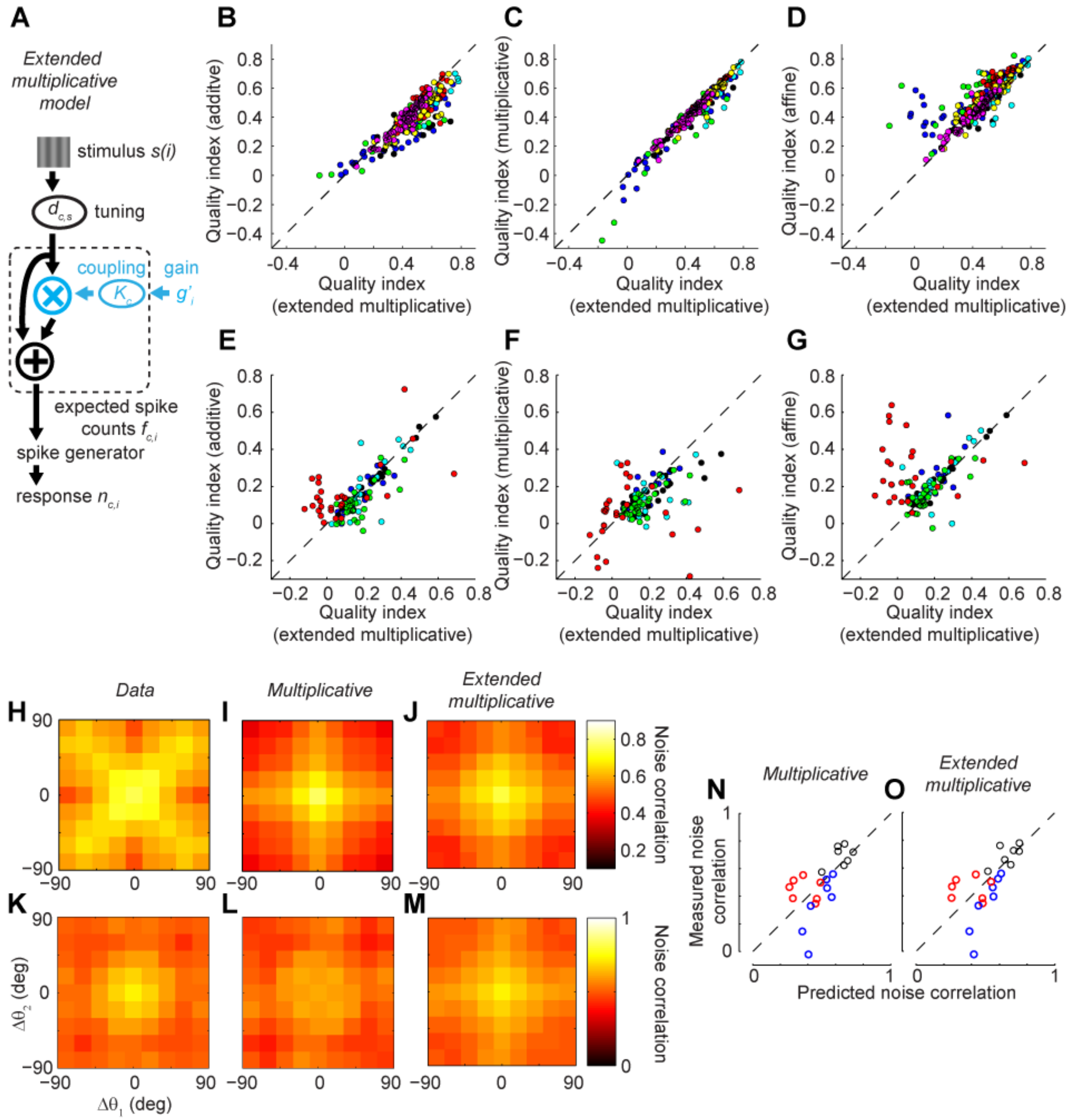

Figure S2 (related to Figures 2, 5, and 6). The extended multiplicative model. **(A)** In the extended multiplicative model, the expected spike count $f_{c,i}$ is given by $f_{c,i} = d_{c,s(i)}(1 + K_c g'_i)$. K_c determines how much a cell is coupled to the multiplicative gain g_i; K_c=0 reduces the model to the independent model, and K_c=1 to the multiplicative model with $g'_i = g_i - 1$. As in all other models, $f_{c,i}$ is passed into a stochastic spike count generator, which yields an integer spike count $n_{c,i}$ from a negative binomial distribution. (**B**-**D**) Cross-validated performance of the response $n_{c,i}$ generated by the extended multiplicative model versus the additive (B), multiplicative (C), and affine (D) models across 7 recording sessions in 3 anesthetized cats. Performance was measured by the quality index, which is zero or negative if the prediction is no better than the independent model, and equals 1 for a perfect prediction. Each *circle* represents the performance on one site across all trials in a session; sites from the same session share the

same color. Only sites that had quality index > 0.1 for at least one of the models were shown. (**E**-**G**) As in B-D, but for performance on single-unit data from quietly awake mouse recording (5 sessions in 4 mice). (**H**-**O**) The extended multiplicative model cannot capture the dependence of correlations on cell tuning and stimulus orientation in anesthetized cat data (cf. Figure 5). Pseudocolor representation of median noise correlation for all pairs of orientation-tuned sites as a function of $\Delta\theta_1$ and $\Delta\theta_2$ (H). Data were pooled across all contrasts and orientations. Session 83-7-5, 45 orientation-tuned sites. Predictions of the multiplicative and extended multiplicative models are plotted in I and J, respectively. Measured and predicted correlation matrices for session 83-10-15 (42 orientation-tuned sites) are shown in K-M; note the worse fit of the extended multiplicative than the affine models (see Figure 5). Scatter plots comparing the measured and predicted correlations for the multiplicative (N) and the extended multiplicative (O) models across 7 sessions in 3 cats; note again the poorer performance of the extended multiplicative than the affine models (Figure 5M). Each *circle* shows the measured and predicted noise correlations for one bin and one session; the median noise correlations in the center, corner, and edge bins are marked by *black*, *red*, and *blue circles*.

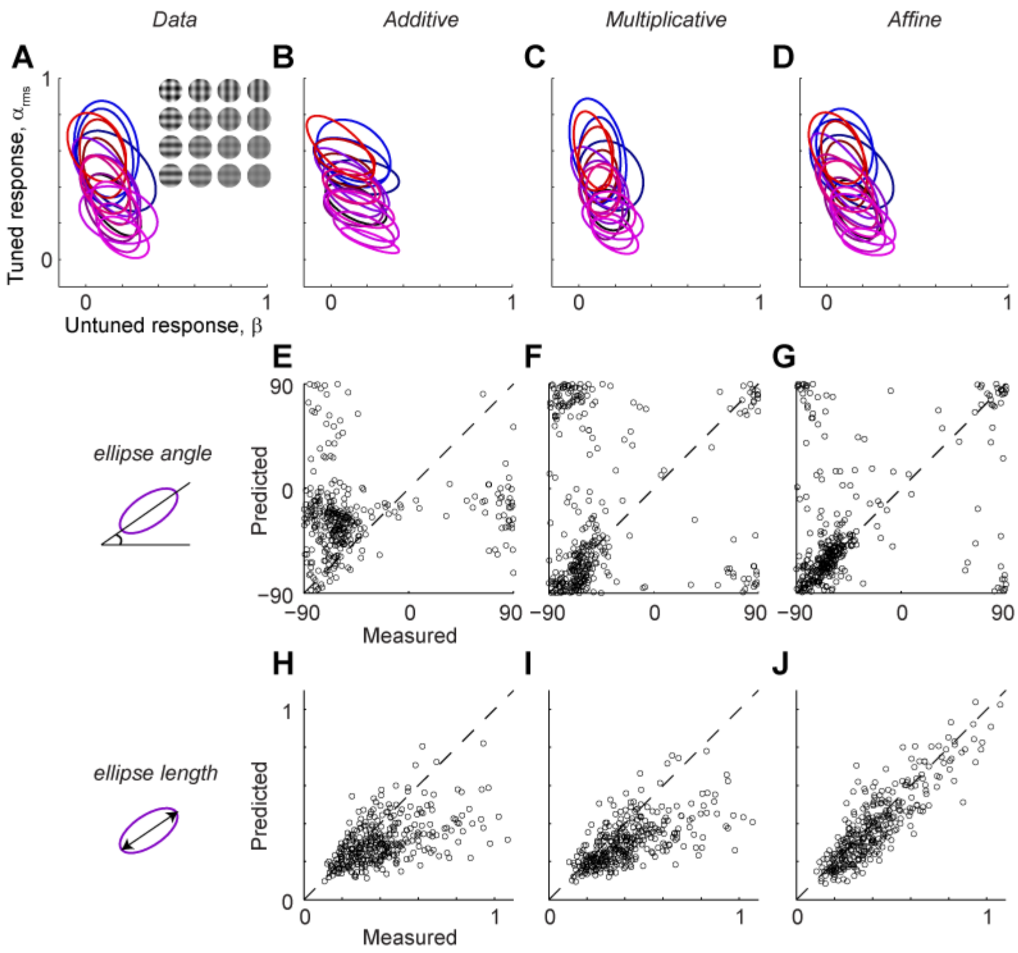

Figure S3 (related to Figure 3). Variability of population response to plaid stimuli. (**A**) Ellipses showing 1 SD contours of Gaussian fits to the distributions of the untuned response β and the average tuned response, defined as the root-mean square of the two tuned responses, $\alpha_{\mathrm{rms}} = \sqrt{\alpha_1^2 + \alpha_2^2}$, for an example recording session (session 83-7-5, 45 orientation-tuned sites, plaid angle = 90°). The 16 ellipses correspond to 16 plaid stimuli in which the contrasts of the two component gratings are varied independently. *Colors* indicate the component contrasts (RGB color code, *red* for contrast in grating 1, *blue* for grating 2). For each component-contrast combination, data were pooled across different component orientations. (**B**-**D**) Ellipses fitted to the predictions of the additive (B), multiplicative (C), and affine (D) models. (**E**-**G**) Comparison of the ellipse angles predicted by the three models versus experimental data across 7 sessions in 3 cats. Each plaid stimulus in each session contributes a *dot* for the ellipse fitted to the distribution of β versus α_{rms}. (**H**-**J**) Same as E-G, but for the comparison of the major-axis lengths of ellipses.

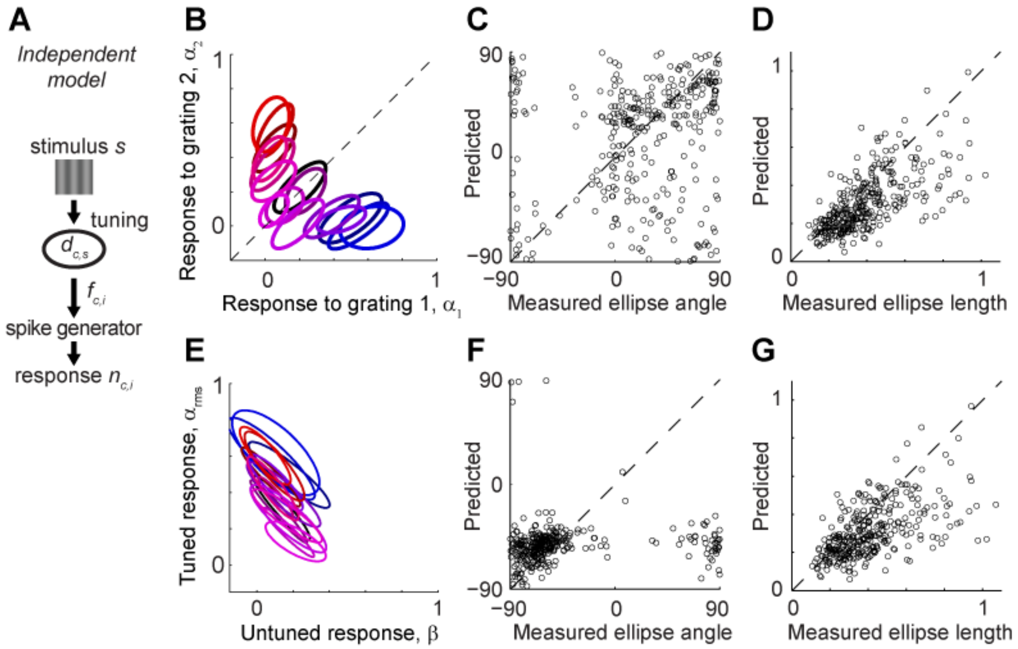

Figure S4 (related to Figure 3). The independent model. (**A**) In the independent model, the expected spike count $f_{c,i}$ is simply the trial-averaged spike count; as in other models, $f_{c,i}$ is passed into a stochastic spike count generator, yielding an integer spike count $n_{c,i}$ from a negative binomial distribution. (**B**) Ellipses showing 1 SD contours of Gaussian fits to the distributions of simulated population responses to gratings 1 and 2 (α_1 and α_2) for session 83-7-5 (45 orientation-tuned sites, plaid angle = 90°). The 16 ellipses correspond to 16 plaid stimuli in which the contrasts of the two component gratings are varied independently. *Colors* indicate the component contrasts (RGB color code with *red* encoding the contrast of grating 1 and *blue* encoding the contrast of grating 2). For each component-contrast combination, data were pooled across different component orientations. Note that a small upward tilt to the ellipses is seen; this does not result from correlated neuronal firing but rather from non-orthogonality of the basis vectors $\boldsymbol{G}(\theta_1)$ and $\boldsymbol{G}(\theta_2)$. (**C**) Comparison of the ellipse angles predicted by the independent model versus data from all 7 sessions in 3 cats. Each plaid stimulus in each session contributes a *dot* for the ellipse fitted to the distribution of α_1 vs α_2. (**D**) Same as C, but for the comparison of the major-axis lengths of ellipses. (**E**-**G**) As in B-D, but for the distributions of the untuned response β and the average tuned response, defined as the root-mean square of the two tuned responses, $\alpha_{\mathrm{rms}} = \sqrt{\alpha_1^2 + \alpha_2^2}$.

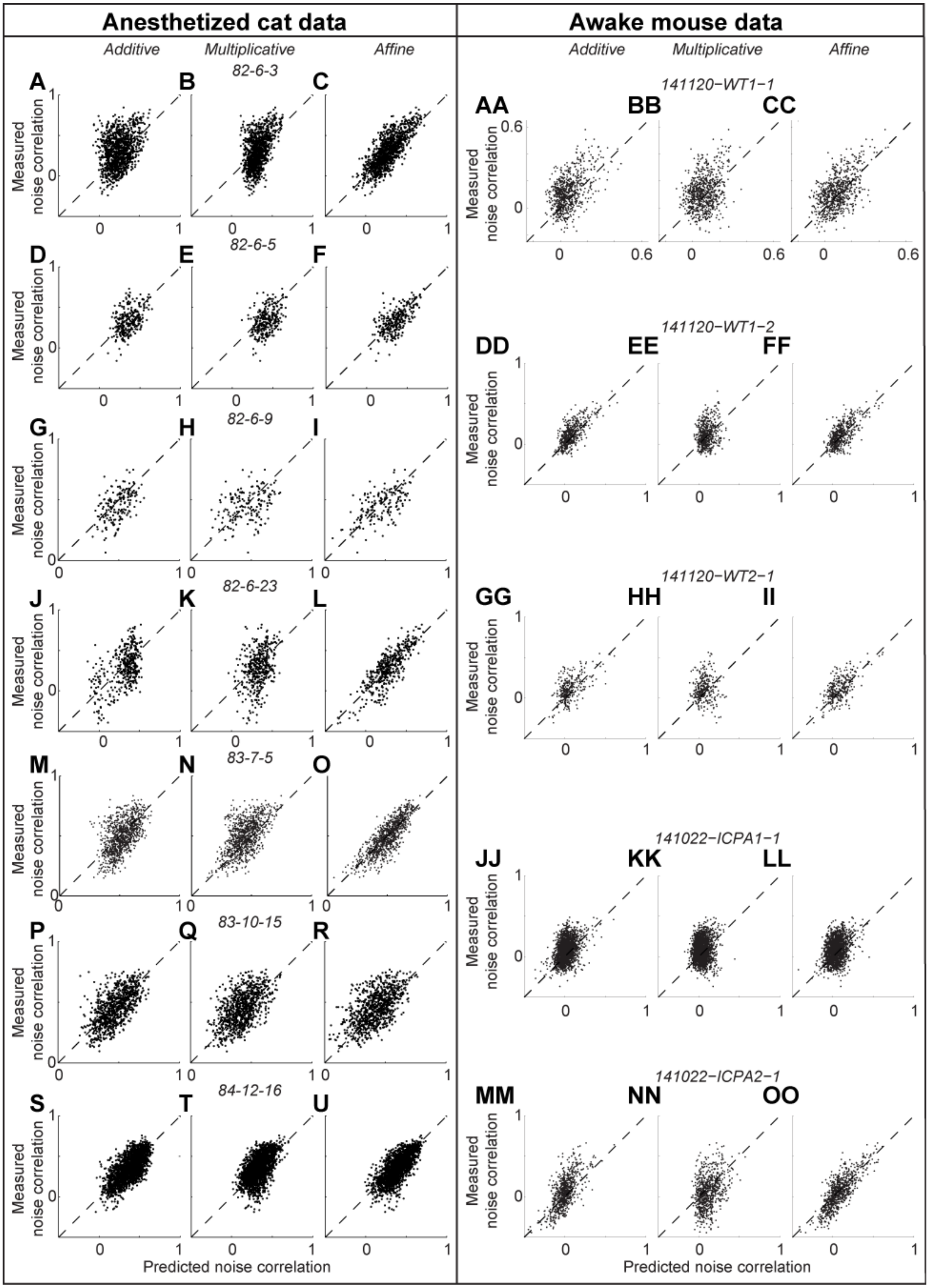

Anesthetized cat data
Awake mouse data
Additive
Multiplicative
Affine
82-6-3
82-6-5
82-6-9
82-6-23
83-7-5
83-10-15
84-12-16
141120−WT1−1
141120−WT1−2
141120−WT2−1
141022−ICPA1−1
141022−ICPA2−1
Measured noise correlation
Predicted noise correlation

Figure S5 (related to Figures 4 and 6). The affine model provides a better prediction of noise correlations in both anesthetized cat and quietly awake mouse data. (**A**-**C**) Measured noise correlations versus predictions from the additive (A), multiplicative (B), and affine (C) models for each pair of orientation-tuned sites in response to single gratings in anesthetized cat data. A *black dot* represents noise correlation for a pair of sites. Session 82-6-3, 47 orientation-tuned sites. (**D**-**F**) As in A-C, but for session 82-6-5, 24 orientation-tuned sites. (**G**-**I**) As in A-C, but for session 82-6-9, 19 orientation-tuned sites. (**J**-**L**) As in A-C, but for session 82-6-23, 29 orientation-tuned sites. (**M**-**O**) As in A-C, but for session 83-7-5, 45 orientation-tuned sites. (**P**-**R**) As in A-C, but for session 83-10-15, 42 orientation-tuned sites. (**S**-**U**) As in A-C, but for session 84-12-16, 64 orientation-tuned sites. (**AA-CC**) Measured noise correlations versus predictions from the additive (AA), multiplicative (BB), and affine (CC) models for each pair of neurons in response to single gratings in quietly awake mouse data. A *black dot* represents noise correlation for a pair of neurons. Session 141120-WT1-1, 39 stable, well-isolated neurons. (**DD**-**FF**) As in AA-CC, but for session 141120-WT1-2, 33 well-isolated neurons. (**GG**-**II**) As in AA-CC, but for session 141120-WT2-1, 29 well-isolated neurons. (**JJ**-**LL**) As in AA-CC, but for session 141022-ICPA1-1, 66 well-isolated neurons. (**MM**-**OO**) As in AA-CC, but for session 141022-ICPA2-1, 39 well-isolated neurons.

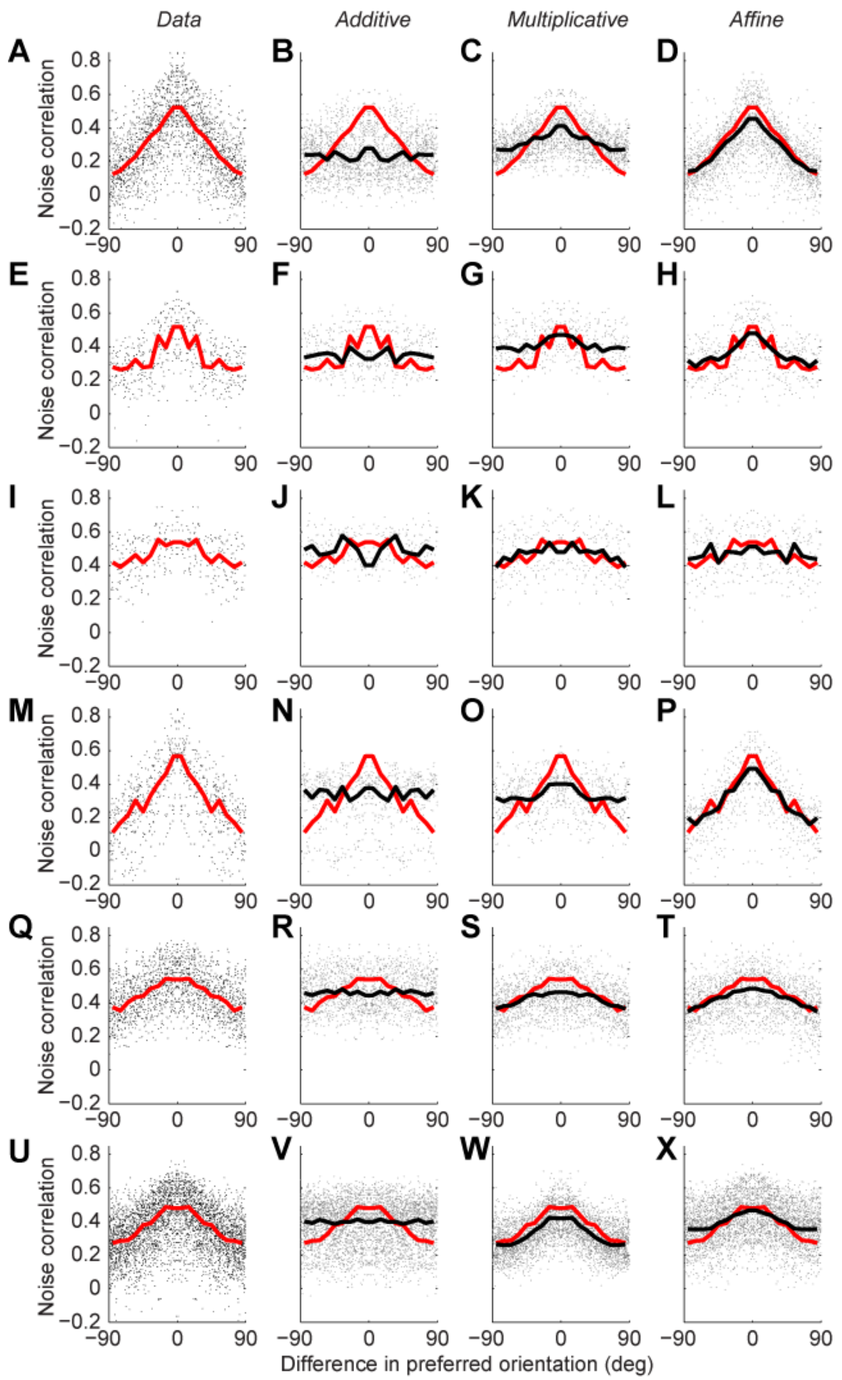

Figure S6 (related to Figure 4). Relationship between noise correlation and tuning similarity in anesthetized cat data. (**A**) Noise correlation for each pair of orientation-tuned sites in response to single gratings as a function of the difference between their preferred orientations. A *black dot* represents noise correlation calculated for a pair of sites, and the *red curve* shows the running median. (**B**-**D**) As in A, but for predictions of the additive (B), multiplicative (C), and affine (D) models. A *gray dot* represents noise correlation predicted by the model for a pair of sites, and a *black curve* shows the running median; a *red curve* repeats the running median of the measured data for comparison. All running medians were calculated with non-overlapping 10° bins. Session 82-6-3, 47 orientation-tuned sites. (**E**-**H**) As in A-D, but

for session 82-6-5, 24 orientation-tuned sites. (**I**-**L**) As in A-D, but for session 82-6-9, 19 orientation-tuned sites. (**M**-**P**) As in A-D, but for session 82-6-23, 29 orientation-tuned sites. (**Q**-**T**) As in A-D, but for session 83-10-15, 42 orientation-tuned sites. (**U**-**X**) As in A-D, but for session 84-12-16, 64 orientation-tuned sites.

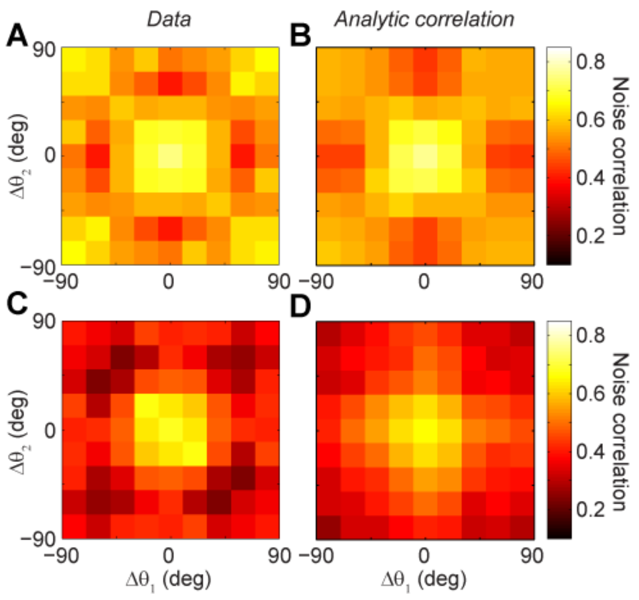

Figure S7 (related to Figure 5). The structures of correlation matrices can be analytically predicted from parameters of the affine model. (**A**) Experimentally measured correlation matrices for recording session 83-7-5 of anesthetized cat data. Here we only considered responses to single gratings at 50% contrast. (**B**) Correlation matrices computed from Eqs. S12, S15, and S16, with the parameters $d_{c,s}$, h_c, $F_{c,s}$, and statistical quantities $E(g_i)$, $Var(g_i)$, $E(a_i)$, $Var(a_i)$, $Cov(a_i, g_i)$ estimated from session 83-7-5. (**C**,**D**) Same as A,B, but for session 83-10-15.

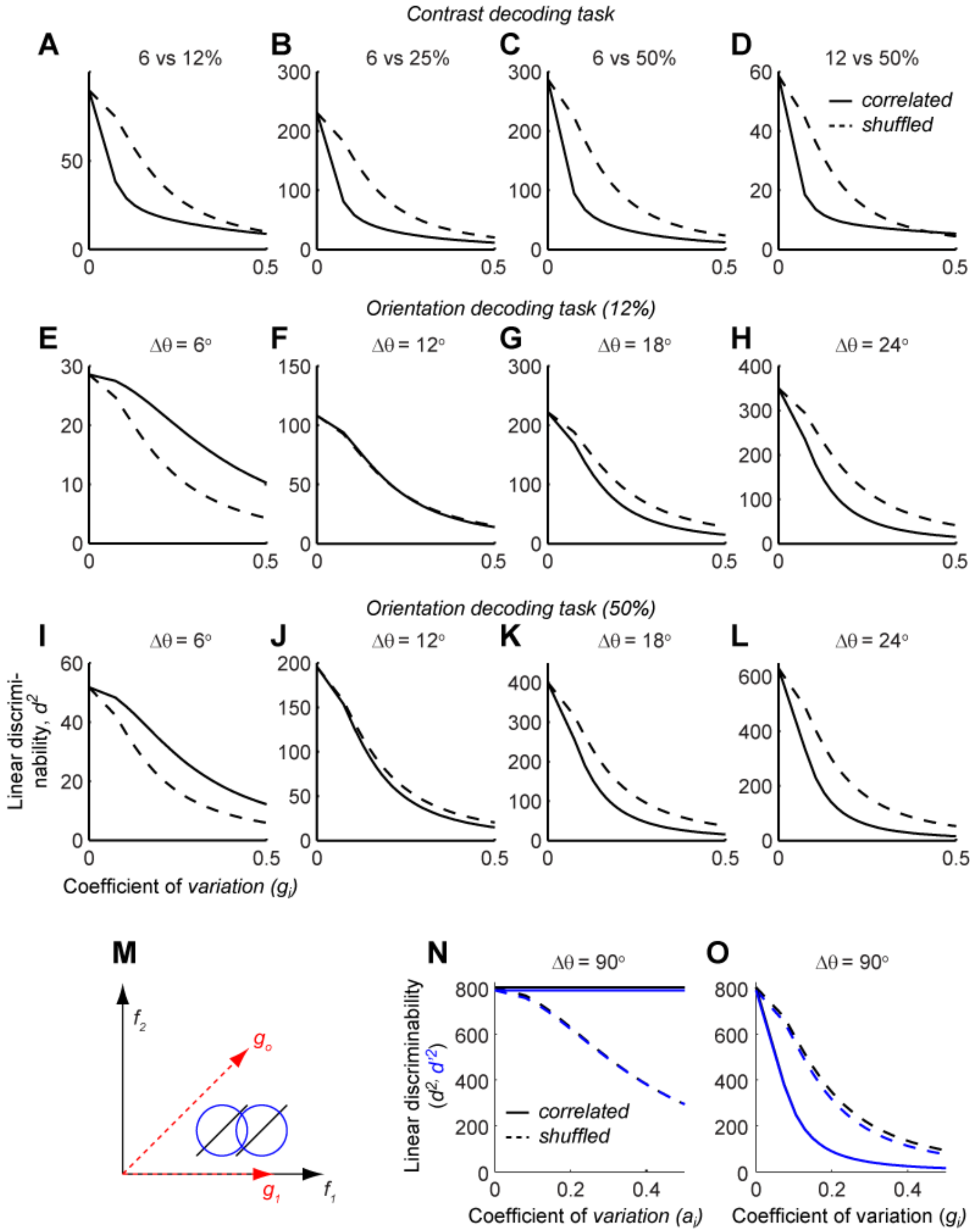

Figure S8 (related to Figures 7 and 8). Further analyses of the effects of fluctuations on stimulus coding. (**A-D**) The discriminability measure d^2 (*solid line*) and the corresponding d^2_{shuffled} (*dashed line*) as a function of the coefficient of variation of the multiplicative gain in four different contrast decoding tasks: two gratings of the same orientation at 6% and 12% (A), 6% and 25% (B), 6% and 50% (C), and 12% and 50% (D) contrasts. Note that trial-shuffling improves discrimination performance for all comparisons. (**E-H**) As in A-D, but for orientation discrimination tasks that involve distinguishing two 12%-contrast gratings whose orientations differed by 6° (E), 12° (F), 18° (G), and 24° (H). (**I-L**) As in E-H, but for tasks involving discerning two 50%-contrast gratings whose orientations differed by 6° (I), 12° (J), 18° (K), and 24° (L). Note that in orientation discrimination tasks, regardless of the stimulus contrast, trial-shuffling worsens discriminability for fine orientation discrimination (see also Figure 8E), but improves it for angles greater

than 12°. (**M**) Cartoon illustrating how shuffling of additive fluctuations worsens discriminability in a contrast discrimination task. The *x*- and *y*-axes represent the spike counts of two neurons, with f_1 representing a neuron that responds to the presented stimulus, and f_2 representing a neuron that responds to a different stimulus but is still modulated by additive variability. (For simplicity, here we consider the expected spike counts without passing through the spike count generator.) The vectors g_0 and g_1 correspond to an additive baseline shift and the tuned response to the grating. The two *black lines* show the distributions of responses to stimuli 1 and 2 if additive-correlated variability is present. Note that this variability is in a different direction to the difference between the mean stimulus responses, yielding two lines that are both parallel to g_0. After shuffling, these distributions become circular (*blue circles*), introducing a considerable overlap between the two distributions, thus reducing d^2. (**N**,**O**) Comparison between the linear discriminability measure d^2 (*black solid line*) as well as d^2_{shuffled} (*black dashed line*) calculated in the full high-dimensional response space and their corresponding approximations d'^2 (*blue solid line*) as well as d'^2_{shuffled} (*blue dashed line*) calculated in the 2-dimensional spaces used for visualization in Figures 7 and 8. Examples shown here are from one of the orientation discrimination tasks plotted in Figure 8. The similar shapes of the blue and black curves indicate that the projections illustrated in Figures 7 and 8 accurately capture the separation of high-dimensional ellipsoids in the full response space.

Session ID	*nu*	*nrpt*	*plaid angle*	*nu'*	*p*: additive vs multiplicative	*p*: additive vs affine	*p*: multiplicative vs affine
82-6-3	47	10	90°	46	$5.4\text{x}10^{-4}$ (multiplicative)	$2.8\text{x}10^{-14}$ (affine)	$1.3\text{x}10^{-12}$ (affine)
82-6-5	24	10	30°	24	$6.6\text{x}10^{-3}$ (multiplicative)	$6.6\text{x}10^{-3}$ (affine)	$2.8\text{x}10^{-4}$ (affine)
82-6-9	19	8	45°	19	$1.7\text{x}10^{-1}$	$1.9\text{x}10^{-2}$ (affine)	$7.6\text{x}10^{-5}$ (affine)
82-6-23	29	10	45°	29	$2.4\text{x}10^{-2}$ (multiplicative)	$3.7\text{x}10^{-9}$ (affine)	$3.7\text{x}10^{-9}$ (affine)
83-7-5	45	10	90°	45	$3.7\text{x}10^{-1}$	$2.6\text{x}10^{-12}$ (affine)	$5.7\text{x}10^{-14}$ (affine)
83-10-15	42	7	90°	42	$2.7\text{x}10^{-4}$ (multiplicative)	$4.4\text{x}10^{-7}$ (affine)	$6.9\text{x}10^{-5}$ (affine)
84-12-16	64	6	60°	60	$1.1\text{x}10^{-3}$ (multiplicative)	$6.3\text{x}10^{-14}$ (affine)	$9.7\text{x}10^{-11}$ (affine)

Table S1 (related to Figure 2). Session ID, number of orientation-tuned units (*nu*), number of repeats per stimulus (*nrpt*), plaid angle, number of sites that showed considerable shared variability (*nu'*, quality index $q > 0.1$ for at least one of the models in cross-validation analysis) in each of the 7 recording sessions in anesthetized cat data. The last 3 columns tabulated the *p* value of the two-sided sign test on quality index of each model in cross-validation analysis. For ones that have $p < 0.05$, the model that has a greater median *q* (i.e., the better model) is indicated in bracket.

Session ID	*p*: additive vs extended multiplicative	*p*: multiplicative vs extended multiplicative	*p*: affine vs extended multiplicative
82-6-3	$5.4\text{x}10^{-4}$ (extended multi)	$1.6\text{x}10^{-3}$ (extended multi)	$4.6\text{x}10^{-10}$ (affine)
82-6-5	$6.6\text{x}10^{-3}$ (extended multi)	$2.8\text{x}10^{-4}$ (extended multi)	$2.3\text{x}10^{-2}$ (affine)
82-6-9	$7.6\text{x}10^{-5}$ (extended multi)	$7.3\text{x}10^{-4}$ (extended multi)	1
82-6-23	$2.3\text{x}10^{-3}$ (extended multi)	$6.1\text{x}10^{-2}$	$1.6\text{x}10^{-6}$ (affine)
83-7-5	$2.5\text{x}10^{-3}$ (extended multi)	$3.1\text{x}10^{-6}$ (extended multi)	$9.3\text{x}10^{-9}$ (affine)
83-10-15	$5.6\text{x}10^{-9}$ (extended multi)	$7.9\text{x}10^{-3}$ (extended multi)	$4.4\text{x}10^{-2}$ (affine)
84-12-16	$1.6\text{x}10^{-7}$ (extended multi)	$2.7\text{x}10^{-2}$ (extended multi)	$1.2\text{x}10^{-5}$ (affine)

Table S2 (related to Figure 2). Session ID and the *p* value of the two-sided sign test on quality index of each model versus the extended multiplicative model in cross-validation analysis in each of the 7 recording sessions in anesthetized cat data. For ones that have $p < 0.05$, the model that has a greater median *q* (i.e., the better model) is indicated in bracket. Number of neurons that showed considerable shared variability (quality index $q > 0.1$ in for at least one of the models in cross-validation analysis) for each session was as in Table S1.

Session ID	*nu*	*nrpt*	*nu'*	*p*: additive vs multiplicative	*p*: additive vs affine	*p*: multiplicative vs affine
141120-WT1-1	39	35	22	5.3×10^{-2}	4.3×10^{-3} (affine)	8.6×10^{-4} (affine)
141120-WT1-2	33	50	21	7.8×10^{-2}	1.5×10^{-3} (affine)	2.2×10^{-4} (affine)
141120-WT2-1	29	35	18	2.4×10^{-1}	7.5×10^{-3} (affine)	1.3×10^{-3} (affine)
141022-PA1-1	66	15	26	8.5×10^{-1}	5.3×10^{-4} (affine)	1.0×10^{-5} (affine)
141022-PA2-1	39	15	27	1.2×10^{-1}	4.9×10^{-5} (affine)	4.9×10^{-5} (affine)

Table S3 (related to Figure 6). Session ID, number of stable, well-isolated neurons (*nu*), number of repeats per stimulus (*nrpt*), and number of neurons that showed considerable shared variability (*nu'*, quality index $q > 0.1$ in for at least one of the models in cross-validation analysis) in each of the 5 recording sessions in quietly awake mice. The last 3 columns tabulated the *p* value of the two-sided sign test on quality index of each model in cross-validation analysis. For ones that have $p < 0.05$, the model that has a greater median *q* (i.e., the better model) is indicated in bracket.

Session ID	*nu'*	*p*: additive vs extended multiplicative	*p*: multiplicative vs extended multiplicative	*p*: affine vs extended multiplicative
141120-WT1-1	22	2.9×10^{-1}	1	1.7×10^{-2} (affine)
141120-WT1-2	22	1.7×10^{-2} (extended multi)	1.7×10^{-2} (extended multi)	2.9×10^{-1}
141120-WT2-1	19	1	6.4×10^{-2}	4.4×10^{-3} (affine)
141022-PA1-1	30	6.0×10^{-5} (extended multi)	3.3×10^{-4} (extended multi)	8.6×10^{-1}
141022-PA2-1	27	5.9×10^{-3} (additive)	2.5×10^{-1}	1.5×10^{-3} (affine)

Table S4 (related to Figure 6). Session ID, number of neurons that showed considerable shared variability (*nu'*, quality index $q > 0.1$ in for at least one of the models in cross-validation analysis), and the *p* value of the two-sided sign test on quality index of each model versus the extended multiplicative model in cross-validation analysis in each of the 5 recording sessions in quietly awake mouse data. For ones that have *p* < 0.05, the model that has a greater median *q* (i.e., the better model) is indicated in bracket.

Supplemental Experimental Procedures

Anesthetized cat recordings

The data consisted of 7 recording sessions from 3 different neuronal populations in 3 cats (Busse et al., 2009). In short, young adult cats were anesthetized with ketamine and xylazine for initial surgery, followed by sodium pentothal and fentanyl for electrophysiological recordings. Responses were recorded from a 10-by-10 electrode array (Figure 1A, 400 μm spacing, 1.5 mm electrode length) with a Cerebus data acquisition system (Blackrock Microsystems, USA). The recordings were estimated to be primarily from layers 2/3 based on insertion depth.

Spikes were detected independently for each site of the array. For each site, we computed a threshold of 4 SD of the background noise. All threshold crossings on each channel were pooled, and only sites that showed orientation tuning were considered in subsequent analyses. Two criteria were used: first, the circular variance of the site's orientation tuning needed to be less than 0.9; second, the site's response (raw spike counts) to its preferred orientation needed to be at least twice that to the orthogonal stimulus at 100% contrast. On average, 42 ± 13 (median ± MAD) sites per recording passed the criteria.

Blanks were gray screen periods with the same mean luminance as the gratings. The gratings were circular (30° diameter), varying in orientation (6 orientations) and contrast (6%, 12%, 25%, and 50%), and of 4 Hz temporal frequency. Spatial frequency was adjusted to optimally drive the majority of sites in the array. Plaids were generated by summing two gratings. The plaid angle (the angle between the two component orientations) was fixed in each session, and plaids in each session consisted of three pairs of orientations (e.g., $0^o/90^o$ and $30^o/120^o$ for a plaid angle of 90°) to reduce the effect of adaptation. To obtain orientation tuning curves, responses to 100% contrast gratings of 12 different orientations were recorded. Stimuli were shown in random order in blocks and presented at least 6 times (Table S1).

Awake mouse recordings

We recorded from two C57BL/6J mice (8 week-old males) and two PV-Cre x Ai32 transgenic mice (11 week-old males; no optogenetic stimuli were applied for these experiments). Each mouse was implanted with a custom-built head plate with a recording chamber under isoflurane anesthesia. After 3 days of recovery accompanied by Rimadyl treatment and 3 head-restraint acclimatization sessions, the animal was briefly anesthetized with isoflurane and a ~1mm^2 craniectomy was made over the left V1 on the first recording day. The dura was resected with a 30G needle when necessary, and the brain was covered with cortex buffer and sealed using Kwik-Cast (World Precision Instruments, Inc.). If an additional recording was carried out the following day, the brain was again protected with cortex buffer and Kwik-Cast. There were five recording sessions in total, of which three were in the wild-type mice and two in the transgenic mice. Recordings were made with a Buzsaki32 silicon probe (NeuroNexus technologies, USA) that was lowered to a depth of 500-800 μm (median 615 μm) by a PatchStar manipulator (Scientifica, UK). Signals were amplified and stored for offline analysis using the Cerebus data acquisition system. During recording, the animal stood in a custom-built tube and were judged to be quietly awake by video monitoring (open eyes, sporadic whisking, postural adjustments, and other movements).

Detailed analysis was carried out only on well-isolated units (isolation distance > 20; refractory violation probability < 0.001 (Harris et al., 2001; Schmitzer-Torbert et al., 2005)) that showed consistent firing throughout a recording session (rate > 1 spike/trial). On average, 39 ± 6 (median ± MAD) neurons per session passed the criteria.

Stimuli were sequences of 1 s oriented drifting gratings (100% contrast, 2 Hz, 12 directions, and spatial frequency of 0.05 cpd), interspersed with either 1 s or 6 s blanks. Stimuli were presented 15-50 times in random order in blocks (Table S3). For sessions 141022-PA1-1 and 141022-PA1-2, stimuli of 60% contrast were also presented.

Population tuning curve analysis

To characterize population responses to plaid stimuli, we first computed each site's total spike count and firing rate on each trial. We then normalized each site's firing rate by dividing by the maximum of its fitted orientation tuning curve. Finally, we combined the results into a population response vector $\boldsymbol{r_i}$ that summarized the normalized firing rates of all orientation-tuned sites on trial i. We used least-squares estimation to fit a population tuning curve $\boldsymbol{R}_i$ to the measured data $\boldsymbol{r_i}$ on trial i as a linear combination of prototypical responses to the component gratings, plus a constant baseline shift:

$$\boldsymbol{R}_i = \alpha_1^i \boldsymbol{G}(\theta_1^i) + \alpha_2^i \boldsymbol{G}(\theta_2^i) + \beta^i \mathbf{1}. \qquad (S1)$$

θ_1^i and θ_2^i are the component orientations of the plaid stimulus presented on trial i, $\boldsymbol{G}(\theta)$ represents the prototypical response to a grating of orientation θ, $\mathbf{1}$ represents the constant vector (all entries 1), and α_1^i, α_2^i, and β^i are weights estimated on trial i. The function $\boldsymbol{G}(\theta)$ was estimated from all presentations of single-grating stimuli as a unit circular Gaussian whose vertical offset and width are set as the baseline and the width of the mean single-site orientation response profile averaged across all orientation-tuned sites. Using this formula, the population tuning curve on trial i can be summarized using only three parameters: α_1^i, α_2^i, and β^i (Figure 1B).

The models

We denote the experimentally measured spike counts of unit c (single neuron or multiunit) on trial i as $N_{c,i}$ and the stimulus presented on trial i as $s(i)$. The independent, additive, multiplicative, and affine models predict an expected spike count $f_{c,i}$ of unit c on trial i that approximates $N_{c,i}$.

In the additive model (Figure 2B), the expected spike count is

$$f_{c,i} = d_{c,s(i)} + a_i h_c. \qquad (S2)$$

The matrix $d_{c,s}$ (of size $M_{\text{units}} \times M_{\text{stimuli}}$) is estimated as the mean response of unit c over all presentations of stimulus s. All of the $M_{\text{units}} \times M_{\text{stimuli}}$ elements of this matrix represent parameters of the model that are fit to the data. The matrix $d_{c,s(i)}$ that appears in Eq. S2 has size $M_{\text{units}} \times M_{\text{trials}}$, and is generated by concatenating the columns of $d_{c,s}$ in the order $s(i)$. The M_{trials}-dimensional vector a_i represents the amount of common additive offset on each trial i, and the M_{units}-dimensional vector h_c represents the degree to which each unit c is susceptible to this offset. The parameters a_i and h_c were fit by least-squares, computed by keeping the first term in a singular value decomposition of the residual matrix $N_{c,i} - d_{c,s(i)}$.

In the multiplicative model (Figure 2C), the expected spike count is given by

$$f_{c,i} = g_i d_{c,s(i)}. \qquad (S3)$$

The M_{trials}-dimensional vector g_i is the amount of common multiplicative gain on each trial i. The parameters g_i and $d_{c,s(i)}$ were fit by least-squares: for every stimulus s, we estimated the M_{units}-dimensional vector corresponding to the sth column of $d_{c,s}$ and the M_{repeats}-dimensional subvector of g_i

corresponding to the repeats of stimulus s by keeping the first term in a singular value decomposition of the $M_{\text{units}} \times M_{\text{repeats}}$-sized submatrix of $f_{c,i}$ that contains the observed spike counts in response to all repeats of stimulus s.

The affine model (Figure 2D) includes both the additive and multiplicative components:

$$f_{c,i} = g_i d_{c,s(i)} + a_i h_c. \qquad \text{(S4)}$$

We fit the affine model by an alternation method. The additive terms a_i and h_c were computed as the first term in the singular value decomposition of the residual matrix $N_{c,i} - g_i d_{c,s(i)}$; the multiplicative terms g_i and $d_{c,s(i)}$ were then fit as the first term in the singular value decomposition of the residual $N_{c,i} - a_i h_c$ according to the method described for the multiplicative model.

Note that the parameterization of the affine model is underdetermined. Specifically, given any fit of this model, one can generate an equivalent fit for any real number γ:

$$d_{c,s(i)} = d'_{c,s(i)} - \gamma h_c, \qquad \text{(S5)}$$

$$a_i = a'_i + \gamma g_i. \qquad \text{(S6)}$$

To remove this ambiguity we chose the unique value of γ for which d_{c,s_0} (i.e., the modeled sensory drive induced by blanks) was the average spontaneous activity of unit c in a blank trial.

We found that the matrices $d_{c,s}$ fit for the additive, multiplicative, and affine models were very similar (the same also held true for h_c fit for the additive and affine models; data not shown).

Spike count generator

The model predictions are not integers, and they represent expected spike counts rather than actual potential observations. It is common in neuronal response modeling to simulate actual spike counts according to a Poisson distribution, the variability of which captures "private noise" individual to each neuron. Nevertheless, there is nothing in neuronal biophysics that suggests neurons should produce Poisson variability, and a Poisson distribution may underestimate the amount of private variability in each neuron. To generate spike counts $n_{c,i}$ from the model, we thus used a negative binomial distribution, a more general family of count distributions to which the Poisson belongs:

$$n_{c,i} \sim NB\left(f_{c,i}, F_{c,s(i)}\right). \qquad \text{(S7)}$$

The negative binomial distribution has two parameters and can be characterized by an expected spike count f and Fano factor F. (Note that the negative binomial distribution can be parameterized in multiple ways; another common parameterization would be $p = 1 - \frac{1}{F}$ and $r = \frac{f(1-p)}{p}$.) The negative binomial reduces to a Poisson distribution when F is 1, but it has higher variance than Poisson for $F > 1$. After computing the expected spike counts $f_{c,i}$ as described above, we estimated a Fano factor $F_{c,s}$ for each unit c and stimulus s by maximum likelihood. Specifically, we used the simplex algorithm ('fminsearch' function in Matlab) to search for the value of $F_{c,s}$ that minimized the negative log likelihood of the full set of observed spike counts.

Cross-validation

We assessed the models' goodness of fit by cross-validating the simulated spike count $n_{c,i}$ (Figure S1). Because of the way the model was parameterized, this required a specialized approach. Specifically, we

looped over all units and trials to predict the response of unit c on trial i from all other elements of the spike count matrix. Unit c is termed the 'test unit', and trial i is termed the 'test trial'. The remaining units were referred to as 'training units', and the remaining trials as 'training trials'. The data from all training trials was used to estimate the stimulus-drive and fluctuation-coupling parameters $\tilde{d}_{c,s(i)}$ and $\tilde{h}_c$ as well as the unit- and stimulus-dependent Fano factor $\tilde{F}_{c,s(i)}$. Then, the multiplicative gain $\tilde{g}_i$ and additive offset $\tilde{a}_i$ on the test trial were estimated from the activity of all training units on this trial. Finally, a prediction $\tilde{f}_{c,i} = \tilde{g}_i \tilde{d}_{c,s(i)} + \tilde{a}_i \tilde{h}_c$ was obtained for the expected spike count of the test unit on the test trial. Note that this prediction used all other elements of the response matrix except the unit and trial being predicted. To obtain more accurate results, we analytically compute the expected cross-validation error over the distribution produced by the spike count generator of Eq. S7 (i.e., the private variability), while considering a fixed value of $f_{c,i}$ and $N_{c,i}$ as well as with all expectations and variances defined by the probability distribution of Eq. S7. The squared error with respect to the test unit c on test trial i can be computed as:

$$\begin{aligned} e_{c,i}^2 &= E\left(\left(n_{c,i} - N_{c,i}\right)^2\right) \\ &= E\left(\left(n_{c,i} - E\left(n_{c,i}\right)\right)^2\right) + \left(E\left(n_{c,i}\right) - N_{c,i}\right)^2 \\ &= Var\left(n_{c,i}\right) + \left(E\left(n_{c,i}\right) - N_{c,i}\right)^2 \\ &= F_{c,s}E\left(f_{c,i}\right) + Var\left(f_{c,i}\right) + \left(f_{c,i} - N_{c,i}\right)^2 \\ &= F_{c,s}f_{c,i} + \left(f_{c,i} - N_{c,i}\right)^2, \end{aligned} \qquad (S8)$$

using Eq. S12 and the fact that $Var\left(f_{c,i}\right) = 0$ (since here we consider $f_{c,i}$ as a fixed value). The first term in the last line is the contribution from the stochastic spike count generator to the squared error; the second term is the error from the spike estimate derived from each of the models.

Analytic calculation of pairwise correlations

Here we compute the correlations expected from the affine model. In this calculation, in addition to stochastic spike generation (private variability, Eq. S7), we consider g_i and a_i to be random variables; the only aspects of their probability distributions that make a difference to the pairwise neuronal correlations are their means, variances, and covariances.

To analytically derive the correlations expected under this model, we first compute the expected value and variance of $f_{c,i}$ during repeated presentations of stimulus s:

$$E\left(f_{c,i}\right) = E(g_i)d_{c,s} + E(a_i)h_c, \qquad (S9)$$

$$Var\left(f_{c,i}\right) = Var(g_i)d_{c,s}^2 + Var(a_i)h_c^2 + 2Cov(g_i, a_i)d_{c,s}h_c. \qquad (S10)$$

Similarly, the covariance of the expected spike counts for two units is

$$Cov\left(f_{c_1,i}, f_{c_2,i}\right) = Var(g_i)d_{c_1,s}d_{c_2,s} + h_{c_1}h_{c_2}Var(a_i) + \left(h_{c_1}d_{c_2,s} + h_{c_2}d_{c_1,s}\right)Cov(g_i, a_i). \qquad (S11)$$

We next compute the variance of spike counts $n_{c,i}$ for unit c. To do this, we employ the law of total variance, which holds that for random variables X and Y, $Var(Y) = E\left(Var(Y|X)\right) + Var(E(Y|X))$. Using this with $X = f_{c,i}$ and $Y = n_{c,i}$, we have

$$Var\left(n_{c,i}\right) = E\left(Var\left(n_{c,i}|f_{c,i}\right)\right) + Var\left(E\left(n_{c,i}|f_{c,i}\right)\right) = F_{c,s}E\left(f_{c,i}\right) + Var\left(f_{c,i}\right). \qquad (S12)$$

All variances and expectations are over repeated presentations of a single stimulus s. This can be interpreted as a sum of two terms: the first corresponding to the amount of private variance expected due to the neuron's spike generator, and the second corresponding to the amount of variance expected due to shared population variability.

Similarly, to compute the covariance of two cells c_1 and c_2, we employ the law of total covariance: $Cov(X,Y) = Cov\big(E(X|Z), E(Y|Z)\big) + E\big(Cov(X,Y|Z)\big)$. To use this formula, we let X denote $n_{c_1,i}$, let Y denote $n_{c_2,i}$, and let Z denote the compound random variable $\{f_{c_1,i}, f_{c_2,i}\}$, then we have $E(X|Z)$=$f_{c_1,i}$, $E(Y|Z)$=$f_{c_2,i}$, and

$$Cov\big(E(X|Z), E(Y|Z)\big) = Cov\big(f_{c_1,i}, f_{c_2,i}\big). \qquad (S13)$$

To compute $E\big(Cov(X,Y|Z)\big)$, we note that the spike generator of each neuron is conditionally independent. Thus,

$$\begin{aligned} &E\left(Cov\big(n_{c_1,i}, n_{c_2,i}\big|f_{c_1,i}, f_{c_2,i}\big)\right) \\ &= \delta_{c_1c_2} E\left(Var\big(n_{c_1,i}\big|f_{c_1,i}\big)\right) \\ &= \delta_{c_1c_2} E\big(F_{c_1,s} f_{c_1,i}\big) \\ &= \delta_{c_1c_2} F_{c_1,s}\big(E(g_i)d_{c_1,s} + E(a_i)h_{c_1}\big). \end{aligned} \qquad (S14)$$

And the covariance of n_{c_1} and n_{c_2} is given by:

$$\begin{aligned} Cov\big(n_{c_1,i}, n_{c_2,i}\big) = {} & Var(g_i)d_{c_1,s}d_{c_2,s} + h_{c_1}h_{c_2}Var(a_i) + \big(h_{c_1}d_{c_2,s} + h_{c_2}d_{c_1,s}\big)Cov(g_i, a_i) \\ & + \delta_{c_1c_2}F_{c_1,s}\big(E(g_i)d_{c_1,s} + E(a_i)h_{c_1}\big). \end{aligned} \qquad (S15)$$

This equation may be understood as a matrix containing the covariance of the expected spike counts, $f_{c,i}$, plus an additional diagonal term corresponding to each neuron's private variability as produced by the spike generator.

To compute the Pearson correlation, we substitute the results of Eqs. S12 and S15 into the formula

$$\rho\big(n_{c_1,i}, n_{c_2,i}\big) = \frac{Cov\big(n_{c_1,i}, n_{c_2,i}\big)}{\sqrt{Var\big(n_{c_1,i}\big)Var\big(n_{c_2,i}\big)}}. \qquad (S16)$$

The correlation of any two units $\rho\big(n_{c_1,i}, n_{c_2,i}\big)$ can therefore be computed analytically in terms of the parameters $d_{c,s}$ and h_c and the statistical quantities $E(g_i), Var(g_i), E(a_i), Var(a_i),$ and $Cov(g_i, a_i)$. Examples of predictions of this analytical model are depicted in Figure S7.

Decoding

To estimate how d^2 depended on the fluctuations of additive offset and multiplicative gain, we constructed a homogeneous neural population with translation-invariant orientation-tuning curves (36 neurons whose preferred orientations are 5° apart). We took $d_{c,s}$ as a circular Gaussian function of width 15° and h_c as a constant value of 600 (both approximated from one example session of anesthetized cat data). The Fano-factor parameter $F_{c,s}$ for the spike count generator was fixed at 1.7 for all stimuli and cells. We investigated how d^2 depended on the values of $E(a_i)$, $E(g_i)$, $Var(a_i)$, and $Var(g_i)$ (within biologically plausible ranges estimated from anesthetized cat data) by using analytic formulae to evaluate

$\overline{\boldsymbol{n}}_1$, $\overline{\boldsymbol{n}}_2$, $Cov(\boldsymbol{n}_1)$, and $Cov(\boldsymbol{n}_2)$ (Supplemental Experimental Procedures: analytic calculation of pairwise correlations). The parameters were varied one at a time; parameters that were not changed were held fixed at $E(a_i)$ = 0.055, $E(g_i)$ = 0.3, $Var(a_i)$ = 0, and $Var(g_i)$ = 0.

Decoding analysis with reduced dimensionality

In order to gain an intuitive understanding of the population coding results (Figures 7 and 8), it is helpful to project the high-dimensional responses into a 1- or 2-dimensional space. Specifically, given the spike count vector $\boldsymbol{n_i}$ on trial i, we can project it onto a low-dimensional space by matrix multiplication:

$$\boldsymbol{\alpha_i} = B\boldsymbol{n_i}. \qquad (S17)$$

The models of population tuning curves shown in Figures 1 and 3 are examples of such a low-dimensional projection, with B given by the pseudoinverse of the matrix whose columns are the basis vectors $\boldsymbol{G}(\theta)$, corresponding to single-grating responses, and a column of ones that corresponds to the additive offset.

For the reduced-dimensional projection to faithfully represent discriminability in the high-dimensional space, two conditions are necessary. First, cluster separation in the low-dimensional space should be approximately equal to that in the full space. Discriminability in the low-dimensional space is given by

$$d'^2 = (B(\overline{\boldsymbol{n}}_2 - \overline{\boldsymbol{n}}_1))^{\mathrm{T}}\left(B\Sigma B^{\mathrm{T}}\right)^{-1}\left(B(\overline{\boldsymbol{n}}_2 - \overline{\boldsymbol{n}}_1)\right). \qquad (S18)$$

$\overline{\boldsymbol{n}}_s$ is a vector containing the mean spike count of all units over all presentations of stimulus s, and Σ is the population covariance matrix averaged across the two stimuli to be discriminated. Note that if B were invertible, d'^2 would equal d^2. For low-dimensional projections, B is a non-invertible rectangular matrix. Nevertheless, for the projections used in Figures 7 and 8, d'^2 is a close approximate of d^2 (Figures S8N and S8O). The second criterion is that the projection is orthogonal. The projections of Figures 1 and 3 are not orthogonal: although the responses to two gratings 90° apart are close to orthogonal, each grating response is not orthogonal to the constant offset. When projecting onto basis vectors corresponding to gratings of similar orientation, this non-orthogonality becomes extreme, as the two grating responses are very similar. To visualize responses in this space (Figure 8), we therefore used the Gram-Schmidt process to orthonormalize the single-grating responses.

The extended multiplicative model

Variable coupling to population activity is included in the additive model, but not in the multiplicative model. To investigate whether this was the sole reason for the affine model's better performance, we introduce an "extended multiplicative model" that allows each neuron to be coupled to the population activity to different degrees (Figure S2A), and we ask if this additional coupling flexibility in the extended multiplicative model better explains the shared variability than the additive component in the affine model.

In this model, the expected spike count $f_{c,i}$ of unit c on trial i is given by

$$f_{c,i} = (1 - K_c)d_{c,s(i)} + g_i K_c d_{c,s(i)}. \qquad (S19)$$

As before, $s(i)$ is the stimulus presented on trial i, $d_{c,s(i)}$ is the sensory drive to unit c from stimulus $s(i)$, g_i is the shared multiplicative gain on trial i, and the new term K_c represents the degree to which each unit c is coupled to this gain. For a unit with K_c = 0, its spiking activity is independent of its surrounding neuronal population, and the model reduces to the independent model. For a unit with K_c = 1, $d_{c,s(i)}$ is perfectly synched with the population activity, and its behavior can be described by the original

multiplicative model. g_i varies from trial to trial, whereas $d_{c,s(i)}$ and K_c take the same values on all trials. The number of parameters in this model ($M_{\text{units}} M_{\text{stimuli}} + M_{\text{units}} + M_{\text{trials}}$) is equivalent to the additive model.

To fit the model numerically, we first re-parametrized. Setting $k_c = \frac{1-K_c}{K_c}$ and $d'_{c,s(i)} = K_c d_{c,s(i)}$, we can rewrite Eq. S19 as

$$f_{c,i} = k_c d'_{c,s(i)} + g_i d'_{c,s(i)}, \tag{S20}$$

which in matrix form is

$$\begin{pmatrix} f_{c_1,1} \\ f_{c_1,2} \\ \vdots \\ f_{c1,M_{\text{trials}}} \\ f_{c_2,1} \\ f_{c_2,2} \\ \vdots \\ f_{c_2,M_{\text{trials}}} \\ \vdots \\ f_{c_{M_{\text{units}}},1} \\ f_{c_{M_{\text{units}}},2} \\ \vdots \\ f_{c_{M_{\text{units}}},M_{\text{trials}}} \end{pmatrix} = \begin{pmatrix} d'_{c_1,s(1)} & 0 & \cdots & 0 & d'_{c_1,s(1)} & 0 & \cdots & 0 \\ d'_{c_1,s(2)} & 0 & \cdots & 0 & 0 & d'_{c_1,s(2)} & \cdots & 0 \\ \vdots & \vdots & \vdots & \vdots & \vdots & \vdots & \vdots & \vdots \\ d'_{c_1,s(M_{\text{trials}})} & 0 & \cdots & 0 & 0 & 0 & \cdots & d'_{c_1,s(M_{\text{trials}})} \\ 0 & d'_{c_2,s(1)} & \cdots & 0 & d'_{c_2,s(1)} & 0 & \cdots & 0 \\ 0 & d'_{c_2,s(2)} & \cdots & 0 & 0 & d'_{c_2,s(2)} & \cdots & 0 \\ \vdots & \vdots & \vdots & \vdots & \vdots & \vdots & \vdots & \vdots \\ 0 & d'_{c_2,s(M_{\text{trials}})} & \cdots & 0 & 0 & 0 & \cdots & d'_{c_2,s(M_{\text{trials}})} \\ \vdots & \vdots & \vdots & \vdots & \vdots & \vdots & \vdots & \vdots \\ 0 & 0 & \cdots & d'_{c_{M_{\text{units}}},s(1)} & d'_{c_{M_{\text{units}}},s(1)} & 0 & \cdots & 0 \\ 0 & 0 & \cdots & d'_{c_{M_{\text{units}}},s(2)} & 0 & d'_{c_{M_{\text{units}}},s(2)} & \cdots & 0 \\ \vdots & \vdots & \vdots & \vdots & \vdots & \vdots & \vdots & \vdots \\ 0 & 0 & \cdots & d'_{c_{M_{\text{units}}},s(M_{\text{trials}})} & 0 & 0 & \cdots & d'_{c_{M_{\text{units}}},s(M_{\text{trials}})} \end{pmatrix} \begin{pmatrix} k_{c_1} \\ k_{c_2} \\ \vdots \\ k_{c_{M_{\text{units}}}} \\ g_1 \\ g_2 \\ \vdots \\ g_{M_{\text{trials}}} \end{pmatrix}.$$

This allows us to fit the extended multiplicative model to experimental data by an alternation method: given a current estimate of $d'_{c,s(i)}$, k_c and g_i are estimated via ridge regression with $\sqrt{\lambda} = 0.02$; then the scaled sensory drive term $d'_{c,s(i)}$ is again fit via ridge regression with $\sqrt{\lambda} = 0.02$. The alternation is repeated until a convergence criterion is met, specifically that the difference in squared error per unit per trial $\frac{\sum_{c,i}(f_{c,i} - N_{c,i})^2}{M_{\text{units}} \times M_{\text{trials}}}$ between two iterations was less than 10^{-10}. This typically took few thousand iterations.

In all sessions of anesthetized cat recording, the extended multiplicative model outperformed both the additive model (Figure S2B & Table S2; $p < 10^{-24}$ for all data together and $p < 0.007$ in all recording sessions evaluated individually; sign test) and the multiplicative model (Figure S2C & Table S2; $p < 10^{-15}$ for all data together, $p < 0.008$ in 5 individual sessions and $p < 0.03$ in 1 session; sign test). Nevertheless, the affine model bested the extended multiplicative model (Figure S2D & Table S2; $p < 10^{-22}$ for all data together, $p < 10^{-4}$ in 4 individual sessions and $p < 0.05$ in 2 others; sign test). In the quietly awake mouse data, the extended multiplicative model sometimes performed better than the additive model (Figure S2E & Table S4; $p < 0.03$ for all data together, $p < 0.02$ in 3 individual sessions, out of which one was better predicted by the additive model; sign test) and the multiplicative model (Figure S2F & Table S4; $p < 0.004$ for all data together and $p < 0.02$ for 2 individual sessions; sign test). Yet again, the affine model performed considerably better than the extended multiplicative model (Figure S2G & Table S4; $p < 10^{-4}$ for all data together, $p < 0.02$ in 3 individual sessions; sign test). In addition, the extended multiplicative model – as the original multiplicative model - failed to capture all possible forms of the correlation matrix: the extended multiplicative model also tended to overestimate the edge-bin correlations while under-estimating the corner-bin correlations (Figures S2H-S2O). These results suggest that the additive com-ponent in the affine model could not be trivially explained away by each cell's coupling strength. While including a cell-coupling factor for the multiplicative gain in the full affine model might potentially improve

the model even further, estimating this model would present severe numerical difficulties, and it is outside the scope of present work.

Supplemental References

Harris, K.D., Hirase, H., Leinekugel, X., Henze, D.A., and Buzsaki, G. (2001). Temporal interaction between single spikes and complex spike bursts in hippocampal pyramidal cells. Neuron 32, 141-149.

Schmitzer-Torbert, N., Jackson, J., Henze, D., Harris, K., and Redish, A.D. (2005). Quantitative measures of cluster quality for use in extracellular recordings. Neuroscience 131, 1-11.
