# Supplementary material for: The Nature of Shared Cortical Variability
Source: Neuron. 2015 Aug 5;87(3):644–56. doi: 10.1016/j.neuron.2015.06.035 (PMC4534383; doi:10.1016/j.neuron.2015.06.035)
Supplement: Document S2. Article plus Supplemental Information [file mmc2.pdf]

Article

Neuron

The Nature of Shared Cortical Variability

Highlights

- Response variability in V1 neuronal populations is largely shared across neurons
- Shared variability involves two factors: a multiplicative gain and an additive offset
- These two factors predict sensory responses of large populations on single trials
- They determine pairwise correlations and constrain information coding

Authors

I-Chun Lin, Michael Okun, Matteo Carandini, Kenneth D. Harris

Correspondence

i.lin@ucl.ac.uk (I.-C.L.),
kenneth.harris@ucl.ac.uk (K.D.H.)

In Brief

Cortical responses are highly variable. Using large-scale recordings in V1, Lin et al. show that this variability is shared across neurons and involves two simple factors: multiplicative and additive. These factors shape the joint variability of large populations of neurons.

Lin et al., 2015, Neuron *87*, 644–656
August 5, 2015 ©2015 The Authors
http://dx.doi.org/10.1016/j.neuron.2015.06.035

Neuron
Article

The Nature of Shared Cortical Variability

I-Chun Lin,[1,2,3,*] Michael Okun,[1,2,3] Matteo Carandini,[2,4] and Kenneth D. Harris[1,3,4,*]
[1]UCL Institute of Neurology, University College London, London WC1N 3BG, UK
[2]UCL Institute of Ophthalmology, University College London, London EC1V 9EL, UK
[3]UCL Department of Neuroscience, Physiology, and Pharmacology, University College London, London WC1E 6DE, UK
[4]Co-senior author
*Correspondence: i.lin@ucl.ac.uk (I.-C.L.), kenneth.harris@ucl.ac.uk (K.D.H.)
http://dx.doi.org/10.1016/j.neuron.2015.06.035
This is an open access article under the CC BY license (http://creativecommons.org/licenses/by/4.0/).

SUMMARY

Neuronal responses of sensory cortex are highly variable, and this variability is correlated across neurons. To assess how variability reflects factors shared across a neuronal population, we analyzed the activity of many simultaneously recorded neurons in visual cortex. We developed a simple model that comprises two sources of shared variability: a multiplicative gain, which uniformly scales each neuron's sensory drive, and an additive offset, which affects different neurons to different degrees. This model captured the variability of spike counts and reproduced the dependence of pairwise correlations on neuronal tuning and stimulus orientation. The relative contributions of the additive and multiplicative fluctuations could vary over time and had marked impact on population coding. These observations indicate that shared variability of neuronal populations in sensory cortex can be largely explained by two factors that modulate the whole population.

INTRODUCTION

Repeated presentations of the same stimulus elicit highly variable responses in sensory cortex (Heggelund and Albus, 1978; Tolhurst et al., 1983; Vogels et al., 1989). This variability is correlated across neurons, so it cannot be easily removed by averaging across the population, and may thus place critical constraints on information transmission (Averbeck et al., 2006; Averbeck and Lee, 2006; Deweese and Zador, 2004; Shadlen and Newsome, 1998; Zohary et al., 1994). Understanding its nature can thus shed light on the circuit mechanisms and computations performed by the cortex in health and disease (Dinstein et al., 2015).

Cortical variability does not arise because neurons are intrinsically noisy. Indeed, cortical neurons can generate highly reliable spike trains (Mainen and Sejnowski, 1995). The variability of their responses is therefore more likely to arise from the variability of their synaptic inputs, reflecting cortical network dynamics (Carandini, 2004). Traditionally, variability has been studied in single neurons or neuronal pairs, but a full description requires understanding factors operating at the population level.

A clue toward understanding cortical variability comes from spontaneous activity patterns that the cortex produces in the absence of stimuli. These patterns share some features with responses evoked by sensory stimuli (Arieli et al., 1996; Kenet et al., 2003; Luczak et al., 2009, 2013; Ringach, 2009; Tsodyks et al., 1999): for instance, pairwise correlations measured during spontaneous activity can resemble those seen during sensory stimulation (Jermakowicz et al., 2009; Okun et al., 2012). Voltage-sensitive dye imaging experiments in visual cortex suggest that the interaction between spontaneous and evoked activity is additive: activity would be the sum of a deterministic sensory response and a stochastic pattern originating from networks that generate spontaneous activity (Arieli et al., 1996). Subsequent work showed that such an additive interaction could approximate the dependence of pairwise correlations on cortical state (Schölvinck et al., 2015). Other work suggests a more complex picture. In auditory cortex, responses to prolonged tone stimuli show similar fluctuating activity to those seen in silence (Luczak et al., 2013). Yet, sensory-evoked spikes do not occur independently of these fluctuations, as would be expected from addition, but occur together with them, suggesting that spontaneous fluctuations gate the representation of stimuli. In visual cortex, quantitative analyses suggest that the variability of single neurons and correlations of neuronal pairs are more consistent with a multiplicative gain change, whose gain factor fluctuates from trial to trial (Ecker et al., 2014; Goris et al., 2014).

Such multiplicative variability is consistent with what one might expect from top-down feedback from higher order cortices. By targeting layer 1, this feedback can change the gain with which neurons respond to activity in input layers (Larkum, 2013; Larkum et al., 1999). Top-down feedback might be involved in spatial attention (Armstrong and Moore, 2007; Moore and Armstrong, 2003), which can have a multiplicative effect on neuronal gain (Reynolds and Heeger, 2009). Yet, other evidence suggests that the influence of spatial attention is additive (Boynton, 2009; Buracas and Boynton, 2007; Murray, 2008; Thiele et al., 2009). Other modulatory effects, such as those seen in visual cortex during locomotion, appear to be both additive and multiplicative (Ayaz et al., 2013).

These observations raise multiple questions. If single-neuron variability is well-modeled by multiplicative gain changes, can a single, population-wide gain factor explain the coordinated fluctuations of the population? Are the additive and multiplicative models mutually exclusive or is there a common ground between

CellPress

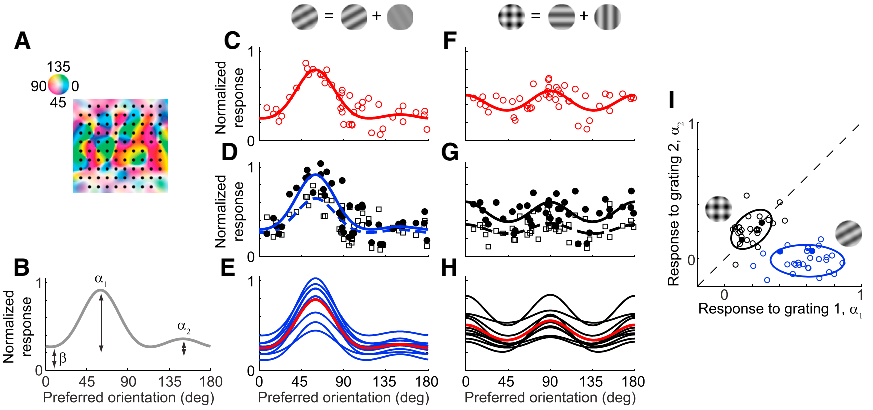

Figure 1. Recordings of Population Activity in Anesthetized Cat V1

(A) Layout of a 10-by-10 electrode array aligned to the underlying map of preferred orientations (adapted from Katzner et al., 2009). The electrode sites are 400 μm apart.

(B) The function fitted to population responses is the sum of two circular Gaussians: one peaking at the orientation of grating 1 with amplitude α_1, and one peaking at the orientation of grating 2 with amplitude α_2. A baseline untuned response β provides an additive offset.

(C) Population response averaged over ten presentations of a plaid with orientations 60° and 150° and contrasts 50% and 6%. Each circle shows the trial-averaged normalized firing rate of an orientation-tuned site, arranged by its preferred orientation. The firing rate is normalized by each site's response to its optimal orientation at 100% contrast. The curve indicates the fit of the function in (B).

(D) Two example single-trial population responses to the plaid shown in (C). A filled circle and an empty square represent the normalized firing rates of an orientation-tuned site on trials 1 and 2; the solid and dashed lines plot the fits of the function in (B) to population responses on trials 1 and 2.

(E) Population responses to ten presentations of the plaid shown in (C) (blue); only fitted curves are shown for clarity. A red curve repeats the trial-averaged population response for comparison.

(F–H) As in (C)–(E), but for a plaid with component gratings of orientations 0° and 90°, both at 50% contrast.

(I) Variability in population responses measured by their response to grating 1, α_1, and their response to grating 2, α_2. The blue circles indicate responses to any plaids with contrasts 50% (grating 1) and 6% (grating 2); the black circles indicate responses to any plaids with contrasts 50% and 50%. Each open circle denotes population response on one trial; the four solid circles mark the four single-trial population responses shown in (D) and (G). For each component-contrast combination, the data were pooled across different component orientations. The ellipses show 1 SD contours of Gaussian fits (session 83-7-5, 45 orientation-tuned sites, plaid angle = 90°).

them? And what are the effects of the multiplicative and additive fluctuations on the cortical code?

Here, we answer these questions by analyzing the trial-by-trial activity of large, simultaneously recorded neural populations in primary visual cortex (V1) of anesthetized cats and quietly awake mice. We find that cortical variability is best understood at the population level, where it can be described by a simple mathematical model comprising two sources of shared variability: multiplicative and additive. This model explained the structure of trial-to-trial population variability and captured the complex dependence of neuronal correlations on stimulus and neuronal tuning. Our results suggest that neither additive nor multiplicative variability alone forms a complete model of cortical variability; instead, a combination of the additive and multiplicative components that invest the whole population can explain much of the shared response variability in visual cortex.

RESULTS

We first analyzed the activity of large neural populations in V1 of anesthetized cats (seven recording sessions from three neuronal populations in three cats). Neuronal responses were recorded from a 10-by-10 electrode array that covered a 16 mm^2-region with a diversity of orientation preferences (Figure 1A). All spikes detected on a given site of the array were pooled, as they originated from neurons having similar preferred orientations (Katzner et al., 2009). Stimuli were contrast-reversing oriented gratings and plaids consisting of two superimposed component gratings. These data sets were previously analyzed after averaging across trials of the same stimulus (Busse et al., 2009); here, we examined them in individual trials.

Variability Is Shared across the Population

Repeated presentations of the same stimulus elicited highly variable responses, yet this variability was coordinated across the population. To illustrate the nature of this variability, we plotted "population tuning curves", a graphical summary of the population response to a plaid stimulus (Figure 1B). As expected, a plaid with very different component contrasts evoked the largest mean activity at sites tuned for the orientation of the high-contrast component grating (Figure 1C), a form of winner-take-all competition (Busse et al., 2009). Yet, the responses of all tuned sites varied from trial to trial; for instance, firing rate tended to be higher on one trial than on another, and this difference affected most sites simultaneously (Figure 1D). As a result, the curves fitted to the population activity changed noticeably from trial to trial (Figure 1E). Similar results were obtained for different stimuli. For instance, as observed previously (Busse et al., 2009), a plaid with equal component contrasts elicited large mean responses at sites tuned for either of the component orientations (Figure 1F). Trial-by-trial responses were again highly variable, and the variability seemed to be coordinated at the population level (Figures 1G and 1H).

Although trial-by-trial variability was clearly coordinated across the population, the nature of this shared variability was not immediately obvious. In the first example, population responses seemed to be scaled multiplicatively between trials (Figure 1D). Yet, in the second example, population responses seemed to be shifted by a common offset between trials (Figure 1G). There were also examples that spoke in favor of a mixture of additive and multiplicative effects (Figures 1E and 1H).

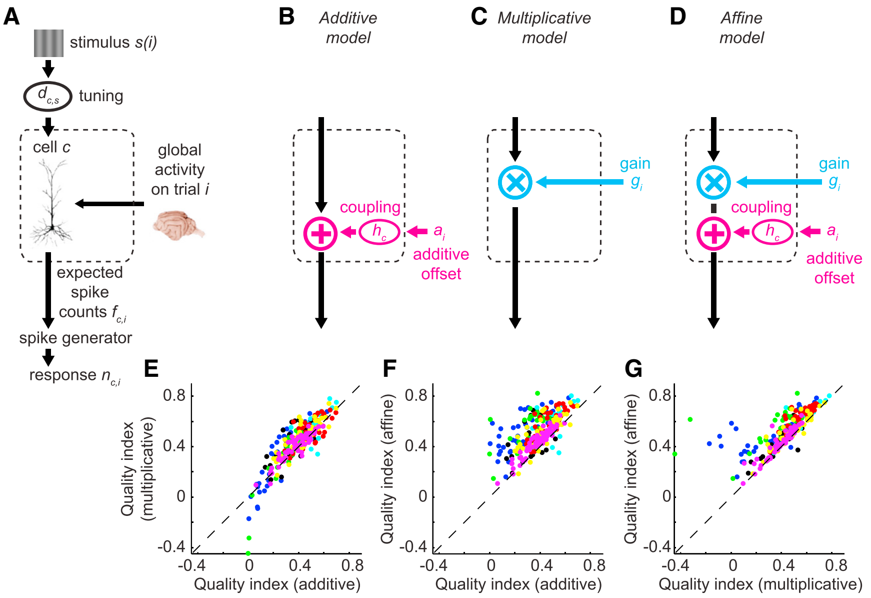

Figure 2. The Additive, Multiplicative, and Affine Models

(A) The basic structure of the models involves a unit c, whose expected spike count $f_{c,i}$ on trial i depends on both the tuning $d_{c,s}$ of that unit for stimulus $s(i)$ shown on that trial and global, shared factors that originate from the rest of the brain and vary from trial to trial. The expected spike count $f_{c,i}$ is then passed into a stochastic spike count generator that generates private variability, yielding an integer spike count $n_{c,i}$ from a negative binomial distribution with mean $f_{c,i}$ and cell- and stimulus-dependent Fano factor $F_{c,s}$.

(B) In the additive model, the global factor is the additive offset a_i that affects each unit c by an amount proportional to a coupling term h_c.

(C) In the multiplicative model, the global factor is the variable response gain g_i that uniformly scales the sensory drive $d_{c,s}$.

(D) The affine model includes both the additive and multiplicative components.

(E–G) Cross-validated performance of the response $n_{c,i}$ generated by the multiplicative model versus the additive model (E) and by the affine model versus the additive (F) and multiplicative (G) models across seven sessions in three cats. The performance was measured by the quality index, which is zero or negative if the prediction is not better than the independent model and equals 1 for a perfect prediction. Each circle represents the performance on one site across all trials in a session; sites from the same session share the same color. Only sites that had quality index > 0.1 for at least one of the models were shown.

To explore the structure of this variability and to gain an intuition into how it is shared between neurons, we characterized the population response on each trial as a single, compact population tuning curve determined by three free parameters. The population tuning curve $\boldsymbol{R}_i$ (a vector whose values are the normalized firing rates of all orientation-tuned sites, ordered by their preferred orientations) on trial i was fit as a linear combination of prototypical responses to the two component gratings of a plaid, plus a constant shift (Figure 1B):

$$\boldsymbol{R}_i = \alpha_1^i \boldsymbol{G}\left(\theta_1^i\right) + \alpha_2^i \boldsymbol{G}\left(\theta_2^i\right) + \beta^i. \quad \text{(Equation 1)}$$

Here, θ_1^i and θ_2^i are the component orientations of the plaid stimulus presented on trial i, and $\boldsymbol{G}(\theta)$ is the prototypical response (a circular Gaussian) to a grating of orientation θ. The population tuning curve $\boldsymbol{R}_i$ on each trial i is thus determined by three parameters: the tuned response to component grating 1, α_1^i, the tuned response to component grating 2, α_2^i, and a baseline untuned response, β^i. These parameters were fit by least-squares to the population response; they effectively summarized the population activity of many tens of sites on each trial, accounting for 47 ± 18% (median ± median absolute deviation) of the variance. We used these fits to examine the population data and the predictions of various models of neuronal variability (but not to fit the models, which was done on the actual spike counts as a function of site and time, as described below).

The parameters of the population tuning curves confirmed that the trial-to-trial variability included a shared multiplicative component (Figure 1I). For a plaid with equal component contrasts, the tuned-response components to gratings 1 and 2, α_1^i and α_2^i, were positively correlated across trials: the ellipse summarizing their distribution was clearly diagonal (Figure 1I, *black; r = 0.45*). By comparison, for a plaid with markedly different component contrasts, the tuned response α_2^i to the low-contrast grating stayed close to zero regardless of the tuned response α_1^i to the high-contrast grating (Figure 1I, *blue*; $r = -0.05$). These two elongated clouds radiating outward from the origin are what would be expected from shared multiplicative variability. They do not, however, rule out the presence of additive variability, which causes variations in the parameter β^i (Figure S3).

The Affine Model

The shared nature of trial-to-trial variability described above suggests that trial-to-trial fluctuations in population activity might be accountable by a small number of factors, whose joint effect on each neuron can be multiplicative and/or additive. To formalize this idea, we turned to a more rigorous approach that seeks to predict the response of every site to every stimulus on every trial. We developed a set of models that operate at the level of spike trains of units (single neuron or multiunit) in a population (not at the level of the summary statistics α_1, α_2, and β previously described). In these models, the response of each unit on a trial depends on both the tuning of that unit to the stimulus shown on that trial and a trial-varying, global, shared factor that originates from the rest of the brain (Figure 2A). To account for the different factors of shared variability, we considered an additive model (Figure 2B), a multiplicative model (Figure 2C), and an affine model (Figure 2D), which encompasses them both.

In the affine model, the expected spike count of unit c on trial i, during which stimulus $s(i)$ is presented, is

$$f_{c,i} = g_i d_{c,s(i)} + a_i h_c, \quad \text{(Equation 2)}$$

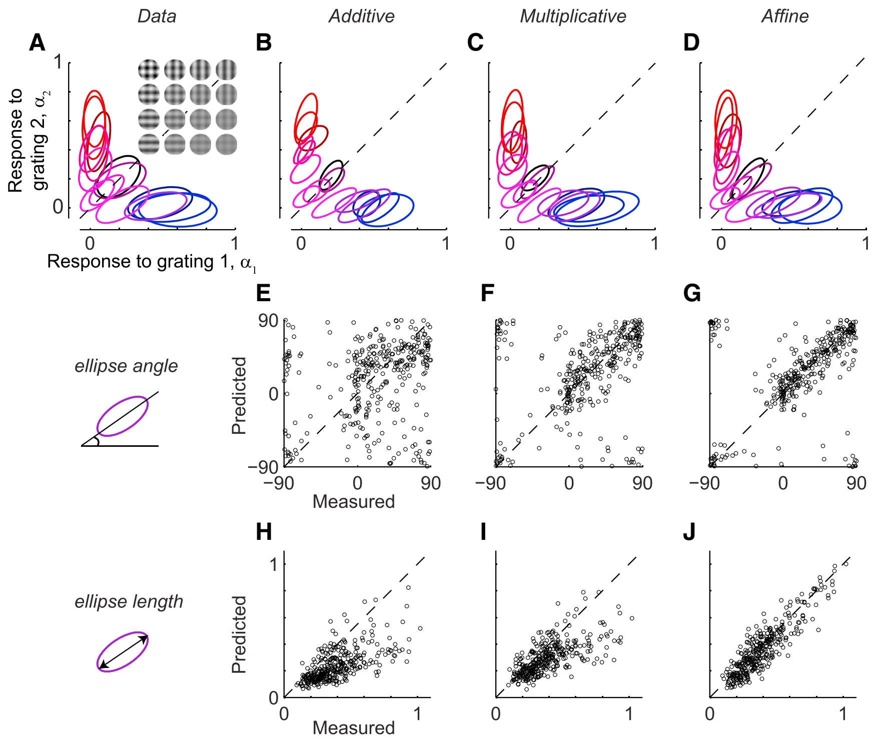

Figure 3. Variability of Population Response to Plaid Stimuli

(A) Ellipses showing 1 SD contours of Gaussian fits to the distributions of population responses to gratings 1 and 2 (α_1 and α_2) for an example session. The 16 ellipses correspond to 16 plaid stimuli in which the contrasts of the two component gratings are varied independently. The colors indicate the component contrasts (RGB color code with red encoding the contrast of grating 1 and blue encoding the contrast of grating 2). For each component-contrast combination, the data were pooled across different component orientations. Two of the ellipses appeared in Figure 1I (session 83-7-5, 45 orientation-tuned sites, plaid angle = 90°).

(B–D) Ellipses fitted to responses simulated by the additive (B), multiplicative (C), and affine (D) models.

(E–G) Comparison of the ellipse angles generated by the three models versus experimental data across seven sessions in three cats. Each plaid stimulus in each session contributes a dot for the ellipse fitted to the distribution of α_1 versus α_2.

(H–J) Same as (E)–(G), but for the comparison of the major-axis lengths of the ellipses.

where $d_{c,s(i)}$ is the deterministic sensory drive to unit c arising from stimulus $s(i)$. This term reflects the unit's sensory tuning as well as its contextual interactions; i.e., anything that contributes to the unit's mean response to that stimulus; e.g., divisive normalization (Busse et al., 2009). The multiplicative gain g_i scales the firing rate of all units in proportion to their sensory drive; i.e., it controls their response gain. The additive offset a_i adds to their firing rates in proportion to their coupling factors h_c. Constraining $g_i = 1$ gives a purely additive model (Figure 2B); setting $a_i = 0$ results in a purely multiplicative model (Figure 2C). Constraining both $g_i = 1$ and $a_i = 0$ gives a model that takes no shared variability into account (referred as the independent model; Figure S4A).

To compare model predictions with recorded spike counts, we passed $f_{c,i}$ into a stochastic spike count generator, which yields an integer spike count $n_{c,i}$ from a negative binomial distribution with mean $f_{c,i}$ and cell- and stimulus-dependent Fano factor $F_{c,s(i)}$, estimated by maximum likelihood. This stochastic spike count generator delivers the fraction of variability that is not shared, but private to each unit (Deweese and Zador, 2004).

Population Variability Is Both Multiplicative and Additive

To study how well these global, shared factors could explain cortical variability, we fit the affine model and compared the results with the purely additive and multiplicative models. We obtained the model parameters by fitting the model predictions $f_{c,i}$ to the spike counts recorded at each site, and we evaluated the fits with cross-validation (see Supplemental Information: cross-validation; Figure S1). Performance was measured by the quality index q: the improvement in cross-validated prediction compared to the independent model with no shared variability. The quality index is zero or negative for a model that offers no improvement over the independent model, and equals 1 for a perfect prediction.

This analysis showed that the affine model is superior to both the additive and multiplicative models (Figures 2E–2G). The multiplicative model performed better than the additive model (Figure 2E; Table S1; $p < 10^{-11}$ for all data together, $p < 0.03$ in five out of seven recording sessions evaluated individually; sign test). In all sessions, the affine model considerably outperformed both the additive model (Figure 2F; Table S1; $p < 10^{-43}$ for all data together, $p < 10^{-6}$ in five individual sessions, and $p < 0.02$ in the remaining two; sign test) and the multiplicative model (Figure 2G; Table S1; $p < 10^{-43}$ for all data together, $p < 10^{-8}$ in four individual sessions, and $p < 0.001$ for the rest; sign test). Because these results were cross-validated, the affine model could not gain a numerical advantage by over-fitting. Rather, the results indicate that neither the additive nor multiplicative factors alone suffice: combining the two forms a better model for shared cortical variability.

Note that the additive model—unlike the multiplicative model—has a coupling term h_c that allows each unit to be coupled to population activity differently, a phenomenon that has been previously described (Okun et al., 2015). Introducing a similar cell-coupling term to the multiplicative model did improve its performance, but this extended multiplicative model still performed worse than the affine model (Figures S2A–S2D; Table S2; Supplemental Information: the extended multiplicative model). The superior performance of the affine model thus reflects the presence of an additive component rather than simply its ability to model different coupling strengths of different neurons.

To gain further intuition into the performance of the models, we returned to the reduced representation of the population activity (Figure 3). We first computed the population tuning curves for the recorded and simulated activities. We then fit ellipses to

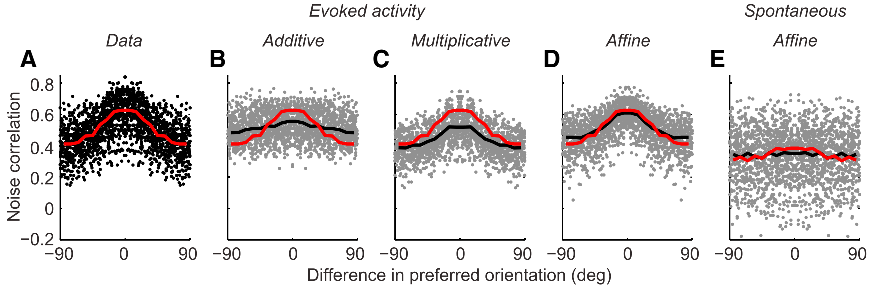

Figure 4. Relationship between Noise Correlation and Tuning Similarity

(A) Noise correlation for each pair of orientation-tuned sites in response to single gratings, as a function of the difference between their preferred orientations. A black dot represents noise correlation calculated for a pair of sites, and the red curve shows the running median.

(B–D) As in (A), but for predictions of the additive (B), multiplicative (C), and affine (D) models. A gray dot represents noise correlation predicted by the model for a pair of sites, a black curve shows the running median, and a red curve repeats the running median of the measured data for comparison.

(E) As in (D), but for spontaneous correlations. Note that even for spontaneous activity, noise correlations were higher for similarly tuned sites, but this could not be captured by the model. All running medians were calculated with non-overlapping 10° bins (session 83-7-5, 45 orientation-tuned sites).

summarize their variability (as in Figure 1I) and examined the ellipses corresponding to all plaid stimuli (Figures 3, S3, and S4). In the data, the tuned responses to component gratings 1 and 2 tended to be correlated only for plaids with equal component contrasts, resulting in ellipses emanating from the origin (Figure 3A). The additive model produced tuned responses that were slanted (i.e., highly correlated) for all plaid stimuli, including those with unequal component contrasts (Figure 3B). The multiplicative and affine models, by contrast, captured the angle of the ellipses appropriately (Figures 3C and 3D). Indeed, the affine model outperformed both the additive and multiplicative models in predicting the ellipse angles across all seven sessions (Figures 3E–3G). The affine model also provided better predictions of ellipse lengths, which reflect the strength of correlated variability (Figures 3H–3J). Analyzing correlations between the tuned responses and the baseline, untuned activity gave similar conclusion (Figure S3). Finally, all three models outperformed the independent model (Figure S4).

In short, the affine model was superior in capturing correlations between the two tuned-response components as well as correlations between the tuned- and untuned-response components. The affine model thus explains much of the response variability and surpasses both the additive and multiplicative models in predicting not only the raw individual spike counts (Figure 2), but also our summary analysis of ensemble activity, the population tuning curves (Figures 3, S3, and S4).

Dependence of Noise Correlations on Tuning Similarity

Our results suggest that the correlated response variability of neuronal populations can be well modeled by only two global factors, additive and multiplicative, that are shared across the population. In a population of N neurons, there are order N^2 pairwise correlations. To what extent can these pairwise correlations be explained by these two factors?

To address this, we measured pairwise noise correlations for all pairs of orientation-tuned sites in response to single-grating stimuli. Correlations tended to be high because they were measured over long time windows and involved multiunit activity (Cohen and Kohn, 2011). We found that noise correlations predicted by the affine model were closer to the measured values, compared to either the additive model (Figures S5A–S5U; $p = 10^{-48}$ for all data together, $p < 10^{-4}$ in four individual sessions, and $p < 0.05$ in one session; sign test on the squared errors between measured and predicted noise correlations) or the multiplicative model (Figures S5A–S5U; $p < 10^{-54}$ for all data together, $p < 10^{-5}$ in five individual sessions, and $p < 0.05$ in the remaining two; sign test).

We next asked whether the affine model could predict the well-known relationship between signal and noise correlations: noise correlations are larger between cells with similar sensory tuning (reviewed in Cohen and Kohn, 2011). This relationship is often attributed to increased connectivity between neurons with similar sensory tuning, but it can also arise from shared multiplicative variability (Brody, 1999; Ecker et al., 2014; Goris et al., 2014). This is simply because multiplication affects neurons that are responding to a stimulus more than neurons that are not responding, thus introducing correlations among neurons with similar tuning.

We measured the dependence of noise correlations on the difference in preferred orientations and compared it to the one calculated from spike counts simulated by the additive, multiplicative, and affine models (Figure 4). As expected, noise correlations showed a clear dependence on orientation-tuning similarity: sites preferring the same orientation were more strongly correlated (Figure 4A). This dependence could not be captured by the additive model, which predicted a negligible dependence on tuning similarity (Figure 4B). The multiplicative model predicted stronger noise correlations when preferred orientations were more similar, but correlations were generally underestimated (Figure 4C). The affine model almost completely accounted for the dependence of noise correlations on tuning similarity (Figure 4D); this superiority was statistically significant in all sessions ($p < 0.05$ for both additive versus affine and multiplicative versus affine; *t* test on each session's sum of squared errors between running medians of data and model; Figure S6).

Still, the affine model slightly underestimated the dependence of pairwise correlations on tuning difference (Figure 4D). To investigate this mild imperfection, we looked at the spontaneous correlations; i.e., the pairwise correlations measured in the absence of stimuli (Figure 4E). Spontaneous correlations have been reported to be strongest for similarly tuned cells (Jermakowicz et al., 2009; Kenet et al., 2003; Okun et al., 2015). Our data showed this effect, albeit weakly (Figure 4E, red curve). Unsurprisingly, none of the three models could predict this dependence; for instance, the predictions of the affine model were essentially flat (Figure 4E, black curve). This small,

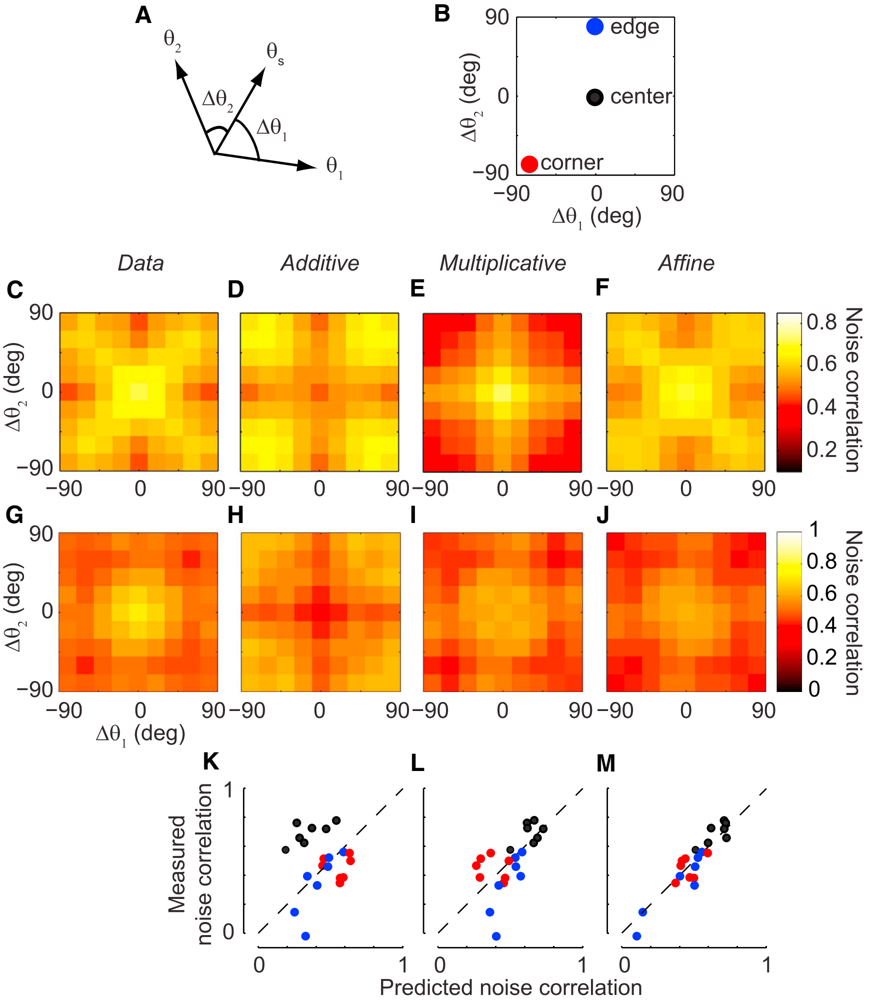

Figure 5. Dependence of Noise Correlations on Stimulus Orientation and Tuning Preferences

(A) Noise correlation for each pair of sites in response to a single grating was analyzed as a function of three parameters: the stimulus orientation, θ_s; the preferred orientation of site 1, θ_1; and the preferred orientation of site 2, θ_2. Assuming rotational symmetry, we could reduce these three parameters to two: the difference between the preferred orientations of the two sites, and the stimulus orientation, $\Delta\theta_c = \theta_c - \theta_s$, for c = 1 or 2.

(B) The dependence of noise correlations on stimulus orientation and tuning preferences are summarized by three numbers: the median noise correlations in the center bin (black circle, measuring the correlation produced when two co-tuned sites are stimulated with a grating of their preferred orientation), the corner bin (red circle, measuring the correlation produced when two co-tuned sites are stimulated with a grating whose orientation is orthogonal to their preferred orientation), and the edge bin (blue circle, measuring the correlation produced when two oppositely tuned sites are stimulated with a grating of one of their preferred orientations).

(C) Pseudocolor representation of median noise correlations for all pairs of orientation-tuned sites as a function of $\Delta\theta_1$ and $\Delta\theta_2$. The data were pooled across all contrasts and orientations (session 83-7-5, 45 orientation-tuned sites).

(D–F) As in (C), but for predictions of the additive (D), multiplicative (E), and affine (F) models.

(G–J) Same as (C)–(F), but for a different session in which the multiplicative model alone could reasonably reproduce the structure of the measured correlation matrix (session 83-10-15, 42 orientation-tuned sites).

(K–M) Scatter plots comparing the measured and predicted correlations for the additive (K), multiplicative (L), and affine (M) models across seven sessions in three cats. Each circle shows the measured and predicted noise correlations for one bin and one session, color-coded as in (B).

but noticeable, error in accounting for spontaneous correlations hints at what might be missing from the affine model: a slightly higher coupling between neurons that are similarly tuned.

To further understand the predictions of the three models, we considered an additional factor that determines noise correlations: the orientation θ_s of the stimulus relative to the preferred orientations θ_1 and θ_2 of the two sites. Noise correlations between a neuronal pair depend not only on the neurons' sensory tuning, but also on the attributes (orientation in this case) of the stimulus (Kohn and Smith, 2005; Ponce-Alvarez et al., 2013). Assuming rotational symmetry, this dependence on stimulus orientation and cell tuning can be summarized by a function $\rho(\Delta\theta_1, \Delta\theta_2)$, where $\Delta\theta_c = \theta_c - \theta_s$ is the preferred orientation of site c relative to stimulus orientation θ_s (Figure 5A). This representation was particularly informative in three locations: (1) the "center" bin, where the preferred orientations of both sites match the stimulus orientation; (2) the "edge" bin, where one site's tuning matches the stimulus and the other is orthogonal to it; and (3) the "corner" bin, where the preferred orientations of both sites are orthogonal to the stimulus orientation (Figure 5B).

This representation revealed a rich structure of pairwise correlations (Figures 5C–5J). In a typical session, noise correlations peaked in the center bin (Figure 5C); this observation was well predicted by the multiplicative and affine models (Figures 5E and 5F), but not by the additive model (Figure 5D). As has been previously observed (Cotton et al., 2013), correlations between similarly tuned sites were also high in the corner bins; this was predicted by the additive and affine models (Figures 5D and 5F), but not by the multiplicative model (Figure 5E). Finally, the data showed the lowest correlations in the edge bins; this was predicted by the affine and additive models (Figures 5D and 5F), but not by the multiplicative model (Figure 5E). While this particular structure of the correlation matrix was the most common (four out of seven sessions), it was not the only one we observed. Other recordings showed a different correlation structure that, again, was well captured by the affine model (e.g., Figures 5G–5J).

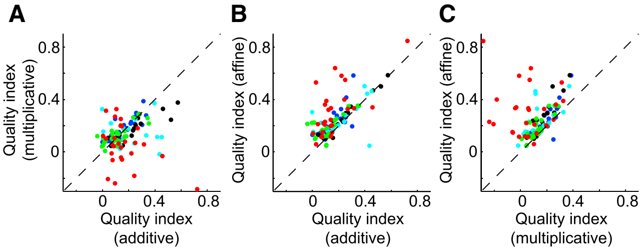

Figure 6. Performance of the Additive, Multiplicative, and Affine Models in Single-Unit Data from Quietly Awake Mice

(A–C) Cross-validated performance of the multiplicative model versus the additive model (A) and of the affine model versus the additive (B) and multiplicative (C) models (cf. Figures 2E–2G). Each circle represents the performance on one neuron across all trials in a session; neurons from the same session share the same color (five recording sessions in four mice). Only neurons that had a quality index > 0.1 for at least one of the models were shown.

To assess each model's performance across sessions, we computed the median noise correlations that fell into the center, corner, and edge bins of the correlation matrix (Figures 5K–5M). As in previous examples, the additive model tended to underestimate correlations in the center bin (Figure 5K). The multiplicative model did better (Figure 5L), but not as well as the affine model (Figure 5M). Note that the correlation matrices predicted by the extended multiplicative model were similar to the multiplicative model (Figures S2H–S2O). Only the affine model could predict all possible structures of the correlation matrices. In fact, the rich structure of these correlation matrices can be analytically predicted by the affine model (see Supplemental Information: analytic calculation of pairwise correlations; Figure S7). We conclude that the affine model concisely accounts for the complex, session-dependent relationship of noise correlations to neuronal tuning and stimulus.

Shared Cortical Variability in Quietly Awake Mice Is Both Multiplicative and Additive

We next asked whether the affine model—derived from anesthetized cat data—is also a good description of shared variability in the unanesthetized cortex. To this end, we used multisite silicon probes to record population activity in V1 of quietly awake, head-fixed mice (five sessions in five neuronal populations in four mice) in response to drifting gratings (12 directions at 100% or 60% contrast). The data were spike-sorted and only stable, well-isolated single units were used for further analysis. As expected (Busse et al., 2009; Niell and Stryker, 2008), the mean firing rates were substantially lower for single neurons in mouse V1 than for multiunit activity in cat V1 (mean of 2.8 versus 30.3 spikes/s). Even with such low firing rates, a majority (58%) of neurons showed detectable shared variability (quality index $q > 0.1$ for at least one of the models; Table S3). We focused on these neurons, fit the models, and asked which model better described this shared variability.

The results demonstrate that as in anesthetized cats, shared cortical variability in awake mice could be described by a combination of additive and multiplicative components (Figure 6). Differences between the multiplicative and additive models were small (Figure 6A; Table S3; $p = 0.30$ for all data together and $p > 0.05$ in all individual sessions; sign test). The affine model, on the other hand, performed significantly better than either the additive model (Figure 6B; Table S3; $p < 10^{-12}$ for all data together and $p < 0.008$ in all individual sessions; sign test) or the multiplicative model (Figure 6C; Table S3; $p < 10^{-16}$ for all data together and $p < 0.002$ in all individual sessions; sign test). Again, the extended multiplicative model did not fare as well as the affine model (Figures S2E–S2G; Table S4).

The affine model also outperformed the additive and multiplicative models in predicting noise correlations in single-unit data from quietly awake mice. As expected on the basis of lower firing rates (de la Rocha et al., 2007; Dorn and Ringach, 2003), measured correlations were considerably lower than in the multiunit data from anesthetized cats (noise-correlation means of 0.09 versus 0.38). Nonetheless, they could still be used to distinguish the performance of the models. As with the cat data, the affine model trumped both the additive model (Figures S5, $p < 10^{-4}$ for all data together, $p < 0.005$ in two individual sessions, and $p < 0.04$ in two others; sign test on the squared errors between measured and predicted noise correlations) and the multiplicative model (Figures S5, $p < 10^{-31}$ for all data together and $p < 0.001$ in all individual sessions; sign test).

Effect of Multiplicative Gain and Additive Offset on Population Coding

We have shown that cortical population variability can be well described by two sources of shared fluctuation: multiplicative and additive. How do these two sources of fluctuation impact information coding? To address this question, we used the responses of a neuronal population governed by the affine model to distinguish stimuli that differ subtly in orientation or contrast. To quantify this aspect of population decoding, we used a linear discriminability measure (Averbeck and Lee, 2006; Poor, 1994):

$$d^2 = (\bar{\boldsymbol{n}}_2 - \bar{\boldsymbol{n}}_1)^T \Sigma^{-1} (\bar{\boldsymbol{n}}_2 - \bar{\boldsymbol{n}}_1). \qquad \text{(Equation 3)}$$

Here $\bar{\boldsymbol{n}}_1$ and $\bar{\boldsymbol{n}}_2$ are the trial-averaged population response vectors to stimuli 1 and 2, and Σ is the population covariance matrix, derived from all presentations of the two stimuli. Since $\bar{\boldsymbol{n}}_1$, $\bar{\boldsymbol{n}}_2$, and Σ can be analytically calculated under the affine model (Supplemental Information: analytic calculation of pairwise correlations), we can estimate the dependence of d^2 on model parameters. For comparison, we also evaluated this measure for a population of independent, uncorrelated neurons having the equivalent amount of variability. To obtain a discriminability measure d^2_{shuffled} based on those uncorrelated responses, we set all off-diagonal values of the covariance matrix Σ (corresponding to correlations between neurons) to zero. Depending on the structure of the correlation, its value can be superior or inferior to d^2 (Abbott and Dayan, 1999; Averbeck et al., 2006; Moreno-Bote et al., 2014).

We first considered the effect of changing the mean of either the additive offset or the multiplicative gain. Increasing the mean of the multiplicative gain enhanced coding, whereas increasing the mean additive offset slightly degraded it (data not shown). This accords with classic studies of tuning

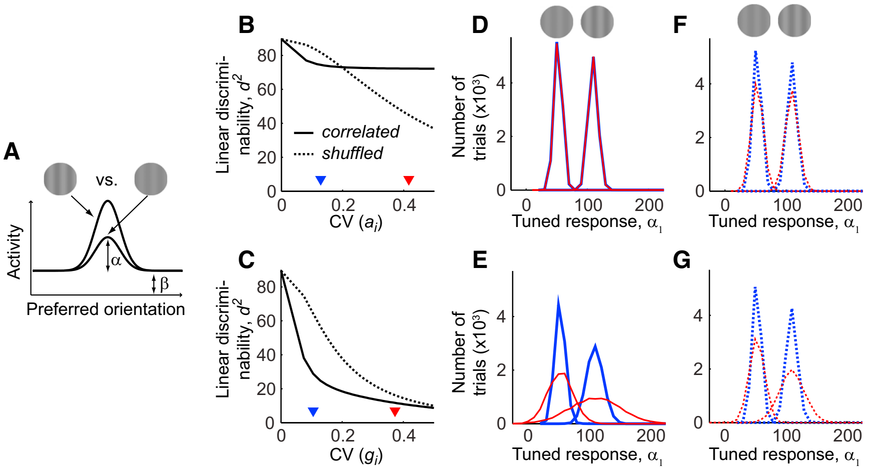

Figure 7. Effects of Fluctuations in Multiplicative Gain and Additive Offset on Contrast Coding

(A) Cartoon depicting the contrast discrimination task. The simulated activity of a homogenous neuronal population was used to discriminate two gratings of the same orientation at 6% and 12% contrasts; curves indicate population tuning curves in response to the two stimuli to be discerned. The population tuning curve on each trial is fitted as a linear combination of a unit Gaussian and a constant offset.

(B and C) Discriminability measure d^2 (solid) and the corresponding d^2_{shuffled} (dashed) between population responses in the discrimination task as a function of the coefficients of variation (CV) of the additive offset (B) and the multiplicative gain (C).

(D) Distributions of tuned-response amplitudes α from simulated population activity on 10^4 trials of a discrimination task for a low value (blue) and a high value (red) of CV(a_i). The two values used are marked by triangles of the same color in (B).

(E) As in (D), but for two different values of CV(g_i), the variability of shared multiplicative fluctuations. The two values are marked by triangles of the same color in (C).

(F and G) As in (D) and (E), but for their uncorrelated counterparts.

curves: the steeper the tuning curve and the lower the baseline, the larger the decoding accuracy (Dayan and Abbott, 2001).

We next considered the effect of trial-to-trial fluctuations in the additive and multiplicative terms. We simulated a contrast discrimination task (Figure 7A) and an orientation discrimination task (Figure 8A). The contrast discrimination task involved distinguishing two gratings of the same orientation, but different contrasts (6% and 12%); the orientation discrimination task involved distinguishing two 12%-contrast gratings whose orientations differed by either 6° or 90°.

In the contrast discrimination task, shared multiplicative fluctuations had a much larger effect on stimulus coding than additive ones. Increasing the variability of the additive offset hardly had any impact on discriminability, triggering a small decline that became noticeable only after trial-shuffling, when variability was uncorrelated across neurons (Figure 7B). On the other hand, increasing the variability of the multiplicative gain sharply decreased discriminability; e.g., discriminability dropped by a factor of 5 when the coefficient of variation of g_i increased from 0 to 0.5. This decline was reduced in d^2_{shuffled}, indicating that it is specific to multiplicative variability that is shared across neurons (Figure 7C). These trends also held for tasks involving different pairs of contrasts (Figures S8B–S8D).

To understand why contrast discrimination would particularly suffer from shared multiplicative fluctuations, we fit the simulated population response on a single trial as a linear combination of a unit circular Gaussian and a constant offset (Figure 7A). Population activity on each trial was thus summarized by two parameters: the tuned amplitude α and the baseline amplitude β. Discriminability could then be understood from the overlap of the distributions of the tuned responses α to the two stimuli: the more they overlap, the lower is discriminability. Increasing the variability of the additive offset hardly altered the shapes of these distributions, explaining the negligible effect of additive fluctuations on coding (Figure 7D). Rather, additive fluctuations affected the baseline amplitude β, which cannot be used to discriminate the two stimuli (data not shown). By contrast, increasing the variability of the multiplicative gain broadened the distributions of the tuned amplitudes α, making them much harder to discern (Figure 7E).

We obtained further insight into the effect of correlations induced by shared additive and multiplicative fluctuations by comparing them to the effect of equivalent uncorrelated variability using a shuffling analysis (Figures 7F and 7G). Comparing the α distributions calculated from the additive-correlated responses to stimuli 1 and 2 (Figure 7D) to their shuffled counterparts (Figure 7F) showed that additive-correlated variability was less detrimental than its shuffled equivalent (see also Figure S8M). By contrast, the α distributions obtained with shared multiplicative fluctuations (Figure 7E) had a greater overlap than the ones from the corresponding shuffled trials (Figure 7G). This indicates that in the contrast discrimination task, additive-correlated variability had a minor effect, but multiplicative-correlated variability worsened discriminability.

The effect of shared fluctuations on the orientation discrimination task depended on the type of fluctuations and the difficulty of the task (Figure 8). Shared additive fluctuations had no effect on discriminability, which deteriorated only after trial-shuffling, when variability was no longer shared among neurons (Figures 8B and 8C). Shared multiplicative fluctuations, on the other hand, had large consequences that depended on the difficulty of the task (Figures 8D and 8E). For most orientation differences, they had a strong detrimental effect, which was reduced by trial-shuffling (Figures 8D, S8G, S8H, S8K, and S8L). Yet, when the task involved discriminating stimuli of fine orientation difference, performance was actually worsened by trial-shuffling (Figures 8E, S8E, and S8I), indicating that shared multiplicative fluctuations allow better fine-orientation discrimination than the equivalent uncorrelated variability.

To understand these effects, we fit population responses as a sum of responses to the two gratings being discriminated (Figure 8A) and plotted ellipses illustrating the means and

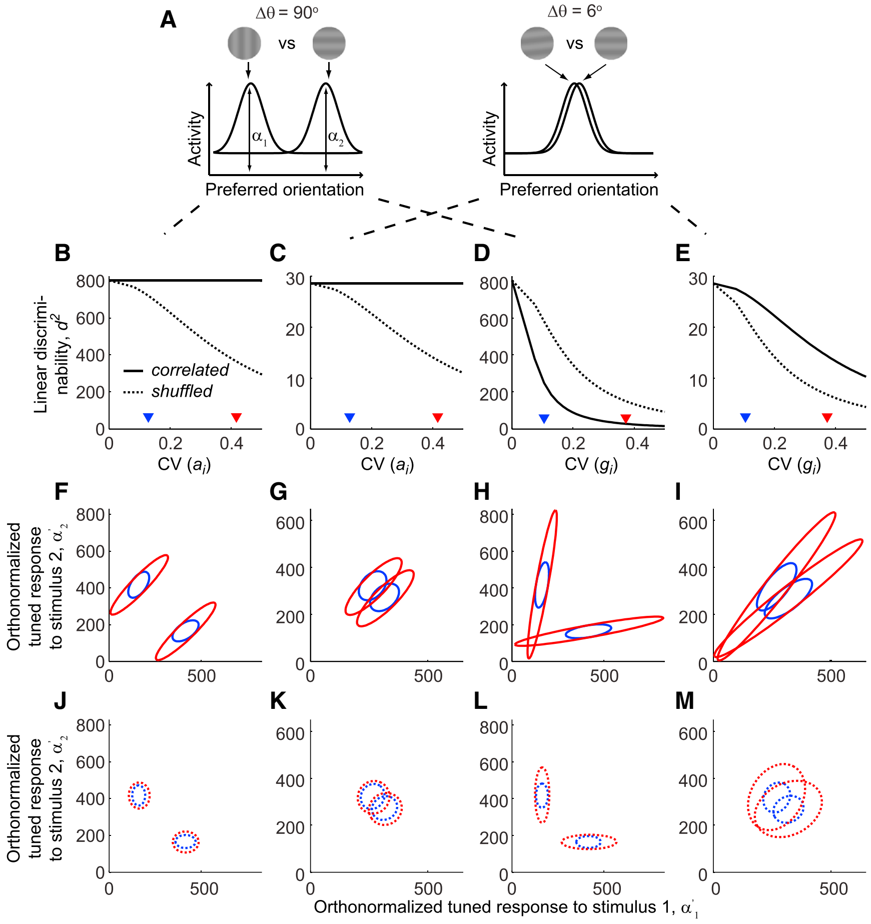

Figure 8. Effects of Fluctuations in Multiplicative Gain and Additive Offset on Orientation Coding

(A) Cartoon showing two orientation discrimination tasks. The activity of a homogenous neuronal population was used to distinguish two 12%-contrast gratings whose orientations differed by 90° and 6°. The population tuning curve on each trial was fitted as a linear combination of two unit Gaussians centered on the orientations of stimuli 1 and 2. α_1 and α_2 thus summarized the tuned amplitudes of the population activity to stimuli 1 and 2, respectively.

(B–E) Discriminability measure d^2 (solid line) and the corresponding $d^2_{shuffled}$ (dashed line) between population responses in the two tasks as a function of the coefficients of variation (CV) of the additive offset (B and C) and the multiplicative gain (D and E). The results from discriminating an orientation difference of 90° were plotted in (B) and (D); and results from fine discrimination (6°) were plotted in (C) and (E).

(F–I) Ellipses showing 3 SD contours of Gaussian fits to the distributions of population tuned responses to stimuli 1 and 2 on 10^4 trials of an orientation discrimination task at two different values of CV(a_i) (F and G) and CV(g_i) (H and I). For simple visualization, these plots show orthonormalized responses α'_1 and α'_2. The two values of CV(a_i) and CV(g_i) were marked by triangles in (B)–(E) with the same color coding.

(J–M) As in (F)–(I), but for trial-shuffled responses in which the equivalent variability occurs across uncorrelated neurons.

covariances of the population responses (Figures 8F–8M; see also Supplemental Information: decoding analysis with reduced dimensionality). For two gratings of very different orientations, the mean responses were far apart; shared multiplicative fluctuations elongated the two ellipses in such a way that they started to overlap, which reduced their discriminability (Figure 8H). Introducing the same amount of variability to uncorrelated neurons by trial-shuffling did not increase the overlap significantly (Figure 8L). Conversely, for fine-orientation discrimination, the mean responses to the two stimuli were very close (Figure 8I). Shared multiplicative fluctuations again elongated the two ellipses, but because the direction of this elongation was almost orthogonal to the separation between the centers, its effects on overlap were relatively minor. When the equivalent variability was applied independently to the neurons via trial-shuffling, the elongated ellipses were replaced by nearly circular shapes that showed greater overlap (Figure 8M). We observed similar trends in orientation discrimination tasks that involved gratings of a different contrast (Figures S8I–S8L).

In short, shared additive fluctuations are not detrimental to either contrast or orientation discrimination. Shared multiplicative fluctuations, on the other hand, are detrimental to the discrimination of contrast and of coarse orientation differences, but are less harmful to fine-orientation discrimination than an equivalent amount of uncorrelated variability.

DISCUSSION

We analyzed the activity of large neuronal populations in V1. We found that much of the trial-by-trial variability is shared across the population and can be summarized using only two global factors: additive and multiplicative. These results may reconcile the long-held view that variability is additive (Arieli et al., 1996; Schölvinck et al., 2015) with subsequent claims of it being predominantly multiplicative (Ecker et al., 2014; Goris et al., 2014). The former view hypothesizes that the large response variability on individual trials comes from adding ongoing cortical activity onto a deterministic sensory response. The latter postulates that much of the response variability arises from fluctuations in excitability. We argue that it is not one or the other, but a combination of the two.

Our data suggest that common multiplicative gain fluctuations play a dominant role in the structure of pairwise noise correlations. There are two long-standing hypotheses for the circuit mechanisms underlying the similarity of signal and noise correlations. The first is that correlations arise from synaptic connectivity patterns. In visual cortex, neurons with similar orientation preferences are preferentially connected, thus co-tuned neurons will also share a larger fraction of common input from cells of similar sensory preference (Ko et al., 2011), increasing their correlations. The second is that correlations reflect common

modulation by multiplicative gain fluctuations (Brody, 1999). Indeed, even a pair of neurons that are unconnected and share no inputs would exhibit noise correlations if they experienced common multiplicative modulation, and the strength of these correlations would be stronger for neurons with similar sensory tuning. Our data provide evidence in favor of both hypotheses, but ascribe a larger role to the mechanism of shared multiplicative fluctuations. Indeed, the affine model generated a good fit to the dependence of noise correlation on orientation tuning (Figures 4 and S6) and only a small residual was left to be explained. Intriguingly, this residual resembled the small, but significant, dependence of spontaneous correlations on tuning difference. The affine model could not possibly capture this dependence, because it has no way to preferentially assign shared factors as a function of tuning similarity. The similarity of spontaneous and signal correlations, matching the residual error in the affine model's prediction, thus suggests that specific synaptic connectivity does contribute to producing noise correlations, although this contribution is numerically smaller than the contribution of common gain fluctuations.

Our results lend weight to the multiplicative model, but also reveal its possible limitations. For example, shared multiplicative fluctuations alone were insufficient to predict the complex relationship between correlations, the sensory tuning of both units, and the stimulus (Figure 5). The affine model—by adding an additional common additive offset on each trial—resolved this limitation.

Nevertheless, there are certain features that may further improve the affine model. For instance, Okun et al. (2015) show that different neurons are coupled to global fluctuations differently. In the current affine model, only the additive component has a cell-coupling term that allows each neuron to be coupled to population activity to different degrees. We saw that it is possible to extend the multiplicative model in such a way; this extension improved the fit of the multiplicative model, but not to the extent that it matched the affine model (Figure S2). Future work could explore the possibility of combining the extended multiplicative model and the additive model; however, this would require substantially greater amounts of single-unit data than analyzed here.

What candidate circuit mechanisms might underlie the multiplicative and additive fluctuations described here? A possibility lies in different interneuron classes: activation and inactivation of parvalbumin- or somatostatin-positive interneurons have been variously suggested to have multiplicative, additive, and combined (affine) effects on the firing rates of pyramidal cells (Atallah et al., 2012; Lee et al., 2012; Wilson et al., 2012). Another possibility lies in top-down connections from higher order cortices and thalamus. These inputs, which target distal apical dendrites in layer 1, can rarely elicit spikes in pyramidal cells, but may boost the gain of pyramidal cells' responses to more proximal sensory inputs (Larkum, 2013; Larkum et al., 1999, 2004). Such top-down connections are believed to play a role in attentional modulation of V4 firing (Armstrong and Moore, 2007; Moore and Armstrong, 2003), which has been reported to have both a multiplicative and an additive effect on visual responses (Boynton, 2009; Buracas and Boynton, 2007; Murray, 2008; Reynolds and Heeger, 2009; Thiele et al., 2009; Williford and Maunsell, 2006). In addition, multiplicative effects have been observed in other contexts such as normalization (Busse et al., 2009; Carandini and Heeger, 2012). The multiplicative and additive fluctuations might also share circuits with those that modulate responses based on locomotion, which has both an additive and a divisive effect (Ayaz et al., 2013).

Additive and multiplicative fluctuations have very different consequences for the cortical coding of sensory information. An analytically tractable model based on our experimental results revealed that for both contrast and orientation discriminations, additive fluctuations had negligible effect on the discriminability of sensory stimuli, whereas multiplicative fluctuations had a much larger effect. These results have a simple intuitive explanation. Variability in population activity reduces the ability to discriminate stimuli, when it means that a single population-firing pattern can be induced by two different sensory stimuli. A change in multiplicative gain can affect population activity in a very similar way to a change in stimulus contrast; it is thus not surprising that contrast discrimination is impaired strongly by multiplicative fluctuations. Likewise, the small effect of additive fluctuations may be understood from the fact that the resulting changes in baseline could not have been generated by any of the stimuli presented.

While correlated fluctuations were originally believed to only worsen stimulus discriminability (Zohary et al., 1994), responses with correlated variability can often outperform responses with the same amount of uncorrelated variability (Abbott and Dayan, 1999; Averbeck and Lee, 2006; Moreno-Bote et al., 2014). This does not mean that correlated variability helps discrimination (compared to zero variability); it means that its correlation structure interferes less with stimulus coding than might otherwise be expected. Indeed, we found that correlations frequently help discriminability. Shared additive fluctuations, for example, had a less detrimental effect on discriminability than their uncorrelated counterparts. This may be because the effect of shared additive fluctuations lies along a different direction in population vector space than differences between stimuli; shuffling thus adds variance to a dimension that can interfere with stimulus coding (Figure S8M). For shared multiplicative fluctuations, the effect of correlations on orientation discrimination depended on the difficulty of the discrimination task. For easy tasks (large orientation differences), correlations hurt. In this case, the pools of neurons responding to the two very different stimuli are largely distinct; the arguments of Zohary et al. (1994) therefore apply, and correlations cause averages over populations to be taken less accurately. For fine discrimination, however, correlations improved performance, possibly because the multiplicative fluctuations move population responses in a different direction to differences in stimulus orientation (akin to differences in contrast). Our decoding results therefore suggest that the nature of shared variability in visual cortical populations is well-suited for stimulus discrimination: additive variability has little consequence, while multiplicative-derived correlations benefit fine-orientation discrimination over uncorrelated variability.

But why should shared multiplicative and additive variability occur at all? Why not simply have private variability or no variability? Multiplicative variability might reflect the same circuit mechanisms that are responsible for top-down processes,

such as attention. It has been suggested that visual attention modulates visual cortical responses in a similar manner to an increase in stimulus contrast, which could explain its primarily multiplicative effect similar to a contrast change (Reynolds and Heeger, 2009). While fluctuations in this process would clearly impair fine-contrast discrimination, contrast discrimination may be a task only rarely required. Indeed, much of the visual system appears to be geared toward making contrast-invariant judgments (Finn et al., 2007). Because our results show that multiplicative fluctuations have little effect on fine-orientation discrimination, we conclude that multiplicative fluctuations might allow the visual system to modulate the salience of visual stimuli, while having relatively little impact on stimulus coding. Additive fluctuations may allow the population to include an additional dimension of salience or other non-sensory factors, with only minor impact on representation of sensory information.

EXPERIMENTAL PROCEDURES

Anesthetized cat recordings were approved by the Animal Care and Use Committee of the Smith-Kettlewell Eye Research Institute. Experimental methods have been previously described by Busse et al. (2009). Briefly, responses, primarily from layers 2/3, were recorded with a 10-by-10 electrode array. All threshold crossings on each channel were pooled and only orientation-tuned sites were considered in subsequent analyses. Sequences of 2 s contrast-reversing oriented gratings and plaids, interspersed with 2 s blanks, were shown in random order in blocks (see Supplemental Information: anesthetized cat recordings).

Awake mouse recordings were conducted under personal and project licenses issued by the Home Office, in accordance with the UK Animals (Scientific Procedures) Act 1986. A head plate with a recording chamber was affixed to the skull. After 3 days of recovery and 3 head-restraint acclimatization sessions, a craniectomy (and durotomy, if necessary) was made over the left V1. The animal was allowed to recover for at least 1.5 hr before the recording. Multisite silicon probes were inserted to a depth of 500–800 μm (median 615 μm). Animals were judged to be quietly awake by video monitoring. Spikes were detected using NDManager (Hazan et al., 2006) and clustered using KlustaKwik (Harris et al., 2000; Kadir et al., 2014), followed by manual adjustment using KlustaViewa (Rossant and Harris, 2013). Detailed analysis was carried out only on well-isolated units that showed consistent firing throughout a recording session. Sequences of 1 s oriented drifting gratings, interspersed with either 1 or 6 s blanks were shown on three liquid-crystal display monitors, covering a field of view of ~120° × 60° that extended in front and to the right of the animal (see Supplemental Information: awake mouse recordings).

Population tuning curves $\boldsymbol{R}_i$ were fit to population responses to plaid stimuli as a linear combination of prototypical response to the component gratings and a constant baseline shift. The percentage of variance of the population activity that could be explained by the population tuning curve analysis on each trial was estimated as $1-(\sum_c (r_{c,i}-R_{c,i})^2)/(\sum_c (r_{c,i}-\bar{r}_i)^2)$, where $r_{c,i}$ is the normalized firing rate for each site c and trial i, and $\bar{r}_i$ is the measured site-averaged response on trial i (see Supplemental Information: population tuning curve analysis).

Full details of the models considered in this study can be found in Supplemental Information: the models. Briefly, we denote the experimentally measured spike counts of unit c (single neuron or multiunit) on trial i as $N_{c,i}$ and the stimulus presented on trial i as $s(i)$. Each model predicts an expected spike count $f_{c,i}$ of unit c on trial i that approximates $N_{c,i}$.

In the independent model, the expected spike count on each trial is a deterministic quantity:

$$f_{c,i} = d_{c,s(i)}, \qquad \text{(Equation 4)}$$

where the matrix $d_{c,s(i)}$ is estimated as the trial-averaged spike count of unit c to stimulus $s(i)$.

In the additive model, the expected spike count is

$$f_{c,i} = d_{c,s(i)} + a_i h_c, \qquad \text{(Equation 5)}$$

where $d_{c,s}$ represents the sensory drive of unit c from stimulus s, a_i the common additive offset on each trial i, and h_c the degree to which each unit c is susceptible to this offset. The total number of parameters in this model is $M_{\text{units}}M_{\text{stimuli}} + M_{\text{trials}} + M_{\text{units}}$. $d_{c,s}$ is estimated as in the independent model, and the parameters a_i and h_c were fit by least-squares.

In the multiplicative model, the expected spike count is given by

$$f_{c,i} = g_i d_{c,s(i)}. \qquad \text{(Equation 6)}$$

g_i is the common multiplicative gain on trial i. Note that unlike the model of Goris et al. (2014), where on each trial each cell has its own (private) gain (i.e., $f_{c,i} = g_{c,i} d_{c,s(i)}$), we propose a common multiplicative gain that is shared across the population. This model contains $M_{\text{units}}M_{\text{stimuli}} + M_{\text{trials}}$ parameters, which were fit by least-squares.

The affine model incorporates both the additive and multiplicative components:

$$f_{c,i} = g_i d_{c,s(i)} + a_i h_c. \qquad \text{(Equation 7)}$$

The total number of parameters in this model is $M_{\text{units}}M_{\text{stimuli}} + 2M_{\text{trials}} + M_{\text{units}}$. We fit the affine model by an alternation method that was repeated until the convergence criterion was met, specifically that the difference in squared error per unit per trial $\sum_{c,i}(f_{c,i}-N_{c,i})^2/M_{\text{units}}M_{\text{trials}}$ between two iterations was lower than 10^{-10}. Typically this took several hundred iterations.

The predictions of these models represent expected spike counts rather than actual integer observations. To generate spike counts $n_{c,i}$ from each model, we used a negative-binomial spike generator with mean $f_{c,i}$ and a Fano factor parameter $F_{c,s(i)}$ that was estimated for each unit c and stimulus s by maximum likelihood (see Supplemental Information: spike count generator). Spike counts generated by this method were used for the analyses in Figures 3, 4, and 5.

We assessed the models' goodness of fit by cross-validating the simulated spike count $n_{c,i}$ (see Supplemental Information: cross-validation; Figure S1). Model performance was assessed for each unit c by quality index $q_c = 1-(\sum_i e_{c,i}^2/\sum_i e_{c,i}'^2)$, where $e_{c,i}'^2$ was the squared error of the independent model. This cross-validation method was used to generate the plots in Figures 2E–2G, 6, and S2B–S2G; only units that showed shared variability ($q_c > 0.1$ for at least one of the models) were included in statistical analysis.

To estimate how the linear discriminability measure d'^2 depends on the fluctuations of additive offset and multiplicative gain, we constructed a homogeneous neural population with translation-invariant orientation tuning curves (see Supplemental Information: decoding). To visualize the population patterns produced by this population, we simulated population responses on 10^4 trials of the contrast and orientation discrimination tasks, assuming Gaussian distributions for both multiplicative gain and additive offset. The corresponding uncorrelated responses were obtained by trial-shuffling. These population responses were then projected onto a low-dimensional space for visualization (see Supplemental Information: decoding analysis with reduced dimensionality).

SUPPLEMENTAL INFORMATION

Supplemental Information includes Supplemental Experimental Procedures, eight figures, and four tables and can be found with this article online at http://dx.doi.org/10.1016/j.neuron.2015.06.035.

AUTHOR CONTRIBUTIONS

Conceptualization: I.-C.L., M.O., M.C., and K.D.H.; Methodology: I.-C.L., M.O., M.C., and K.D.H.; Software: I.-C.L.; Formal Analysis: I.-C.L., M.O., M.C., and K.D.H.; Investigation: I.-C.L. and M.O.; Resources: M.C.; Writing – Original Draft: I.-C.L., M.O., M.C., and K.D.H.; Writing – Reviewing & Editing: I.-C.L., M.O., M.C., and K.D.H.; Visualization: I.-C.L.; Supervision: M.C. and K.D.H.; Project Administration: M.C. and K.D.H.; Funding Acquisition: M.C. and K.D.H.

ACKNOWLEDGMENTS

We thank L. Busse for providing the cat data acquired for her previously published study, and members of our laboratory for useful suggestions. K.D.H. and M.C. are jointly funded by the Wellcome Trust (grants 095668 and 095669) and by the Simons Collaboration on the Global Brain (grant 325512). M.C. holds the GlaxoSmithKline/Fight for Sight Chair in Visual Neuroscience.

Received: October 1, 2014
Revised: March 4, 2015
Accepted: June 24, 2015
Published: July 23, 2015

REFERENCES

Abbott, L.F., and Dayan, P. (1999). The effect of correlated variability on the accuracy of a population code. Neural Comput. *11*, 91–101.

Arieli, A., Sterkin, A., Grinvald, A., and Aertsen, A. (1996). Dynamics of ongoing activity: explanation of the large variability in evoked cortical responses. Science *273*, 1868–1871.

Armstrong, K.M., and Moore, T. (2007). Rapid enhancement of visual cortical response discriminability by microstimulation of the frontal eye field. Proc. Natl. Acad. Sci. USA *104*, 9499–9504.

Atallah, B.V., Bruns, W., Carandini, M., and Scanziani, M. (2012). Parvalbumin-expressing interneurons linearly transform cortical responses to visual stimuli. Neuron *73*, 159–170.

Averbeck, B.B., and Lee, D. (2006). Effects of noise correlations on information encoding and decoding. J. Neurophysiol. *95*, 3633–3644.

Averbeck, B.B., Latham, P.E., and Pouget, A. (2006). Neural correlations, population coding and computation. Nat. Rev. Neurosci. *7*, 358–366.

Ayaz, A., Saleem, A.B., Schölvinck, M.L., and Carandini, M. (2013). Locomotion controls spatial integration in mouse visual cortex. Curr. Biol. *23*, 890–894.

Boynton, G.M. (2009). A framework for describing the effects of attention on visual responses. Vision Res. *49*, 1129–1143.

Brody, C.D. (1999). Correlations without synchrony. Neural Comput. *11*, 1537–1551.

Buracas, G.T., and Boynton, G.M. (2007). The effect of spatial attention on contrast response functions in human visual cortex. J. Neurosci. *27*, 93–97.

Busse, L., Wade, A.R., and Carandini, M. (2009). Representation of concurrent stimuli by population activity in visual cortex. Neuron *64*, 931–942.

Carandini, M. (2004). Amplification of trial-to-trial response variability by neurons in visual cortex. PLoS Biol. *2*, E264.

Carandini, M., and Heeger, D.J. (2012). Normalization as a canonical neural computation. Nat. Rev. Neurosci. *13*, 51–62.

Cohen, M.R., and Kohn, A. (2011). Measuring and interpreting neuronal correlations. Nat. Neurosci. *14*, 811–819.

Cotton, R.J., Froudarakis, E., Storer, P., Saggau, P., and Tolias, A.S. (2013). Three-dimensional mapping of microcircuit correlation structure. Front. Neural Circuits *7*, 151.

Dayan, P., and Abbott, L.F. (2001). Theoretical neuroscience: computational and mathematical modeling of neural systems (Cambridge, Mass.: Massachusetts Institute of Technology Press).

de la Rocha, J., Doiron, B., Shea-Brown, E., Josić, K., and Reyes, A. (2007). Correlation between neural spike trains increases with firing rate. Nature *448*, 802–806.

Deweese, M.R., and Zador, A.M. (2004). Shared and private variability in the auditory cortex. J. Neurophysiol. *92*, 1840–1855.

Dinstein, I., Heeger, D.J., and Behrmann, M. (2015). Neural variability: friend or foe? Trends Cogn. Sci. *19*, 322–328.

Dorn, J.D., and Ringach, D.L. (2003). Estimating membrane voltage correlations from extracellular spike trains. J. Neurophysiol. *89*, 2271–2278.

Ecker, A.S., Berens, P., Cotton, R.J., Subramaniyan, M., Denfield, G.H., Cadwell, C.R., Smirnakis, S.M., Bethge, M., and Tolias, A.S. (2014). State dependence of noise correlations in macaque primary visual cortex. Neuron *82*, 235–248.

Finn, I.M., Priebe, N.J., and Ferster, D. (2007). The emergence of contrast-invariant orientation tuning in simple cells of cat visual cortex. Neuron *54*, 137–152.

Goris, R.L., Movshon, J.A., and Simoncelli, E.P. (2014). Partitioning neuronal variability. Nat. Neurosci. *17*, 858–865.

Harris, K.D., Henze, D.A., Csicsvari, J., Hirase, H., and Buzsáki, G. (2000). Accuracy of tetrode spike separation as determined by simultaneous intracellular and extracellular measurements. J. Neurophysiol. *84*, 401–414.

Hazan, L., Zugaro, M., and Buzsáki, G. (2006). Klusters, NeuroScope, NDManager: a free software suite for neurophysiological data processing and visualization. J. Neurosci. Methods *155*, 207–216.

Heggelund, P., and Albus, K. (1978). Response variability and orientation discrimination of single cells in striate cortex of cat. Exp. Brain Res. *32*, 197–211.

Jermakowicz, W.J., Chen, X., Khaytin, I., Bonds, A.B., and Casagrande, V.A. (2009). Relationship between spontaneous and evoked spike-time correlations in primate visual cortex. J. Neurophysiol. *101*, 2279–2289.

Kadir, S.N., Goodman, D.F., and Harris, K.D. (2014). High-dimensional cluster analysis with the masked EM algorithm. Neural Comput. *26*, 2379–2394.

Katzner, S., Nauhaus, I., Benucci, A., Bonin, V., Ringach, D.L., and Carandini, M. (2009). Local origin of field potentials in visual cortex. Neuron *61*, 35–41.

Kenet, T., Bibitchkov, D., Tsodyks, M., Grinvald, A., and Arieli, A. (2003). Spontaneously emerging cortical representations of visual attributes. Nature *425*, 954–956.

Ko, H., Hofer, S.B., Pichler, B., Buchanan, K.A., Sjöström, P.J., and Mrsic-Flogel, T.D. (2011). Functional specificity of local synaptic connections in neocortical networks. Nature *473*, 87–91.

Kohn, A., and Smith, M.A. (2005). Stimulus dependence of neuronal correlation in primary visual cortex of the macaque. J. Neurosci. *25*, 3661–3673.

Larkum, M. (2013). A cellular mechanism for cortical associations: an organizing principle for the cerebral cortex. Trends Neurosci. *36*, 141–151.

Larkum, M.E., Zhu, J.J., and Sakmann, B. (1999). A new cellular mechanism for coupling inputs arriving at different cortical layers. Nature *398*, 338–341.

Larkum, M.E., Senn, W., and Lüscher, H.R. (2004). Top-down dendritic input increases the gain of layer 5 pyramidal neurons. Cereb. Cortex *14*, 1059–1070.

Lee, S.H., Kwan, A.C., Zhang, S., Phoumthipphavong, V., Flannery, J.G., Masmanidis, S.C., Taniguchi, H., Huang, Z.J., Zhang, F., Boyden, E.S., et al. (2012). Activation of specific interneurons improves V1 feature selectivity and visual perception. Nature *488*, 379–383.

Luczak, A., Barthó, P., and Harris, K.D. (2009). Spontaneous events outline the realm of possible sensory responses in neocortical populations. Neuron *62*, 413–425.

Luczak, A., Bartho, P., and Harris, K.D. (2013). Gating of sensory input by spontaneous cortical activity. J. Neurosci. *33*, 1684–1695.

Mainen, Z.F., and Sejnowski, T.J. (1995). Reliability of spike timing in neocortical neurons. Science *268*, 1503–1506.

Moore, T., and Armstrong, K.M. (2003). Selective gating of visual signals by microstimulation of frontal cortex. Nature *421*, 370–373.

Moreno-Bote, R., Beck, J., Kanitscheider, I., Pitkow, X., Latham, P., and Pouget, A. (2014). Information-limiting correlations. Nat. Neurosci. *17*, 1410–1417.

Murray, S.O. (2008). The effects of spatial attention in early human visual cortex are stimulus independent. J. Vis. *8*, 1–11.

Niell, C.M., and Stryker, M.P. (2008). Highly selective receptive fields in mouse visual cortex. J. Neurosci. *28*, 7520–7536.

CellPress

Okun, M., Yger, P., Marguet, S.L., Gerard-Mercier, F., Benucci, A., Katzner, S., Busse, L., Carandini, M., and Harris, K.D. (2012). Population rate dynamics and multineuron firing patterns in sensory cortex. J. Neurosci. *32*, 17108–17119.

Okun, M., Steinmetz, N.A., Cossell, L., Iacaruso, M.F., Ko, H., Barthó, P., Moore, T., Hofer, S.B., Mrsic-Flogel, T.D., Carandini, M., and Harris, K.D. (2015). Diverse coupling of neurons to populations in sensory cortex. Nature *521*, 511–515.

Ponce-Alvarez, A., Thiele, A., Albright, T.D., Stoner, G.R., and Deco, G. (2013). Stimulus-dependent variability and noise correlations in cortical MT neurons. Proc. Natl. Acad. Sci. USA *110*, 13162–13167.

Poor, H.V. (1994). An Introduction to Signal Detection and Estimation (Springer New York).

Reynolds, J.H., and Heeger, D.J. (2009). The normalization model of attention. Neuron *61*, 168–185.

Ringach, D.L. (2009). Spontaneous and driven cortical activity: implications for computation. Curr. Opin. Neurobiol. *19*, 439–444.

Rossant, C., and Harris, K.D. (2013). Hardware-accelerated interactive data visualization for neuroscience in Python. Front. Neuroinform. *7*, 36.

Schölvinck, M.L., Saleem, A.B., Benucci, A., Harris, K.D., and Carandini, M. (2015). Cortical state determines global variability and correlations in visual cortex. J. Neurosci. *35*, 170–178.

Shadlen, M.N., and Newsome, W.T. (1998). The variable discharge of cortical neurons: implications for connectivity, computation, and information coding. J. Neurosci. *18*, 3870–3896.

Thiele, A., Pooresmaeili, A., Delicato, L.S., Herrero, J.L., and Roelfsema, P.R. (2009). Additive effects of attention and stimulus contrast in primary visual cortex. Cereb. Cortex *19*, 2970–2981.

Tolhurst, D.J., Movshon, J.A., and Dean, A.F. (1983). The statistical reliability of signals in single neurons in cat and monkey visual cortex. Vision Res. *23*, 775–785.

Tsodyks, M., Kenet, T., Grinvald, A., and Arieli, A. (1999). Linking spontaneous activity of single cortical neurons and the underlying functional architecture. Science *286*, 1943–1946.

Vogels, R., Spileers, W., and Orban, G.A. (1989). The response variability of striate cortical neurons in the behaving monkey. Exp. Brain Res. *77*, 432–436.

Williford, T., and Maunsell, J.H. (2006). Effects of spatial attention on contrast response functions in macaque area V4. J. Neurophysiol. *96*, 40–54.

Wilson, N.R., Runyan, C.A., Wang, F.L., and Sur, M. (2012). Division and subtraction by distinct cortical inhibitory networks in vivo. Nature *488*, 343–348.

Zohary, E., Shadlen, M.N., and Newsome, W.T. (1994). Correlated neuronal discharge rate and its implications for psychophysical performance. Nature *370*, 140–143.

Neuron

Supplemental Information

The Nature of Shared Cortical Variability

I-Chun Lin, Michael Okun, Matteo Carandini, and Kenneth D. Harris

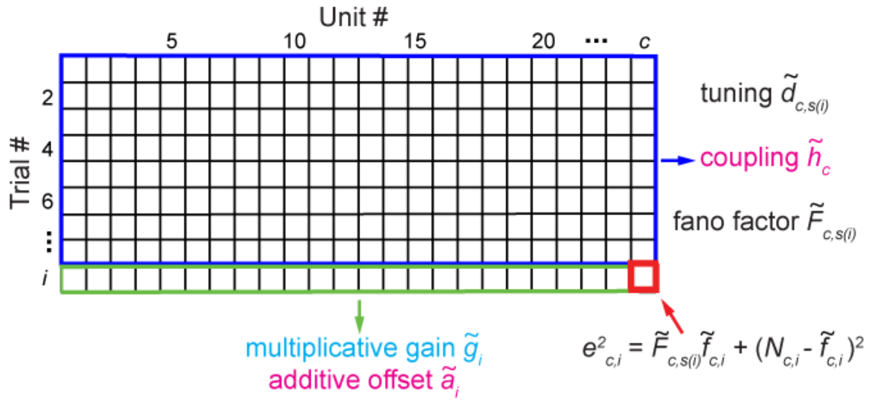

Figure S1 (related to Figures 2 and 6). Schematic of the cross-validation method used to evaluate the performance of the models for shared variability. We iterated over all elements of the matrix of units and trials, predicting the response of each element in the matrix from all other elements of the matrix. The predicted unit c is referred to as the 'test unit', and the predicted trial i is referred to as the 'test trial'. The remaining units were referred to as 'training units' and the remaining trials as 'training trials'. The data from all training trials (*blue block*) were used to estimate the stimulus-drive term $\tilde{d}_{c,s(i)}$, the fluctuation-coupling parameter $\tilde{h}_c$, and the Fano factor of the spike count generator $\tilde{F}_{c,s(i)}$. Then, the multiplicative gain $\tilde{g}_i$ and additive offset $\tilde{a}_i$ on the test trial were estimated from the activity of all training units on this trial (*green block*). Finally, a prediction $\tilde{f}_{c,i} = \tilde{g}_i\tilde{d}_{c,s(i)} + \tilde{a}_i\tilde{h}_c$ was obtained for the expected spike count of the test cell on the test trial. The squared error with respect to the test unit c on the test trial i (*red square*) was estimated as $e^2_{c,i} = \tilde{F}_{c,s(i)}\tilde{f}_{c,i} + \left(N_{c,i} - \tilde{f}_{c,i}\right)^2$ (see Supplemental Experimental Procedures: cross-validation). Cross-validated performance of each model was then assessed by quality index $q_c = 1 - \frac{\sum_i e^2_{c,i}}{\sum_i e'^2_{c,i}}$, where $e'^2_{c,i}$ is the squared error associated with the independent model.

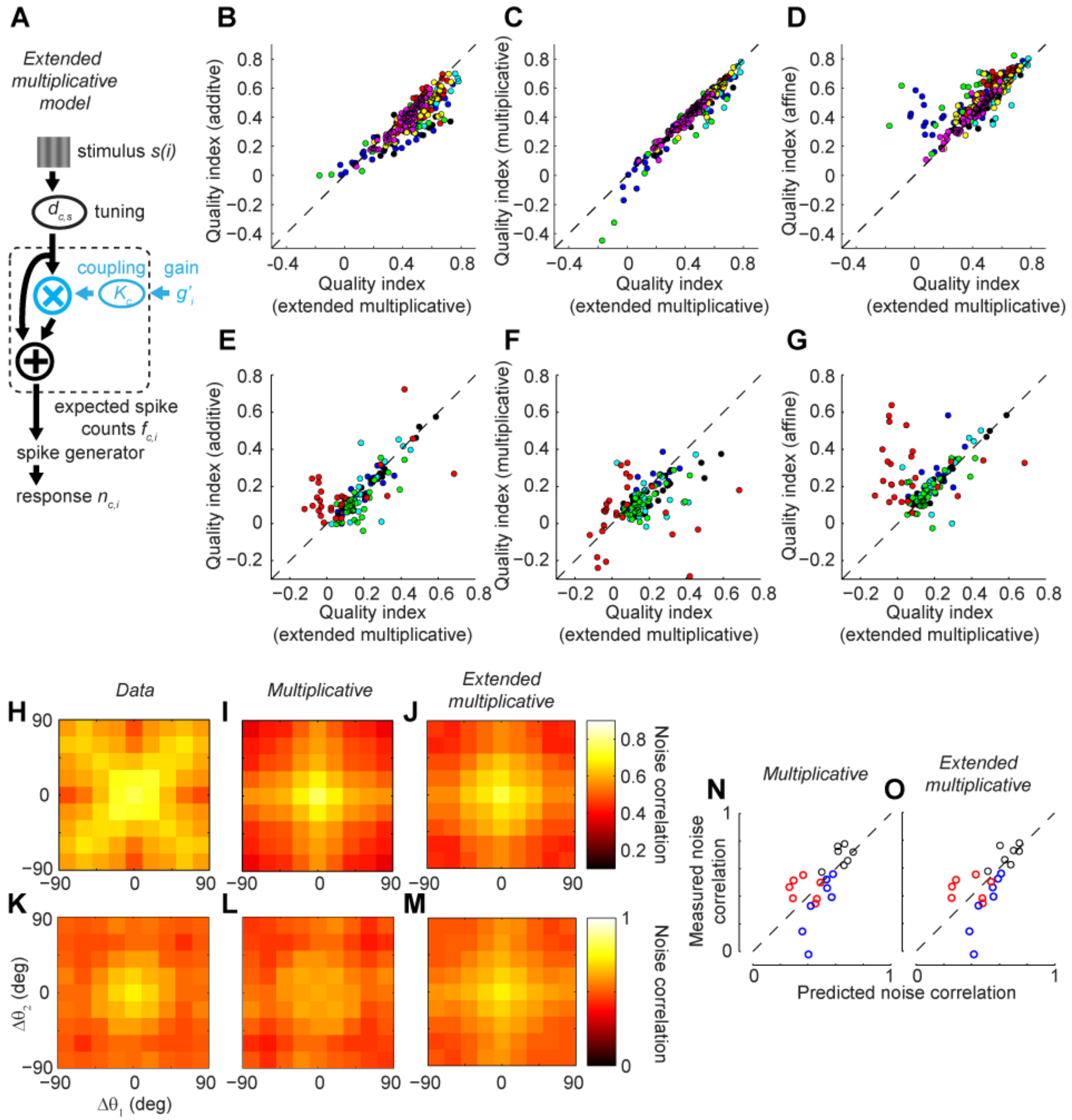

Figure S2 (related to Figures 2, 5, and 6). The extended multiplicative model. **(A)** In the extended multiplicative model, the expected spike count $f_{c,i}$ is given by $f_{c,i} = d_{c,s(i)}(1 + K_c g'_i)$. K_c determines how much a cell is coupled to the multiplicative gain g_i; K_c=0 reduces the model to the independent model, and K_c=1 to the multiplicative model with $g'_i = g_i - 1$. As in all other models, $f_{c,i}$ is passed into a stochastic spike count generator, which yields an integer spike count $n_{c,i}$ from a negative binomial distribution. (**B**-**D**) Cross-validated performance of the response $n_{c,i}$ generated by the extended multiplicative model versus the additive (B), multiplicative (C), and affine (D) models across 7 recording sessions in 3 anesthetized cats. Performance was measured by the quality index, which is zero or negative if the prediction is no better than the independent model, and equals 1 for a perfect prediction. Each *circle* represents the performance on one site across all trials in a session; sites from the same session share the

same color. Only sites that had quality index > 0.1 for at least one of the models were shown. (**E**-**G**) As in B-D, but for performance on single-unit data from quietly awake mouse recording (5 sessions in 4 mice). (**H**-**O**) The extended multiplicative model cannot capture the dependence of correlations on cell tuning and stimulus orientation in anesthetized cat data (cf. Figure 5). Pseudocolor representation of median noise correlation for all pairs of orientation-tuned sites as a function of $\Delta\theta_1$ and $\Delta\theta_2$ (H). Data were pooled across all contrasts and orientations. Session 83-7-5, 45 orientation-tuned sites. Predictions of the multiplicative and extended multiplicative models are plotted in I and J, respectively. Measured and predicted correlation matrices for session 83-10-15 (42 orientation-tuned sites) are shown in K-M; note the worse fit of the extended multiplicative than the affine models (see Figure 5). Scatter plots comparing the measured and predicted correlations for the multiplicative (N) and the extended multiplicative (O) models across 7 sessions in 3 cats; note again the poorer performance of the extended multiplicative than the affine models (Figure 5M). Each *circle* shows the measured and predicted noise correlations for one bin and one session; the median noise correlations in the center, corner, and edge bins are marked by *black*, *red*, and *blue circles*.

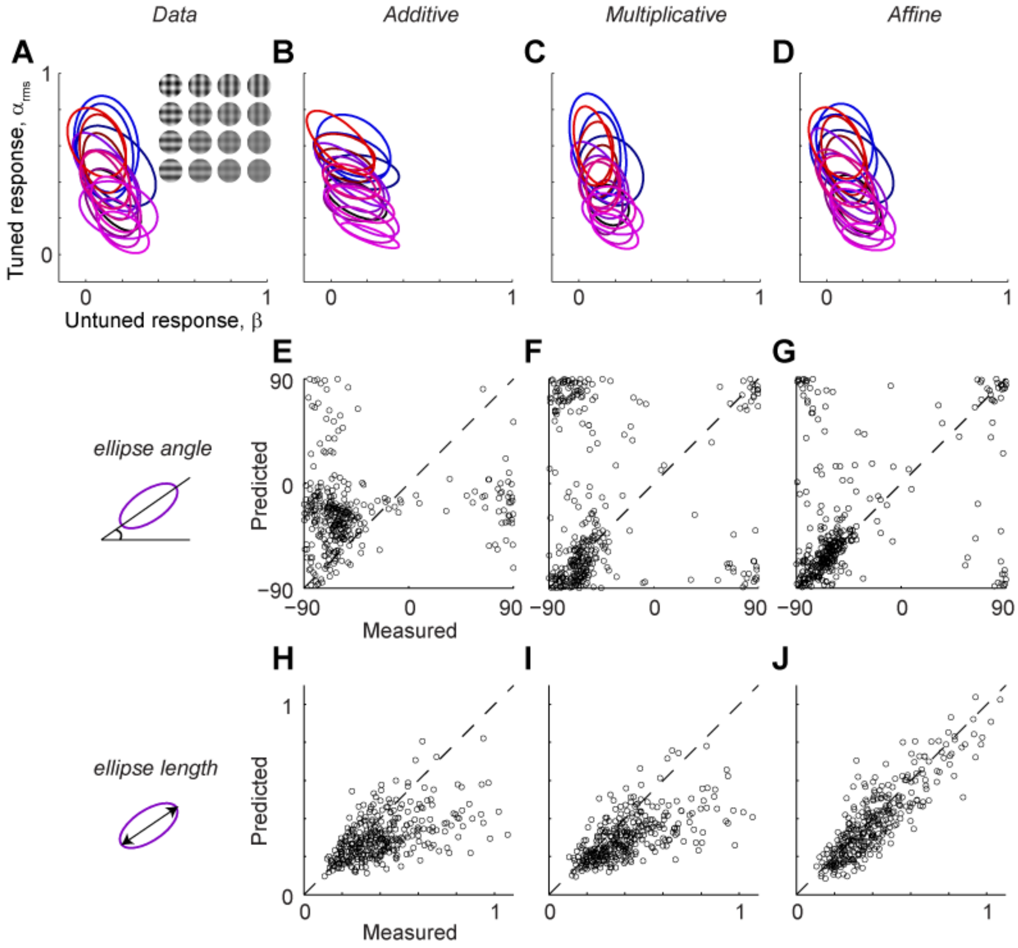

Figure S3 (related to Figure 3). Variability of population response to plaid stimuli. (**A**) Ellipses showing 1 SD contours of Gaussian fits to the distributions of the untuned response β and the average tuned response, defined as the root-mean square of the two tuned responses, $\alpha_{\rm rms} = \sqrt{\alpha_1^2 + \alpha_2^2}$, for an example recording session (session 83-7-5, 45 orientation-tuned sites, plaid angle = 90°). The 16 ellipses correspond to 16 plaid stimuli in which the contrasts of the two component gratings are varied independently. *Colors* indicate the component contrasts (RGB color code, *red* for contrast in grating 1, *blue* for grating 2). For each component-contrast combination, data were pooled across different component orientations. (**B**-**D**) Ellipses fitted to the predictions of the additive (B), multiplicative (C), and affine (D) models. (**E**-**G**) Comparison of the ellipse angles predicted by the three models versus experimental data across 7 sessions in 3 cats. Each plaid stimulus in each session contributes a *dot* for the ellipse fitted to the distribution of β versus $\alpha_{\rm rms}$. (**H**-**J**) Same as E-G, but for the comparison of the major-axis lengths of ellipses.

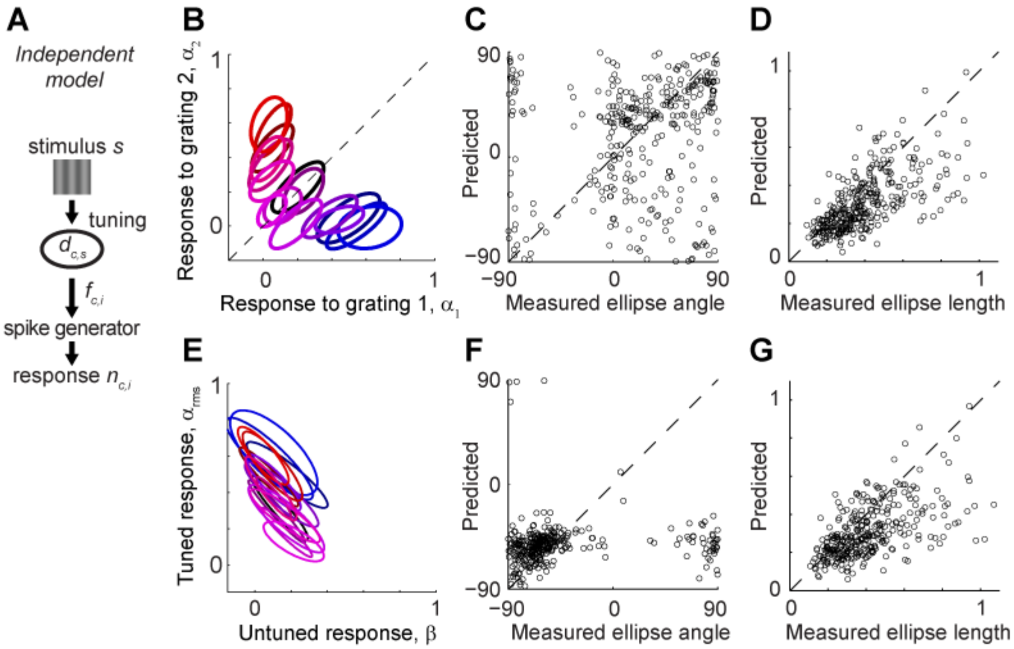

Figure S4 (related to Figure 3). The independent model. (**A**) In the independent model, the expected spike count $f_{c,i}$ is simply the trial-averaged spike count; as in other models, $f_{c,i}$ is passed into a stochastic spike count generator, yielding an integer spike count $n_{c,i}$ from a negative binomial distribution. (**B**) Ellipses showing 1 SD contours of Gaussian fits to the distributions of simulated population responses to gratings 1 and 2 (α_1 and α_2) for session 83-7-5 (45 orientation-tuned sites, plaid angle = 90°). The 16 ellipses correspond to 16 plaid stimuli in which the contrasts of the two component gratings are varied independently. *Colors* indicate the component contrasts (RGB color code with *red* encoding the contrast of grating 1 and *blue* encoding the contrast of grating 2). For each component-contrast combination, data were pooled across different component orientations. Note that a small upward tilt to the ellipses is seen; this does not result from correlated neuronal firing but rather from non-orthogonality of the basis vectors $\boldsymbol{G}(\theta_1)$ and $\boldsymbol{G}(\theta_2)$. (**C**) Comparison of the ellipse angles predicted by the independent model versus data from all 7 sessions in 3 cats. Each plaid stimulus in each session contributes a *dot* for the ellipse fitted to the distribution of α_1 vs α_2. (**D**) Same as C, but for the comparison of the major-axis lengths of ellipses. (**E**-**G**) As in B-D, but for the distributions of the untuned response β and the average tuned response, defined as the root-mean square of the two tuned responses, $\alpha_{\mathrm{rms}} = \sqrt{\alpha_1^2 + \alpha_2^2}$.

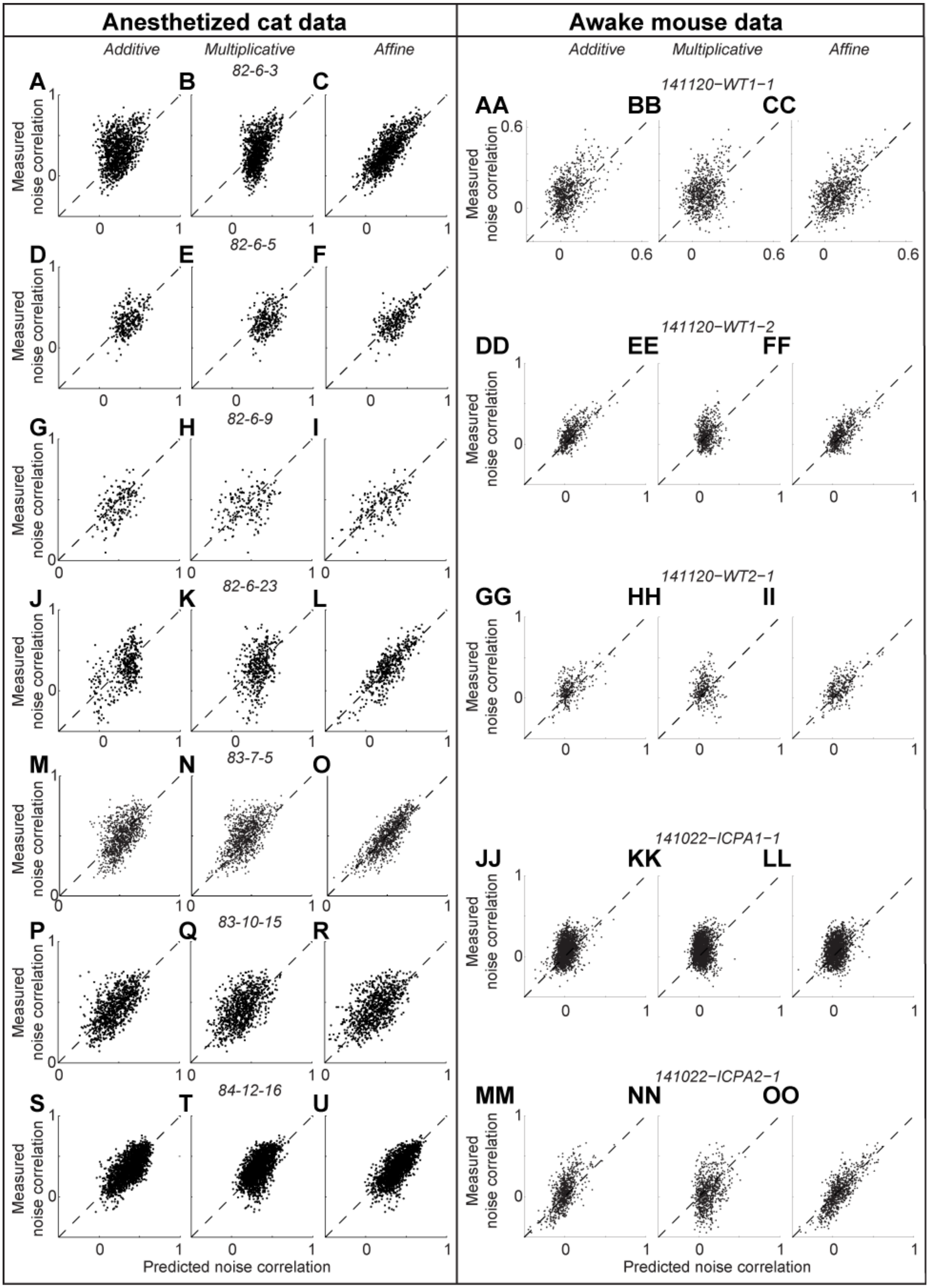

Anesthetized cat data
Awake mouse data
Additive
Multiplicative
Affine
82-6-3
82-6-5
82-6-9
82-6-23
83-7-5
83-10-15
84-12-16
141120−WT1−1
141120−WT1−2
141120−WT2−1
141022−ICPA1−1
141022−ICPA2−1
A
B
C
D
E
F
G
H
I
J
K
L
M
N
O
P
Q
R
S
T
U
AA
BB
CC
DD
EE
FF
GG
HH
II
JJ
KK
LL
MM
NN
OO
Measured noise correlation
Predicted noise correlation

Figure S5 (related to Figures 4 and 6). The affine model provides a better prediction of noise correlations in both anesthetized cat and quietly awake mouse data. (**A**-**C**) Measured noise correlations versus predictions from the additive (A), multiplicative (B), and affine (C) models for each pair of orientation-tuned sites in response to single gratings in anesthetized cat data. A *black dot* represents noise correlation for a pair of sites. Session 82-6-3, 47 orientation-tuned sites. (**D**-**F**) As in A-C, but for session 82-6-5, 24 orientation-tuned sites. (**G**-**I**) As in A-C, but for session 82-6-9, 19 orientation-tuned sites. (**J**-**L**) As in A-C, but for session 82-6-23, 29 orientation-tuned sites. (**M**-**O**) As in A-C, but for session 83-7-5, 45 orientation-tuned sites. (**P**-**R**) As in A-C, but for session 83-10-15, 42 orientation-tuned sites. (**S**-**U**) As in A-C, but for session 84-12-16, 64 orientation-tuned sites. (**AA-CC**) Measured noise correlations versus predictions from the additive (AA), multiplicative (BB), and affine (CC) models for each pair of neurons in response to single gratings in quietly awake mouse data. A *black dot* represents noise correlation for a pair of neurons. Session 141120-WT1-1, 39 stable, well-isolated neurons. (**DD**-**FF**) As in AA-CC, but for session 141120-WT1-2, 33 well-isolated neurons. (**GG**-**II**) As in AA-CC, but for session 141120-WT2-1, 29 well-isolated neurons. (**JJ**-**LL**) As in AA-CC, but for session 141022-ICPA1-1, 66 well-isolated neurons. (**MM**-**OO**) As in AA-CC, but for session 141022-ICPA2-1, 39 well-isolated neurons.

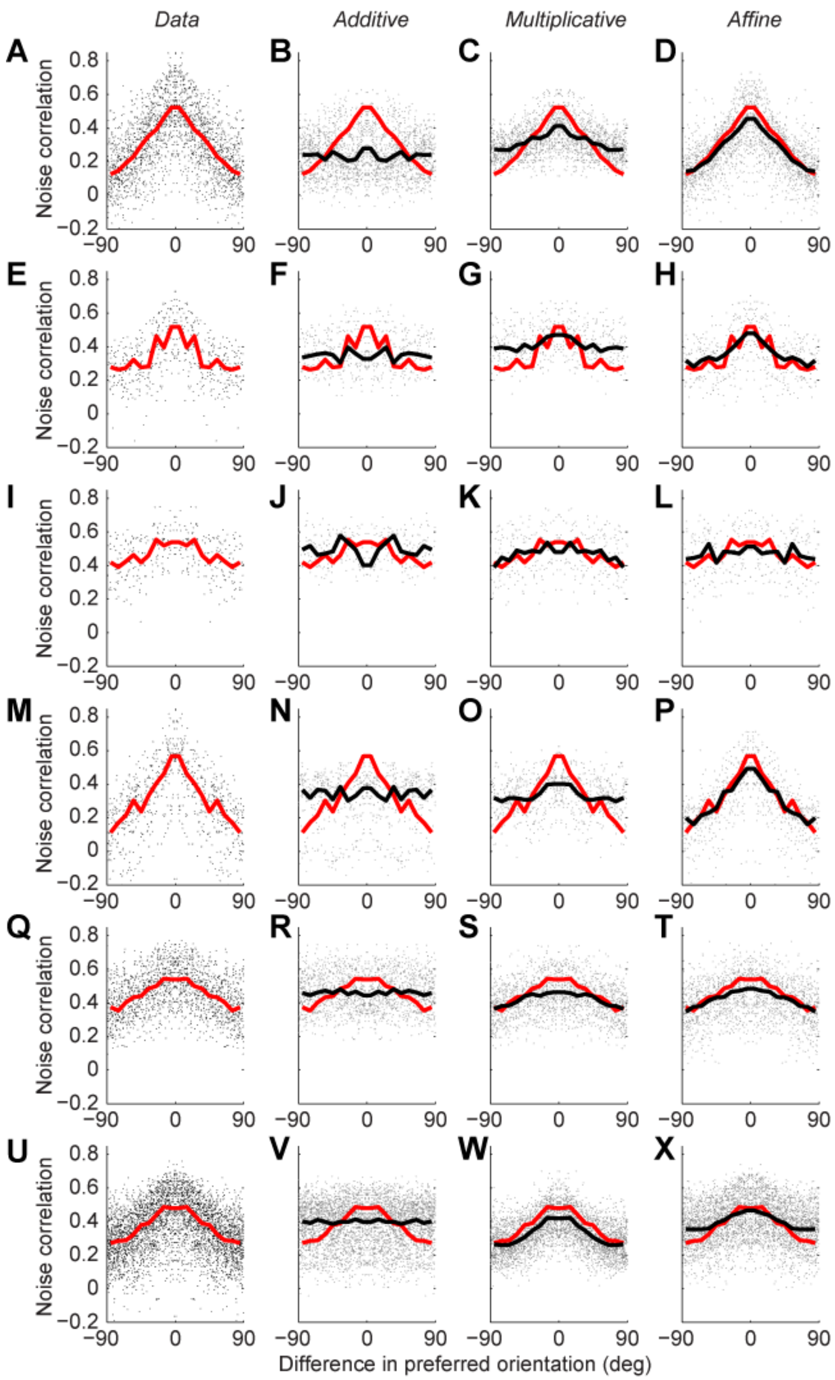

Figure S6 (related to Figure 4). Relationship between noise correlation and tuning similarity in anesthetized cat data. (**A**) Noise correlation for each pair of orientation-tuned sites in response to single gratings as a function of the difference between their preferred orientations. A *black dot* represents noise correlation calculated for a pair of sites, and the *red curve* shows the running median. (**B**-**D**) As in A, but for predictions of the additive (B), multiplicative (C), and affine (D) models. A *gray dot* represents noise correlation predicted by the model for a pair of sites, and a *black curve* shows the running median; a *red curve* repeats the running median of the measured data for comparison. All running medians were calculated with non-overlapping 10° bins. Session 82-6-3, 47 orientation-tuned sites. (**E**-**H**) As in A-D, but

for session 82-6-5, 24 orientation-tuned sites. (**I**-**L**) As in A-D, but for session 82-6-9, 19 orientation-tuned sites. (**M**-**P**) As in A-D, but for session 82-6-23, 29 orientation-tuned sites. (**Q**-**T**) As in A-D, but for session 83-10-15, 42 orientation-tuned sites. (**U**-**X**) As in A-D, but for session 84-12-16, 64 orientation-tuned sites.

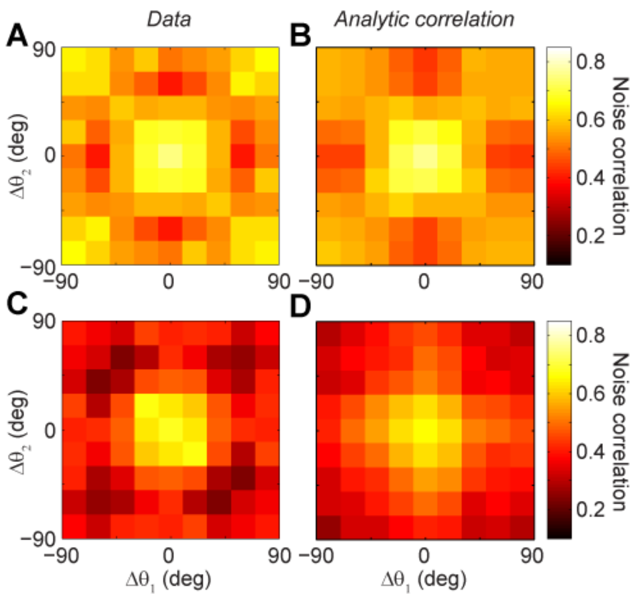

Figure S7 (related to Figure 5). The structures of correlation matrices can be analytically predicted from parameters of the affine model. (**A**) Experimentally measured correlation matrices for recording session 83-7-5 of anesthetized cat data. Here we only considered responses to single gratings at 50% contrast. (**B**) Correlation matrices computed from Eqs. S12, S15, and S16, with the parameters $d_{c,s}$, h_c, $F_{c,s}$, and statistical quantities $E(g_i)$, $Var(g_i)$, $E(a_i)$, $Var(a_i)$, $Cov(a_i, g_i)$ estimated from session 83-7-5. (**C**,**D**) Same as A,B, but for session 83-10-15.

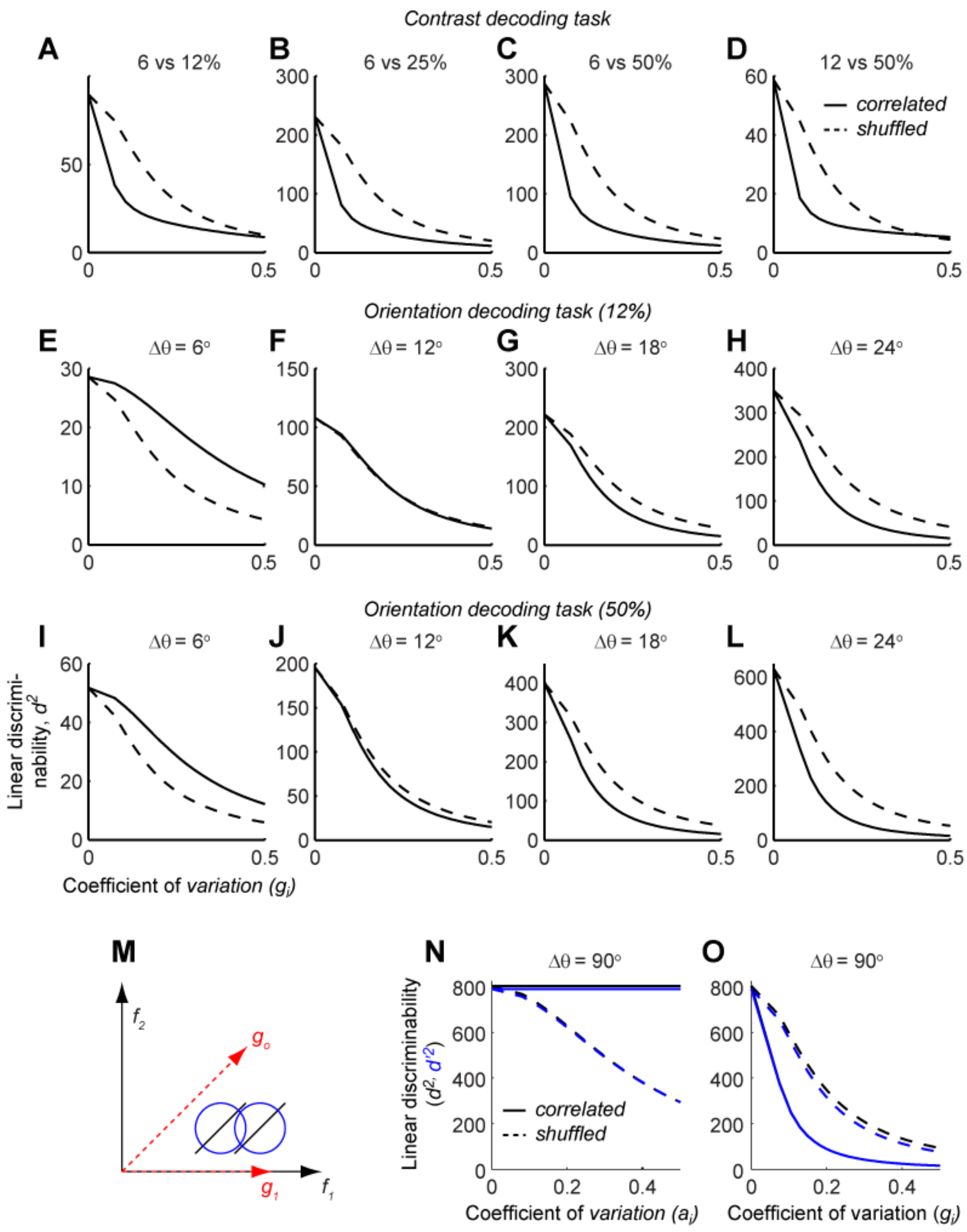

Figure S8 (related to Figures 7 and 8). Further analyses of the effects of fluctuations on stimulus coding. (**A-D**) The discriminability measure d^2 (*solid line*) and the corresponding d^2_{shuffled} (*dashed line*) as a function of the coefficient of variation of the multiplicative gain in four different contrast decoding tasks: two gratings of the same orientation at 6% and 12% (A), 6% and 25% (B), 6% and 50% (C), and 12% and 50% (D) contrasts. Note that trial-shuffling improves discrimination performance for all comparisons. (**E-H**) As in A-D, but for orientation discrimination tasks that involve distinguishing two 12%-contrast gratings whose orientations differed by 6° (E), 12° (F), 18° (G), and 24° (H). (**I-L**) As in E-H, but for tasks involving discerning two 50%-contrast gratings whose orientations differed by 6° (I), 12° (J), 18° (K), and 24° (L). Note that in orientation discrimination tasks, regardless of the stimulus contrast, trial-shuffling worsens discriminability for fine orientation discrimination (see also Figure 8E), but improves it for angles greater

than 12°. (**M**) Cartoon illustrating how shuffling of additive fluctuations worsens discriminability in a contrast discrimination task. The *x*- and *y*-axes represent the spike counts of two neurons, with f_1 representing a neuron that responds to the presented stimulus, and f_2 representing a neuron that responds to a different stimulus but is still modulated by additive variability. (For simplicity, here we consider the expected spike counts without passing through the spike count generator.) The vectors g_0 and g_1 correspond to an additive baseline shift and the tuned response to the grating. The two *black lines* show the distributions of responses to stimuli 1 and 2 if additive-correlated variability is present. Note that this variability is in a different direction to the difference between the mean stimulus responses, yielding two lines that are both parallel to g_0. After shuffling, these distributions become circular (*blue circles*), introducing a considerable overlap between the two distributions, thus reducing d^2. (**N**,**O**) Comparison between the linear discriminability measure d^2 (*black solid line*) as well as d^2_{shuffled} (*black dashed line*) calculated in the full high-dimensional response space and their corresponding approximations d'^2 (*blue solid line*) as well as d'^2_{shuffled} (*blue dashed line*) calculated in the 2-dimensional spaces used for visualization in Figures 7 and 8. Examples shown here are from one of the orientation discrimination tasks plotted in Figure 8. The similar shapes of the blue and black curves indicate that the projections illustrated in Figures 7 and 8 accurately capture the separation of high-dimensional ellipsoids in the full response space.

Session ID	*nu*	*nrpt*	*plaid angle*	*nu'*	*p*: additive vs multiplicative	*p*: additive vs affine	*p*: multiplicative vs affine
82-6-3	47	10	90°	46	5.4×10^{-4} (multiplicative)	2.8×10^{-14} (affine)	1.3×10^{-12} (affine)
82-6-5	24	10	30°	24	6.6×10^{-3} (multiplicative)	6.6×10^{-3} (affine)	2.8×10^{-4} (affine)
82-6-9	19	8	45°	19	1.7×10^{-1}	1.9×10^{-2} (affine)	7.6×10^{-5} (affine)
82-6-23	29	10	45°	29	2.4×10^{-2} (multiplicative)	3.7×10^{-9} (affine)	3.7×10^{-9} (affine)
83-7-5	45	10	90°	45	3.7×10^{-1}	2.6×10^{-12} (affine)	5.7×10^{-14} (affine)
83-10-15	42	7	90°	42	2.7×10^{-4} (multiplicative)	4.4×10^{-7} (affine)	6.9×10^{-5} (affine)
84-12-16	64	6	60°	60	1.1×10^{-3} (multiplicative)	6.3×10^{-14} (affine)	9.7×10^{-11} (affine)

Table S1 (related to Figure 2). Session ID, number of orientation-tuned units (*nu*), number of repeats per stimulus (*nrpt*), plaid angle, number of sites that showed considerable shared variability (*nu'*, quality index $q > 0.1$ for at least one of the models in cross-validation analysis) in each of the 7 recording sessions in anesthetized cat data. The last 3 columns tabulated the *p* value of the two-sided sign test on quality index of each model in cross-validation analysis. For ones that have $p < 0.05$, the model that has a greater median *q* (i.e., the better model) is indicated in bracket.

Session ID	*p*: additive vs extended multiplicative	*p*: multiplicative vs extended multiplicative	*p*: affine vs extended multiplicative
82-6-3	5.4×10^{-4} (extended multi)	1.6×10^{-3} (extended multi)	4.6×10^{-10} (affine)
82-6-5	6.6×10^{-3} (extended multi)	2.8×10^{-4} (extended multi)	2.3×10^{-2} (affine)
82-6-9	7.6×10^{-5} (extended multi)	7.3×10^{-4} (extended multi)	1
82-6-23	2.3×10^{-3} (extended multi)	6.1×10^{-2}	1.6×10^{-6} (affine)
83-7-5	2.5×10^{-3} (extended multi)	3.1×10^{-6} (extended multi)	9.3×10^{-9} (affine)
83-10-15	5.6×10^{-9} (extended multi)	7.9×10^{-3} (extended multi)	4.4×10^{-2} (affine)
84-12-16	1.6×10^{-7} (extended multi)	2.7×10^{-2} (extended multi)	1.2×10^{-5} (affine)

Table S2 (related to Figure 2). Session ID and the *p* value of the two-sided sign test on quality index of each model versus the extended multiplicative model in cross-validation analysis in each of the 7 recording sessions in anesthetized cat data. For ones that have $p < 0.05$, the model that has a greater median *q* (i.e., the better model) is indicated in bracket. Number of neurons that showed considerable shared variability (quality index $q > 0.1$ in for at least one of the models in cross-validation analysis) for each session was as in Table S1.

Session ID	*nu*	*nrpt*	*nu'*	*p*: additive vs multiplicative	*p*: additive vs affine	*p*: multiplicative vs affine
141120-WT1-1	39	35	22	5.3×10^{-2}	4.3×10^{-3} (affine)	8.6×10^{-4} (affine)
141120-WT1-2	33	50	21	7.8×10^{-2}	1.5×10^{-3} (affine)	2.2×10^{-4} (affine)
141120-WT2-1	29	35	18	2.4×10^{-1}	7.5×10^{-3} (affine)	1.3×10^{-3} (affine)
141022-PA1-1	66	15	26	8.5×10^{-1}	5.3×10^{-4} (affine)	1.0×10^{-5} (affine)
141022-PA2-1	39	15	27	1.2×10^{-1}	4.9×10^{-5} (affine)	4.9×10^{-5} (affine)

Table S3 (related to Figure 6). Session ID, number of stable, well-isolated neurons (*nu*), number of repeats per stimulus (*nrpt*), and number of neurons that showed considerable shared variability (*nu'*, quality index $q > 0.1$ in for at least one of the models in cross-validation analysis) in each of the 5 recording sessions in quietly awake mice. The last 3 columns tabulated the *p* value of the two-sided sign test on quality index of each model in cross-validation analysis. For ones that have $p < 0.05$, the model that has a greater median *q* (i.e., the better model) is indicated in bracket.

Session ID	*nu'*	*p*: additive vs extended multiplicative	*p*: multiplicative vs extended multiplicative	*p*: affine vs extended multiplicative
141120-WT1-1	22	2.9×10^{-1}	1	1.7×10^{-2} (affine)
141120-WT1-2	22	1.7×10^{-2} (extended multi)	1.7×10^{-2} (extended multi)	2.9×10^{-1}
141120-WT2-1	19	1	6.4×10^{-2}	4.4×10^{-3} (affine)
141022-PA1-1	30	6.0×10^{-5} (extended multi)	3.3×10^{-4} (extended multi)	8.6×10^{-1}
141022-PA2-1	27	5.9×10^{-3} (additive)	2.5×10^{-1}	1.5×10^{-3} (affine)

Table S4 (related to Figure 6). Session ID, number of neurons that showed considerable shared variability (*nu'*, quality index $q > 0.1$ in for at least one of the models in cross-validation analysis), and the *p* value of the two-sided sign test on quality index of each model versus the extended multiplicative model in cross-validation analysis in each of the 5 recording sessions in quietly awake mouse data. For ones that have *p* < 0.05, the model that has a greater median *q* (i.e., the better model) is indicated in bracket.

Supplemental Experimental Procedures

Anesthetized cat recordings

The data consisted of 7 recording sessions from 3 different neuronal populations in 3 cats (Busse et al., 2009). In short, young adult cats were anesthetized with ketamine and xylazine for initial surgery, followed by sodium pentothal and fentanyl for electrophysiological recordings. Responses were recorded from a 10-by-10 electrode array (Figure 1A, 400 μm spacing, 1.5 mm electrode length) with a Cerebus data acquisition system (Blackrock Microsystems, USA). The recordings were estimated to be primarily from layers 2/3 based on insertion depth.

Spikes were detected independently for each site of the array. For each site, we computed a threshold of 4 SD of the background noise. All threshold crossings on each channel were pooled, and only sites that showed orientation tuning were considered in subsequent analyses. Two criteria were used: first, the circular variance of the site's orientation tuning needed to be less than 0.9; second, the site's response (raw spike counts) to its preferred orientation needed to be at least twice that to the orthogonal stimulus at 100% contrast. On average, 42 ± 13 (median ± MAD) sites per recording passed the criteria.

Blanks were gray screen periods with the same mean luminance as the gratings. The gratings were circular (30° diameter), varying in orientation (6 orientations) and contrast (6%, 12%, 25%, and 50%), and of 4 Hz temporal frequency. Spatial frequency was adjusted to optimally drive the majority of sites in the array. Plaids were generated by summing two gratings. The plaid angle (the angle between the two component orientations) was fixed in each session, and plaids in each session consisted of three pairs of orientations (e.g., $0^o/90^o$ and $30^o/120^o$ for a plaid angle of 90°) to reduce the effect of adaptation. To obtain orientation tuning curves, responses to 100% contrast gratings of 12 different orientations were recorded. Stimuli were shown in random order in blocks and presented at least 6 times (Table S1).

Awake mouse recordings

We recorded from two C57BL/6J mice (8 week-old males) and two PV-Cre x Ai32 transgenic mice (11 week-old males; no optogenetic stimuli were applied for these experiments). Each mouse was implanted with a custom-built head plate with a recording chamber under isoflurane anesthesia. After 3 days of recovery accompanied by Rimadyl treatment and 3 head-restraint acclimatization sessions, the animal was briefly anesthetized with isoflurane and a ~1mm^2 craniectomy was made over the left V1 on the first recording day. The dura was resected with a 30G needle when necessary, and the brain was covered with cortex buffer and sealed using Kwik-Cast (World Precision Instruments, Inc.). If an additional recording was carried out the following day, the brain was again protected with cortex buffer and Kwik-Cast. There were five recording sessions in total, of which three were in the wild-type mice and two in the transgenic mice. Recordings were made with a Buzsaki32 silicon probe (NeuroNexus technologies, USA) that was lowered to a depth of 500-800 μm (median 615 μm) by a PatchStar manipulator (Scientifica, UK). Signals were amplified and stored for offline analysis using the Cerebus data acquisition system. During recording, the animal stood in a custom-built tube and were judged to be quietly awake by video monitoring (open eyes, sporadic whisking, postural adjustments, and other movements).

Detailed analysis was carried out only on well-isolated units (isolation distance > 20; refractory violation probability < 0.001 (Harris et al., 2001; Schmitzer-Torbert et al., 2005)) that showed consistent firing throughout a recording session (rate > 1 spike/trial). On average, 39 ± 6 (median ± MAD) neurons per session passed the criteria.

Stimuli were sequences of 1 s oriented drifting gratings (100% contrast, 2 Hz, 12 directions, and spatial frequency of 0.05 cpd), interspersed with either 1 s or 6 s blanks. Stimuli were presented 15-50 times in random order in blocks (Table S3). For sessions 141022-PA1-1 and 141022-PA1-2, stimuli of 60% contrast were also presented.

Population tuning curve analysis

To characterize population responses to plaid stimuli, we first computed each site's total spike count and firing rate on each trial. We then normalized each site's firing rate by dividing by the maximum of its fitted orientation tuning curve. Finally, we combined the results into a population response vector $\boldsymbol{r_i}$ that summarized the normalized firing rates of all orientation-tuned sites on trial i. We used least-squares estimation to fit a population tuning curve $\boldsymbol{R}_i$ to the measured data $\boldsymbol{r_i}$ on trial i as a linear combination of prototypical responses to the component gratings, plus a constant baseline shift:

$$\boldsymbol{R}_i = \alpha_1^i \boldsymbol{G}(\theta_1^i) + \alpha_2^i \boldsymbol{G}(\theta_2^i) + \beta^i \mathbf{1}. \quad \text{(S1)}$$

θ_1^i and θ_2^i are the component orientations of the plaid stimulus presented on trial i, $\boldsymbol{G}(\theta)$ represents the prototypical response to a grating of orientation θ, $\mathbf{1}$ represents the constant vector (all entries 1), and α_1^i, α_2^i, and β^i are weights estimated on trial i. The function $\boldsymbol{G}(\theta)$ was estimated from all presentations of single-grating stimuli as a unit circular Gaussian whose vertical offset and width are set as the baseline and the width of the mean single-site orientation response profile averaged across all orientation-tuned sites. Using this formula, the population tuning curve on trial i can be summarized using only three parameters: α_1^i, α_2^i, and β^i (Figure 1B).

The models

We denote the experimentally measured spike counts of unit c (single neuron or multiunit) on trial i as $N_{c,i}$ and the stimulus presented on trial i as $s(i)$. The independent, additive, multiplicative, and affine models predict an expected spike count $f_{c,i}$ of unit c on trial i that approximates $N_{c,i}$.

In the additive model (Figure 2B), the expected spike count is

$$f_{c,i} = d_{c,s(i)} + a_i h_c. \quad \text{(S2)}$$

The matrix $d_{c,s}$ (of size $M_{\text{units}} \times M_{\text{stimuli}}$) is estimated as the mean response of unit c over all presentations of stimulus s. All of the $M_{\text{units}} \times M_{\text{stimuli}}$ elements of this matrix represent parameters of the model that are fit to the data. The matrix $d_{c,s(i)}$ that appears in Eq. S2 has size $M_{\text{units}} \times M_{\text{trials}}$, and is generated by concatenating the columns of $d_{c,s}$ in the order $s(i)$. The M_{trials}-dimensional vector a_i represents the amount of common additive offset on each trial i, and the M_{units}-dimensional vector h_c represents the degree to which each unit c is susceptible to this offset. The parameters a_i and h_c were fit by least-squares, computed by keeping the first term in a singular value decomposition of the residual matrix $N_{c,i} - d_{c,s(i)}$.

In the multiplicative model (Figure 2C), the expected spike count is given by

$$f_{c,i} = g_i d_{c,s(i)}. \quad \text{(S3)}$$

The M_{trials}-dimensional vector g_i is the amount of common multiplicative gain on each trial i. The parameters g_i and $d_{c,s(i)}$ were fit by least-squares: for every stimulus s, we estimated the M_{units}-dimensional vector corresponding to the sth column of $d_{c,s}$ and the M_{repeats}-dimensional subvector of g_i

corresponding to the repeats of stimulus s by keeping the first term in a singular value decomposition of the $M_{\text{units}} \times M_{\text{repeats}}$-sized submatrix of $f_{c,i}$ that contains the observed spike counts in response to all repeats of stimulus s.

The affine model (Figure 2D) includes both the additive and multiplicative components:

$$f_{c,i} = g_i d_{c,s(i)} + a_i h_c. \tag{S4}$$

We fit the affine model by an alternation method. The additive terms a_i and h_c were computed as the first term in the singular value decomposition of the residual matrix $N_{c,i} - g_i d_{c,s(i)}$; the multiplicative terms g_i and $d_{c,s(i)}$ were then fit as the first term in the singular value decomposition of the residual $N_{c,i} - a_i h_c$ according to the method described for the multiplicative model.

Note that the parameterization of the affine model is underdetermined. Specifically, given any fit of this model, one can generate an equivalent fit for any real number γ:

$$d_{c,s(i)} = d'_{c,s(i)} - \gamma h_c, \tag{S5}$$

$$a_i = a'_i + \gamma g_i. \tag{S6}$$

To remove this ambiguity we chose the unique value of γ for which d_{c,s_0} (i.e., the modeled sensory drive induced by blanks) was the average spontaneous activity of unit c in a blank trial.

We found that the matrices $d_{c,s}$ fit for the additive, multiplicative, and affine models were very similar (the same also held true for h_c fit for the additive and affine models; data not shown).

Spike count generator

The model predictions are not integers, and they represent expected spike counts rather than actual potential observations. It is common in neuronal response modeling to simulate actual spike counts according to a Poisson distribution, the variability of which captures "private noise" individual to each neuron. Nevertheless, there is nothing in neuronal biophysics that suggests neurons should produce Poisson variability, and a Poisson distribution may underestimate the amount of private variability in each neuron. To generate spike counts $n_{c,i}$ from the model, we thus used a negative binomial distribution, a more general family of count distributions to which the Poisson belongs:

$$n_{c,i} \sim NB\left(f_{c,i}, F_{c,s(i)}\right). \tag{S7}$$

The negative binomial distribution has two parameters and can be characterized by an expected spike count f and Fano factor F. (Note that the negative binomial distribution can be parameterized in multiple ways; another common parameterization would be $p = 1 - \frac{1}{F}$ and $r = \frac{f(1-p)}{p}$.) The negative binomial reduces to a Poisson distribution when F is 1, but it has higher variance than Poisson for $F > 1$. After computing the expected spike counts $f_{c,i}$ as described above, we estimated a Fano factor $F_{c,s}$ for each unit c and stimulus s by maximum likelihood. Specifically, we used the simplex algorithm ('fminsearch' function in Matlab) to search for the value of $F_{c,s}$ that minimized the negative log likelihood of the full set of observed spike counts.

Cross-validation

We assessed the models' goodness of fit by cross-validating the simulated spike count $n_{c,i}$ (Figure S1). Because of the way the model was parameterized, this required a specialized approach. Specifically, we

looped over all units and trials to predict the response of unit c on trial i from all other elements of the spike count matrix. Unit c is termed the 'test unit', and trial i is termed the 'test trial'. The remaining units were referred to as 'training units', and the remaining trials as 'training trials'. The data from all training trials was used to estimate the stimulus-drive and fluctuation-coupling parameters $\tilde{d}_{c,s(i)}$ and $\tilde{h}_c$ as well as the unit- and stimulus-dependent Fano factor $\tilde{F}_{c,s(i)}$. Then, the multiplicative gain $\tilde{g}_i$ and additive offset $\tilde{a}_i$ on the test trial were estimated from the activity of all training units on this trial. Finally, a prediction $\tilde{f}_{c,i} = \tilde{g}_i\tilde{d}_{c,s(i)} + \tilde{a}_i\tilde{h}_c$ was obtained for the expected spike count of the test unit on the test trial. Note that this prediction used all other elements of the response matrix except the unit and trial being predicted. To obtain more accurate results, we analytically compute the expected cross-validation error over the distribution produced by the spike count generator of Eq. S7 (i.e., the private variability), while considering a fixed value of $f_{c,i}$ and $N_{c,i}$ as well as with all expectations and variances defined by the probability distribution of Eq. S7. The squared error with respect to the test unit c on test trial i can be computed as:

$$\begin{aligned} e_{c,i}^2 &= E\left(\left(n_{c,i} - N_{c,i}\right)^2\right) \\ &= E\left(\left(n_{c,i} - E\left(n_{c,i}\right)\right)^2\right) + \left(E\left(n_{c,i}\right) - N_{c,i}\right)^2 \\ &= Var\left(n_{c,i}\right) + \left(E\left(n_{c,i}\right) - N_{c,i}\right)^2 \\ &= F_{c,s}E\left(f_{c,i}\right) + Var\left(f_{c,i}\right) + \left(f_{c,i} - N_{c,i}\right)^2 \\ &= F_{c,s}f_{c,i} + \left(f_{c,i} - N_{c,i}\right)^2, \end{aligned} \quad (S8)$$

using Eq. S12 and the fact that $Var\left(f_{c,i}\right) = 0$ (since here we consider $f_{c,i}$ as a fixed value). The first term in the last line is the contribution from the stochastic spike count generator to the squared error; the second term is the error from the spike estimate derived from each of the models.

Analytic calculation of pairwise correlations

Here we compute the correlations expected from the affine model. In this calculation, in addition to stochastic spike generation (private variability, Eq. S7), we consider g_i and a_i to be random variables; the only aspects of their probability distributions that make a difference to the pairwise neuronal correlations are their means, variances, and covariances.

To analytically derive the correlations expected under this model, we first compute the expected value and variance of $f_{c,i}$ during repeated presentations of stimulus s:

$$E\left(f_{c,i}\right) = E(g_i)d_{c,s} + E(a_i)h_c, \quad (S9)$$

$$Var\left(f_{c,i}\right) = Var(g_i)d_{c,s}^2 + Var(a_i)h_c^2 + 2Cov(g_i, a_i)d_{c,s}h_c. \quad (S10)$$

Similarly, the covariance of the expected spike counts for two units is

$$Cov\left(f_{c_1,i}, f_{c_2,i}\right) = Var(g_i)d_{c_1,s}d_{c_2,s} + h_{c_1}h_{c_2}Var(a_i) + \left(h_{c_1}d_{c_2,s} + h_{c_2}d_{c_1,s}\right)Cov(g_i, a_i). \quad (S11)$$

We next compute the variance of spike counts $n_{c,i}$ for unit c. To do this, we employ the law of total variance, which holds that for random variables X and Y, $Var(Y) = E\left(Var(Y|X)\right) + Var(E(Y|X))$. Using this with $X = f_{c,i}$ and $Y = n_{c,i}$, we have

$$Var\left(n_{c,i}\right) = E\left(Var\left(n_{c,i}|f_{c,i}\right)\right) + Var\left(E\left(n_{c,i}|f_{c,i}\right)\right) = F_{c,s}E\left(f_{c,i}\right) + Var\left(f_{c,i}\right). \quad (S12)$$

All variances and expectations are over repeated presentations of a single stimulus s. This can be interpreted as a sum of two terms: the first corresponding to the amount of private variance expected due to the neuron's spike generator, and the second corresponding to the amount of variance expected due to shared population variability.

Similarly, to compute the covariance of two cells c_1 and c_2, we employ the law of total covariance: $Cov(X,Y) = Cov\big(E(X|Z),E(Y|Z)\big) + E\big(Cov(X,Y|Z)\big)$. To use this formula, we let X denote $n_{c_1,i}$, let Y denote $n_{c_2,i}$, and let Z denote the compound random variable $\{f_{c_1,i}, f_{c_2,i}\}$, then we have $E(X|Z)$=$f_{c_1,i}$, $E(Y|Z)$=$f_{c_2,i}$, and

$$Cov\big(E(X|Z),E(Y|Z)\big) = Cov\big(f_{c_1,i}, f_{c_2,i}\big). \tag{S13}$$

To compute $E\big(Cov(X,Y|Z)\big)$, we note that the spike generator of each neuron is conditionally independent. Thus,

$$\begin{aligned} &E\left(Cov\big(n_{c_1,i}, n_{c_2,i}\big|f_{c_1,i}, f_{c_2,i}\big)\right) \\ &= \delta_{c_1c_2}E\left(Var\big(n_{c_1,i}\big|f_{c_1,i}\big)\right) \\ &= \delta_{c_1c_2}E\big(F_{c_1,s}f_{c_1,i}\big) \\ &= \delta_{c_1c_2}F_{c_1,s}\big(E(g_i)d_{c_1,s} + E(a_i)h_{c_1}\big). \end{aligned} \tag{S14}$$

And the covariance of n_{c_1} and n_{c_2} is given by:

$$\begin{aligned} Cov\big(n_{c_1,i}, n_{c_2,i}\big) = Var(g_i)d_{c_1,s}d_{c_2,s} + h_{c_1}h_{c_2}Var(a_i) + \big(h_{c_1}d_{c_2,s} + h_{c_2}d_{c_1,s}\big)Cov(g_i,a_i) \\ + \ \delta_{c_1c_2}F_{c_1,s}\big(E(g_i)d_{c_1,s} + E(a_i)h_{c_1}\big). \end{aligned} \tag{S15}$$

This equation may be understood as a matrix containing the covariance of the expected spike counts, $f_{c,i}$, plus an additional diagonal term corresponding to each neuron's private variability as produced by the spike generator.

To compute the Pearson correlation, we substitute the results of Eqs. S12 and S15 into the formula

$$\rho\big(n_{c_1,i}, n_{c_2,i}\big) = \frac{Cov\big(n_{c_1,i}, n_{c_2,i}\big)}{\sqrt{Var\big(n_{c_1,i}\big)Var\big(n_{c_2,i}\big)}}. \tag{S16}$$

The correlation of any two units $\rho\big(n_{c_1,i}, n_{c_2,i}\big)$ can therefore be computed analytically in terms of the parameters $d_{c,s}$ and h_c and the statistical quantities $E(g_i), Var(g_i), E(a_i), Var(a_i),$ and $Cov(g_i,a_i)$. Examples of predictions of this analytical model are depicted in Figure S7.

Decoding

To estimate how d^2 depended on the fluctuations of additive offset and multiplicative gain, we constructed a homogeneous neural population with translation-invariant orientation-tuning curves (36 neurons whose preferred orientations are 5° apart). We took $d_{c,s}$ as a circular Gaussian function of width 15° and h_c as a constant value of 600 (both approximated from one example session of anesthetized cat data). The Fano-factor parameter $F_{c,s}$ for the spike count generator was fixed at 1.7 for all stimuli and cells. We investigated how d^2 depended on the values of $E(a_i)$, $E(g_i)$, $Var(a_i)$, and $Var(g_i)$ (within biologically plausible ranges estimated from anesthetized cat data) by using analytic formulae to evaluate

$\bar{\boldsymbol{n}}_1$, $\bar{\boldsymbol{n}}_2$, $Cov(\boldsymbol{n}_1)$, and $Cov(\boldsymbol{n}_2)$ (Supplemental Experimental Procedures: analytic calculation of pairwise correlations). The parameters were varied one at a time; parameters that were not changed were held fixed at $E(a_i)$ = 0.055, $E(g_i)$ = 0.3, $Var(a_i)$ = 0, and $Var(g_i)$ = 0.

Decoding analysis with reduced dimensionality

In order to gain an intuitive understanding of the population coding results (Figures 7 and 8), it is helpful to project the high-dimensional responses into a 1- or 2-dimensional space. Specifically, given the spike count vector $\boldsymbol{n_i}$ on trial i, we can project it onto a low-dimensional space by matrix multiplication:

$$\boldsymbol{\alpha_i} = B\boldsymbol{n_i}. \tag{S17}$$

The models of population tuning curves shown in Figures 1 and 3 are examples of such a low-dimensional projection, with B given by the pseudoinverse of the matrix whose columns are the basis vectors $\boldsymbol{G}(\theta)$, corresponding to single-grating responses, and a column of ones that corresponds to the additive offset.

For the reduced-dimensional projection to faithfully represent discriminability in the high-dimensional space, two conditions are necessary. First, cluster separation in the low-dimensional space should be approximately equal to that in the full space. Discriminability in the low-dimensional space is given by

$$d'^2 = (B(\bar{\boldsymbol{n}}_2 - \bar{\boldsymbol{n}}_1))^{\mathrm{T}}\left(B\Sigma B^{\mathrm{T}}\right)^{-1}\left(B(\bar{\boldsymbol{n}}_2 - \bar{\boldsymbol{n}}_1)\right). \tag{S18}$$

$\bar{\boldsymbol{n}}_s$ is a vector containing the mean spike count of all units over all presentations of stimulus s, and Σ is the population covariance matrix averaged across the two stimuli to be discriminated. Note that if B were invertible, d'^2 would equal d^2. For low-dimensional projections, B is a non-invertible rectangular matrix. Nevertheless, for the projections used in Figures 7 and 8, d'^2 is a close approximate of d^2 (Figures S8N and S8O). The second criterion is that the projection is orthogonal. The projections of Figures 1 and 3 are not orthogonal: although the responses to two gratings 90° apart are close to orthogonal, each grating response is not orthogonal to the constant offset. When projecting onto basis vectors corresponding to gratings of similar orientation, this non-orthogonality becomes extreme, as the two grating responses are very similar. To visualize responses in this space (Figure 8), we therefore used the Gram-Schmidt process to orthonormalize the single-grating responses.

The extended multiplicative model

Variable coupling to population activity is included in the additive model, but not in the multiplicative model. To investigate whether this was the sole reason for the affine model's better performance, we introduce an "extended multiplicative model" that allows each neuron to be coupled to the population activity to different degrees (Figure S2A), and we ask if this additional coupling flexibility in the extended multiplicative model better explains the shared variability than the additive component in the affine model.

In this model, the expected spike count $f_{c,i}$ of unit c on trial i is given by

$$f_{c,i} = (1 - K_c)d_{c,s(i)} + g_i K_c d_{c,s(i)}. \tag{S19}$$

As before, $s(i)$ is the stimulus presented on trial i, $d_{c,s(i)}$ is the sensory drive to unit c from stimulus $s(i)$, g_i is the shared multiplicative gain on trial i, and the new term K_c represents the degree to which each unit c is coupled to this gain. For a unit with K_c = 0, its spiking activity is independent of its surrounding neuronal population, and the model reduces to the independent model. For a unit with K_c = 1, $d_{c,s(i)}$ is perfectly synched with the population activity, and its behavior can be described by the original

multiplicative model. g_i varies from trial to trial, whereas $d_{c,s(i)}$ and K_c take the same values on all trials. The number of parameters in this model ($M_{\text{units}}M_{\text{stimuli}} + M_{\text{units}} + M_{\text{trials}}$) is equivalent to the additive model.

To fit the model numerically, we first re-parametrized. Setting $k_c = \frac{1-K_c}{K_c}$ and $d'_{c,s(i)} = K_c d_{c,s(i)}$, we can rewrite Eq. S19 as

$$f_{c,i} = k_c d'_{c,s(i)} + g_i d'_{c,s(i)}, \tag{S20}$$

which in matrix form is

$$\begin{pmatrix} f_{c_1,1} \\ f_{c_1,2} \\ \vdots \\ f_{c1,M_{\text{trials}}} \\ f_{c_2,1} \\ f_{c_2,2} \\ \vdots \\ f_{c_2,M_{\text{trials}}} \\ \vdots \\ f_{c_{M_{\text{units}}},1} \\ f_{c_{M_{\text{units}}},2} \\ \vdots \\ f_{c_{M_{\text{units}}},M_{\text{trials}}} \end{pmatrix} = \begin{pmatrix} d'_{c_1,s(1)} & 0 & \cdots & 0 & d'_{c_1,s(1)} & 0 & \cdots & 0 \\ d'_{c_1,s(2)} & 0 & \cdots & 0 & 0 & d'_{c_1,s(2)} & \cdots & 0 \\ \vdots & \vdots & \vdots & \vdots & \vdots & \vdots & \vdots & \vdots \\ d'_{c_1,s(M_{\text{trials}})} & 0 & \cdots & 0 & 0 & 0 & \cdots & d'_{c_1,s(M_{\text{trials}})} \\ 0 & d'_{c_2,s(1)} & \cdots & 0 & d'_{c_2,s(1)} & 0 & \cdots & 0 \\ 0 & d'_{c_2,s(2)} & \cdots & 0 & 0 & d'_{c_2,s(2)} & \cdots & 0 \\ \vdots & \vdots & \vdots & \vdots & \vdots & \vdots & \vdots & \vdots \\ 0 & d'_{c_2,s(M_{\text{trials}})} & \cdots & 0 & 0 & 0 & \cdots & d'_{c_2,s(M_{\text{trials}})} \\ \vdots & \vdots & \vdots & \vdots & \vdots & \vdots & \vdots & \vdots \\ 0 & 0 & \cdots & d'_{c_{M_{\text{units}}},s(1)} & d'_{c_{M_{\text{units}}},s(1)} & 0 & \cdots & 0 \\ 0 & 0 & \cdots & d'_{c_{M_{\text{units}}},s(2)} & 0 & d'_{c_{M_{\text{units}}},s(2)} & \cdots & 0 \\ \vdots & \vdots & \vdots & \vdots & \vdots & \vdots & \vdots & \vdots \\ 0 & 0 & \cdots & d'_{c_{M_{\text{units}}},s(M_{\text{trials}})} & 0 & 0 & \cdots & d'_{c_{M_{\text{units}}},s(M_{\text{trials}})} \end{pmatrix} \begin{pmatrix} k_{c_1} \\ k_{c_2} \\ \vdots \\ k_{c_{M_{\text{units}}}} \\ g_1 \\ g_2 \\ \vdots \\ g_{M_{\text{trials}}} \end{pmatrix}.$$

This allows us to fit the extended multiplicative model to experimental data by an alternation method: given a current estimate of $d'_{c,s(i)}$, k_c and g_i are estimated via ridge regression with $\sqrt{\lambda} = 0.02$; then the scaled sensory drive term $d'_{c,s(i)}$ is again fit via ridge regression with $\sqrt{\lambda} = 0.02$. The alternation is repeated until a convergence criterion is met, specifically that the difference in squared error per unit per trial $\frac{\sum_{c,i}\left(f_{c,i}-N_{c,i}\right)^2}{M_{\text{units}} \times M_{\text{trials}}}$ between two iterations was less than 10^{-10}. This typically took few thousand iterations.

In all sessions of anesthetized cat recording, the extended multiplicative model outperformed both the additive model (Figure S2B & Table S2; $p < 10^{-24}$ for all data together and $p < 0.007$ in all recording sessions evaluated individually; sign test) and the multiplicative model (Figure S2C & Table S2; $p < 10^{-15}$ for all data together, $p < 0.008$ in 5 individual sessions and $p < 0.03$ in 1 session; sign test). Nevertheless, the affine model bested the extended multiplicative model (Figure S2D & Table S2; $p < 10^{-22}$ for all data together, $p < 10^{-4}$ in 4 individual sessions and $p < 0.05$ in 2 others; sign test). In the quietly awake mouse data, the extended multiplicative model sometimes performed better than the additive model (Figure S2E & Table S4; $p < 0.03$ for all data together, $p < 0.02$ in 3 individual sessions, out of which one was better predicted by the additive model; sign test) and the multiplicative model (Figure S2F & Table S4; $p < 0.004$ for all data together and $p < 0.02$ for 2 individual sessions; sign test). Yet again, the affine model performed considerably better than the extended multiplicative model (Figure S2G & Table S4; $p < 10^{-4}$ for all data together, $p < 0.02$ in 3 individual sessions; sign test). In addition, the extended multiplicative model – as the original multiplicative model - failed to capture all possible forms of the correlation matrix: the extended multiplicative model also tended to overestimate the edge-bin correlations while under-estimating the corner-bin correlations (Figures S2H-S2O). These results suggest that the additive com-ponent in the affine model could not be trivially explained away by each cell's coupling strength. While including a cell-coupling factor for the multiplicative gain in the full affine model might potentially improve

the model even further, estimating this model would present severe numerical difficulties, and it is outside the scope of present work.

Supplemental References

Harris, K.D., Hirase, H., Leinekugel, X., Henze, D.A., and Buzsaki, G. (2001). Temporal interaction between single spikes and complex spike bursts in hippocampal pyramidal cells. Neuron 32, 141-149.

Schmitzer-Torbert, N., Jackson, J., Henze, D., Harris, K., and Redish, A.D. (2005). Quantitative measures of cluster quality for use in extracellular recordings. Neuroscience 131, 1-11.
